# Synthesis and Biological Evaluation of Novel 2-Aroyl Benzofuran-Based Hydroxamic Acids as Antimicrotubule Agents

**DOI:** 10.3390/ijms25147519

**Published:** 2024-07-09

**Authors:** Elena Mariotto, Martina Canton, Chiara Marchioro, Andrea Brancale, Ernest Hamel, Katia Varani, Fabrizio Vincenzi, Tiziano De Ventura, Chiara Padroni, Giampietro Viola, Romeo Romagnoli

**Affiliations:** 1 Department of Woman’s and Child’s Health, Hemato-Oncology Lab, University of Padova, 35128 Padova, Italy; elena.mariotto@unipd.it (E.M.); martina.canton.1@studenti.unipd.it (M.C.); chiara.marchioro.1@studenti.unipd.it (C.M.); giampietro.viola.1@unipd.it (G.V.); 2 Laboratory of Experimental Pharmacology, Istituto di Ricerca Pediatrica (IRP), Fondazione Città della Speranza, 35128 Padova, Italy; 3 Department of Organic Chemistry, University of Chemistry and Technology, Prague, 166 28 Prague, Czech Republic; Andrea.Brancale@vscht.cz; 4 Molecular Pharmacology Branch, Developmental Therapeutics Program, Division of Cancer Treatment and Diagnosis, Frederick National Laboratory for Cancer Research, National Cancer Institute, National Institutes of Health, Frederick, MD 21702, USA; hamele@dc37a.nci.nih.gov; 5 Department of Translational Medicine, University of Ferrara, 44121 Ferrara, Italy; vrk@unife.it (K.V.); fabrizio.vincenzi@unife.it (F.V.); 6 Department of Chemical, Pharmaceutical and Agricultural Sciences, University of Ferrara, 44121 Ferrara, Italy; tiziano.deventura@unife.it; 7 Medicinal Chemistry Department, Integrated Drug Discovery, Aptuit, an Evotec Company, 37135 Verona, Italy; chiara.padroni@gmail.com

**Keywords:** dual-target inhibitors, anticancer agents, benzo[*b*]furan, tubulin, histone deacetylase (HDAC)

## Abstract

Because of synergism between tubulin and HDAC inhibitors, we used the pharmacophore fusion strategy to generate potential tubulin–HDAC dual inhibitors. Drug design was based on the introduction of a *N*-hydroxyacrylamide or a *N*-hydroxypropiolamide at the 5-position of the 2-aroylbenzo[*b*]furan skeleton, to produce compounds **6a**–**i** and **11a**–**h**, respectively. Among the synthesized compounds, derivatives **6a**, **6c**, **6e**, **6g**, **11a,** and **11c** showed excellent antiproliferative activity, with IC_50_ values at single- or double-digit nanomolar levels, against the A549, HT-29, and MCF-7 cells resistant towards the control compound combretastatin A-4 (CA-4). Compounds **11a** and **6g** were also 10-fold more active than CA-4 against the Hela cell line. When comparing the inhibition of tubulin polymerization versus the HDAC6 inhibitory activity, we found that **6a**–**g**, **6i**, **11a**, **11c**, and **11e**, although very potent as inhibitors of tubulin assembly, did not have significant inhibitory activity against HDAC6.

## 1. Introduction

The microtubule network is a key component of the cytoskeleton in eukaryotic cells and plays a crucial role in a wide range of cellular functions. The microtubule is thus a validated target for anticancer drug discovery [1,2]. Molecules that bind to tubulin are known as microtubule targeting agents (MTAs) and can be classified as either microtubule stabilizing agents (MSAs) or microtubule-destabilizing agents (MDAs) [3,4]. Treatment with MTAs interferes with spindle formation in mitosis and induces cell cycle arrest at the G2/M phase, followed by apoptosis of tumor cells [5]. To date, eight distinct binding sites of MTAs on tubulin have been identified, six of which are either entirely on β-tubulin or are at the αβ interface between adjacent tubulin dimers in a protofilament, with the pironetin site located on α-tubulin [6]. The taxane/epothilone site [7] and the laulimalide/peloruside site [8] on β-tubulin accommodate all known MSAs [9]. In terms of the MDAs, the colchicine site [10] is extremely important because it binds numerous structurally diverse compounds and is the most accessible for medicinal chemists. 

Among the eight distinct classes of MTA binding sites that have been identified, up to now, only three types of antimitotic agents have been approved for clinical use in treating cancer. These are the vinca alkaloids vinblastine and vincristine and the synthetic vinorelbine, the synthetic halichondrin B analogue eribulin, and several taxanes and the synthetic epothilone analogue ixabepilone. These agents are used in individual and combination therapies [11,12]. However, emerging multidrug resistance (MDR) mediated by the overexpression of membrane-bound drug efflux proteins [13], along with overexpression of the βIII tubulin isoform [14] and the expression of antiapoptotic proteins such as survivin [15], are the main mechanisms that have limited their use in clinical practice for long-term treatment [16]. In addition, current MTAs not only suppress and alter tubulin dynamics in cancer cells but also affect normal cells leading to neutropenia and peripheral neuropathy as the main adverse side effects [17].

**Figure 1:**
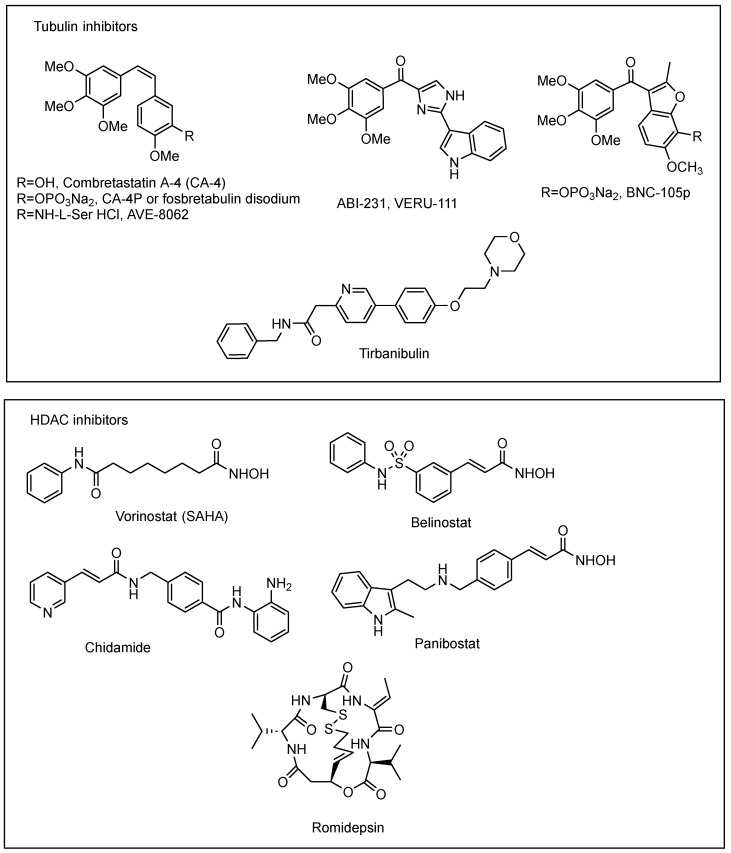
Chemical structures of representative tubulin inhibitors binding to the colchicine site and structures of approved HDAC inhibitors.

MTAs that bind to the colchicine site (colchicine-binding site inhibitors, or CBSIs), located at the interface between α- and β-tubulin heterodimers, have attracted the attention of many researchers interested in discovering novel tubulin targeted antitumor agents. Unlike other MTAs [18,19,20], CBSIs display several advantages, notably structural simplicity, anti-angiogenic effects, improved aqueous solubility, broad therapeutic index, and reduced tumor drug resistance, due to their ability to bind a range of β-tubulin isotypes and evade multidrug resistance-associated transporter proteins [21]. One of the most notable of these compounds, both for its potency and structural simplicity, is the naturally occurring polymethoxylated *cis*-stilbene derivative combretastatin A-4 (CA-4, Figure 1) [22]. Moreover, several studies have documented that this compound also acts as a powerful vascular disrupting agent [23], particularly for the neovasculature of tumors. To date, several CBSIs including the corresponding phosphate derivative of combretastatin A-4 (CA-4P, also known as fosbretabulin disodium) [24], BPR0L075 (SCB01A) [25], BNC-105p [26], AVE8062 [27], and sabizabulin (ABI-231, VERU-111) [28] have advanced to clinical trials for the treatment of various cancers such as colon cancer, breast cancer, lung cancer, and prostate cancer as found on the site “www.clinicaltrials.gov (accessed in March 2024)”. Tirbanibulin (KX2-391), a dual Src kinase signaling inhibitor and tubulin polymerization inhibitor acting at the colchicine site of β-tubulin [29], was recently approved by the Food and Drug Administration (FDA) for the treatment of actinic keratosis [30].

Histone deacetylases (HDACs) are a family of pleiotropic enzymes that catalyze the removal of acetyl groups from the lysine residues located in the N-terminal tails of histone proteins and thus promote a change in chromatin structure [31]. This change leads to the process of epigenetic regulation of gene transcription involved in numerous cellular functions, including proliferation, angiogenesis, apoptosis, and motility [32,33]. In cancer cells, HDACs are often aberrantly overexpressed, leading to histone hypoacetylation causing transcriptional repression of many tumor suppressor genes, such as p21 and p27 [34]. HDACs also regulate the acetylation state of non-histone proteins, such as α-tubulin, transcription factors, and heat shock protein 90 (Hsp90) [35]. Inhibition of HDACs is therefore regarded as an important target for cancer therapy [36]. 

Eighteen different HDAC subtypes have been identified in mammalian cells and have been further classified into four families [37]. Amongst them, class I (HDACs 1–3 and HDAC8), class II (HDACs 4–7 and HDACs 9–10) and class IV (HDAC 11) are zinc-dependent metalloenzymes [38] 

Currently, the US Food and Drug Administration (FDA) has approved four HDAC inhibitors (HDACis) for the treatment of hematologic cancers, such as T-cell lymphoma and multiple myeloma. These drugs are Vorinostat (SAHA) [39], Belinostat (PXD101) [40], Romidepsin (FK228) [41], and Panobinostat (LBH589) [42], while, in addition, Chidamide has been approved by China’s FDA to treat patients with recurrent or refractory peripheral T-cell lymphoma (PTCL) [43]. Unfortunately, many of the HDACis, when used as a single agent, showed dose-limiting adverse effects due to their relative lack of selectivity among the different HDAC isoforms (pan-HDACi) [44], and drug resistance was often observed [45]. In addition, HDACis showed a limited therapeutic application with efficacy in hematologic malignancies only and no significant effects against solid tumors [46]. Combination therapy of HDACis with other anticancer agents has shown synergistic or additive anticancer effects in clinical studies, achieving efficacy against solid tumors and reducing drug resistance [47,48]. 

α-Tubulin is an important cytosolic non-histone protein, and its acetylation is regulated by the HDAC6 isoform, thereby impacting the microtubule network [49]. The synergetic effect of histone deacetylases inhibitors combined with tubulin inhibitors such as taxanes and vincristine is an important strategy to improve therapeutic efficacy and overcome the resistance to single-target drug therapies [50,51,52]. However, combination therapy suffers several drawbacks, such as drug–drug interactions, unpredictable pharmacokinetic, and safety profiles with enhanced adverse effects and poor patient compliance [53]. 

To overcome the limitations of single-target agents or drug combinations, an increasing number of investigations have focused on the rational design of a single-molecule inhibitor of two targets, such as compounds capable of inhibiting tubulin and histone deacetylase simultaneously, thereby amplifying their antitumor effects [54,55,56].

### Rational Design of Novel Dual Tubulin–HDAC Inhibitors

A general three-component pharmacophore model for HDACis has been established, including a surface recognition cap (SRC) group occupying the entrance area to the active site, a zinc-binding group (ZBG) interacting with the zinc ion in the catalytic center and a hydrophobic flexible or rigid spacer/linker connecting the two units through the enzyme’s channel [57,58,59]. The ZBG and linker are crucial for HDAC inhibition as they are necessary to access and bind to the zinc ion in the internal cavity of the enzyme. The amino acids in these regions are highly conserved among the different HDAC isoforms. The cap group binds to a less conserved area around the rim at the entrance to the active site. HDACis have the ability to tolerate cap groups of diverse structures without affecting HDAC inhibitory capabilities significantly, and isoform selectivity may be attained by modifying the cap group [57].

The design strategy of HDACis with dual-acting capabilities typically requires combining in a unique framework the ZBG with a pharmacophore for another target as the SRC group via a linker [60].

While HDAC inhibition can be obtained from incorporation of a zinc binding group through a linker, tubulin activity requires an appropriate chemical scaffold that can accommodate the HDAC functionality without compromising potency of the compound as a tubulin polymerization inhibitor. As previously described, HDAC inhibitors can tolerate extensive structural modifications at the capping group [61], while ZBG and the linker are relatively conserved. Because the capping group locates outside of the binding channel of the HDAC and is not involved in critical contacts with the active site, many dual tubulin–HDAC inhibitors have a capping group mandated by the pharmacophore of the tubulin inhibitor attached to a ZBG from an HDACi via a rigid or flexible linker [62]. 

**Figure 2:**
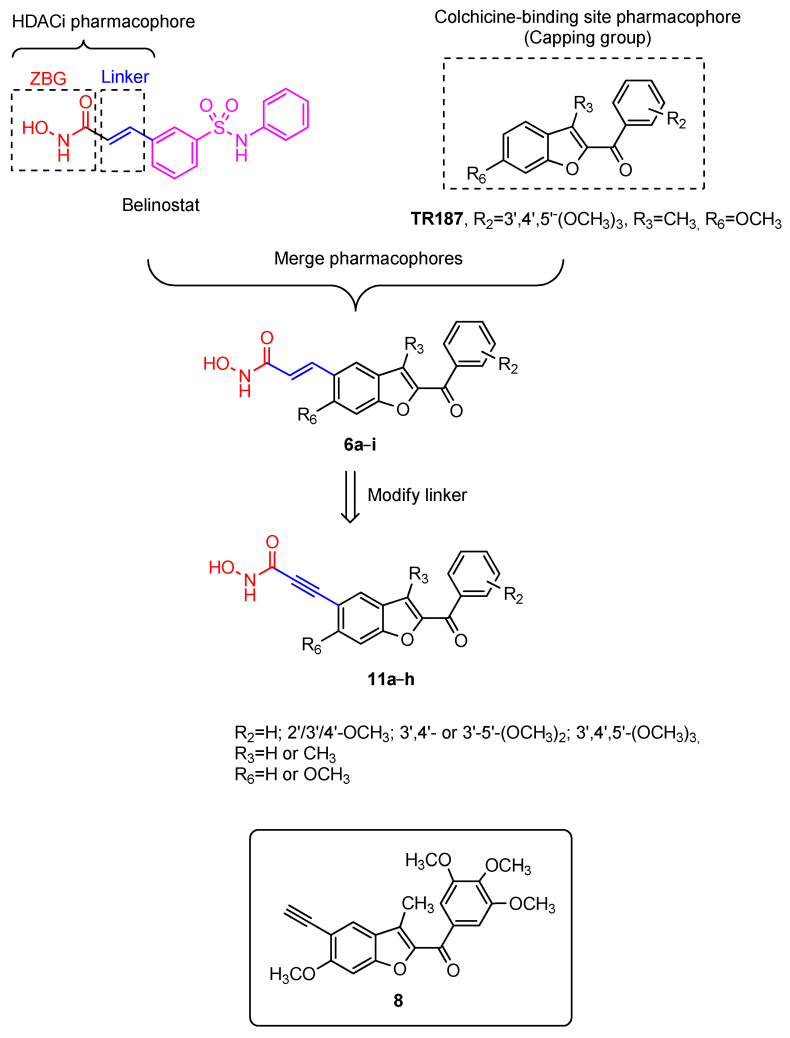
Design strategy for potential HDAC–tubulin dual inhibitors.

In a previous study, we reported the discovery of a series of methoxy-substituted 2-(3′,4′,5′-trimethoxybenzoyl) benzo[*b*]furan derivatives targeting the colchicine site of tubulin (Figure 2) [63]. The concomitant presence of a methyl and a methoxy group at the C-3 and C-6 position, respectively, of the benzo[*b*]furan ring furnished the most active compound **TR187**, which produced a significant inhibition of tubulin polymerization as well as cancer cell growth at nanomolar concentrations. To demonstrate the validity of the dual targeting strategy, compound **TR187** was selected as the lead compound for the design of novel tubulin–HDAC dual inhibitors. Based on the structure–activity relationship (SAR) found in previous studies [64,65], the C-5 position on the benzene portion of the benzo[*b*]furan ring of compound **TR187** could tolerate structural modifications. The preparation of compound **8** (Figure 2) revealed that the insertion of an acetylenic moiety (CCH) at the 5-position of compound **TR187** still retained its antiproliferative activity with IC_50_ values ranging from 18 to 207 nM against a panel of five different cancer cell lines (see Table 1). Since the 5-position of compound **TR187** would likely tolerate structural modifications, we therefore introduced in this position the critical components of the HDAC pharmacophore, the ZBG and the linker, while hoping to retain tubulin polymerization inhibitory activity. Due to its high affinity for the zinc ion, hydroxamic acid is the most frequently used ZBG in the development of HDAC inhibitors. 

Therefore, based on these findings, two new series of potential dual tubulin–HDAC inhibitors were rationally designed following a pharmacophore fusion strategy in which the 5-position of the 2-aroyl benzo[*b*]furan scaffold (as SRC group) was attached to a hydroxamic acid as ZBG via a rigid two-atom length olefinic or acetylenic linker, to generate compounds **6a**–**i** and **11a**–**h**, respectively. 

For the first series of designed derivatives **6a**–**i**, the *N*-hydroxyacrylamide group, a privileged structure found in many potent HDAC inhibitors, including the approved drugs panobinostat and belinostat, was introduced at the 5-position of the 2-aroyl benzo[*b*]furan moiety as the SRC group. 

In the second series of compounds, the vinyl linkage of compounds **6a**–**i** was replaced by an ethyne spacer, to furnish the corresponding *N*-hydroxypropiolamide derivatives **11a**–**h**. Through the synthesis of compounds **6b**–**g** and **11b**–**g**, we investigated the importance of the 3′,4′,5′-trimethoxybenzoyl group at the 2-position of the benzo[*b*]furan derivatives **6a** and **11a**, respectively, on antiproliferative activity by varying the number and position of methoxy groups on the benzoyl moiety. By the synthesis of derivatives **6h** and **11h**, we determined if the presence of the 3′,4′,5′-trimethoxyphenyl group of the 2-benzoyl moiety combined with the methoxy group at the 6-position of the benzo[*b*]furan was essential for optimal activity. 

Even though several tubulin–HDAC dual inhibitors have been reported with good results in recent years [54,66,67,68], to our knowledge, this is the first attempt to obtain a dual inhibitor based on the benzo[*b*]furan moiety.

## 2. Results 

### 2.1. Chemistry

**Scheme 1:**
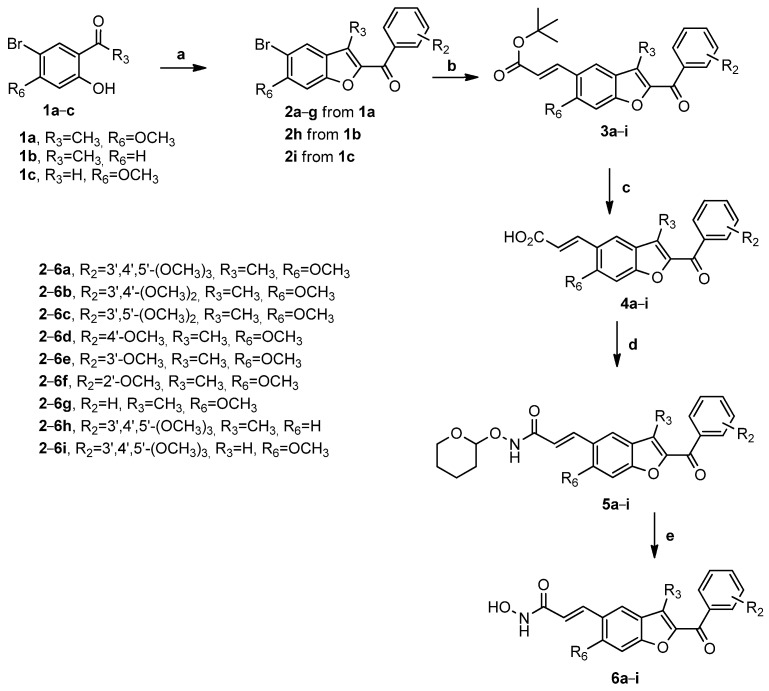
Reagents. **a**: appropriate substituted α-bromo acetophenone, K_2_CO_3_, acetonitrile, 78 °C; **b**: *tert*-butyl acrylate, Pd(OAc)_2_, PPh_3_, K_2_CO_3_, Et_3_N, DMF, 80 °C; **c**: TFA, rt; **d**: NH_2_OTHP, EDCI, HOBt, DMF, DIPEA, rt; and **e**: 4 M HCl in dioxane, rt.

The *N*-hydroxyacrylamide benzo[*b*]furan derivatives **6a**–**i** were prepared according to the procedure described in Scheme 1. The 2-aroyl-3-methyl-5-bromobenzo[b]furan derivatives **2a**–**g** and **2h** were synthesized in good yield via a “one-step” cyclization reaction of the corresponding 1-(5-bromo-2-hydroxy-4-methoxyphenyl)ethanone **1a** or 1-(5-bromo-2-hydroxyphenyl)ethanone **1b** with variously substituted α-bromo acetophenones and anhydrous potassium carbonate (K_2_CO_3_) in refluxing acetonitrile. Following the same procedure, the 2-aroyl-5-bromobenzo[*b*]furan derivative **2i** was obtained via the condensation of commercially available 4-methoxy-5-bromo salicylaldehyde **1c** with 2-bromo-1-(3,4,5-trimethoxyphenyl)ethanone. Derivatives **2a**–**i** were subjected to a Heck olefination reaction with *tert*-butyl acrylate, Palladium(II) acetate [Pd(OAc)_2_], triphenylphosphine (PPh_3_), triethylamine (Et_3_N), and potassium carbonate in dimethylformamide (DMF) at 80 °C to afford the corresponding *tert*-butyl acrylates **3a**–**i**. For these latter Heck-coupled intermediates, the conversion of the acrylic *tert*-butyl esters into the corresponding acrylic acids was achieved with trifluoroacetic acid (TFA) to afford acrylic acids **4a**–**i**. The resulting products were coupled with *O*-(tetrahydro-2*H*-pyran-2-yl)hydroxylamine (NH_2_OTHP) using N-hydroxybenzotriazole (HOBt) and 1-[3-(dimethyamino)-propyl]-3-ethylcarbodiimide hydrochloride (EDCI) in DMF at room temperature to yield the *O*-THP-protected hydroxamic acid derivatives **5a**–**i**, and the subsequent acidification with 4 M anhydrous hydrochloric acid in dioxane to remove the THP-protecting group furnished the final *N*-hydroxyacrylamides **6a**–**i**.

**Scheme 2:**
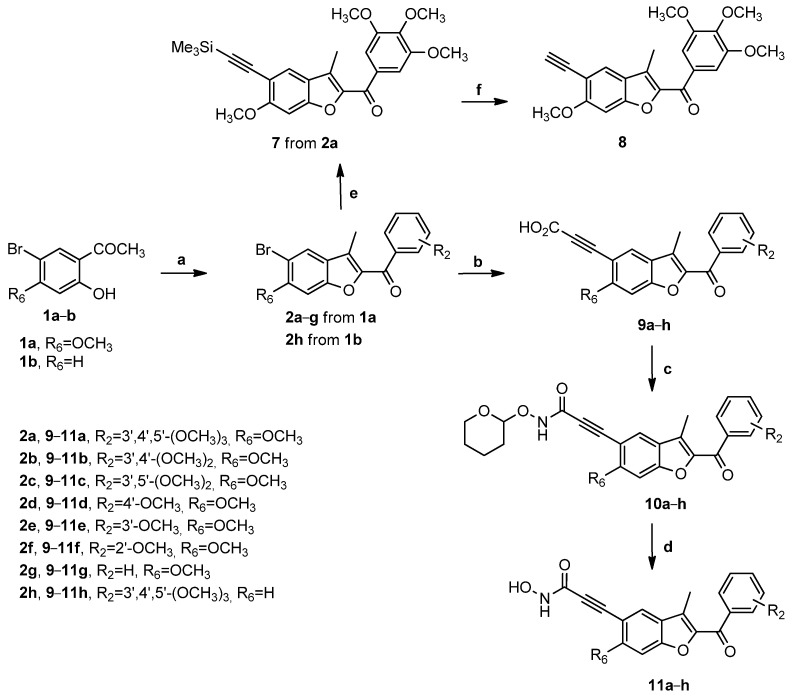
Reagents. **a**: appropriate substituted α-bromo acetophenone, K_2_CO_3_, acetonitrile, 78 °C; **b**: propynoic acid, Pd(PPh_3_)_4_, DBU, DMSO, 40 °C; **c**: NH_2_OTHP, EDCI, HOBt, Et_3_N, DMF, 50 °C; **d**: PTSA, MeOH, 50 °C; **e**: TMS-acetylene, CuI, PdCl_2_(PPh_3_)_2_, Et_3_N, THF, 100 °C; and **f**: TBAF, THF.

The synthetic procedure for the preparation of *N*-hydroxypropiolamide derivatives **11a**–**h** was described in Scheme 2. The 2-aroyl-3-methyl-5-bromobenzo[*b*]furan derivatives **2a**–**h** were further condensed with propynoic acid via a Sonogashira coupling reaction catalyzed with palladium (0) tetrakistriphenylphosphine [Pd (PPh_3_)_4_] in dimethyl sulfoxide (DMSO) at 40 °C with 1,8-diazabicyclo[5.4.0]undec-7-ene (DBU) as the base to obtain the novel propynoic acid derivatives **9a**–**h**. Using the same sequential reactions employed in the preceding Scheme 1, these latter intermediates were reacted with *O*-THP-protected hydroxylamine as NH_2_OTHP using EDC and HOBt as coupling reagents and *N*,*N*-diisopropiletilammina (DIPEA) as the base, to yield the amides **10a**–**h**. The target compounds **11a**–**h** were obtained after cleavage of the tetrahydropyran (THP) functionality mediated by *para*-toluene sulfonic acid (PTSA) in methanol (MeOH). 

For the preparation of compound **8**, the 5-bromo benzo[*b*]furan derivative **2a** was subjected to Sonogashira’s coupling reaction with trimethylsilylacetylene (TMS-acetylene) using a palladium catalyst in the presence of copper (I) iodide (CuI), bis(triphenylphosphine)palladium(II) dichloride [PdCl_2_(PPh_3_)_2_], and TEA in tetrahydrofuran (THF) to furnish the analogue **7**, followed by the removal of the trimethylsilyl-protecting group using tetrabutylammonium fluoride (TBAF) in THF, to afford alkyne derivative **8**.

### 2.2. Biological Activity and Molecular Docking Studies

#### 2.2.1. In Vitro Antiproliferative Activities 

The synthesized 2-aroyl-5-*N*-hydroxyacrylamide benzo[*b*]furan derivatives **6a**–**i** and the related *N*-hydoxypropiolamide analogues **11a**–**h** were evaluated for their ability to inhibit the growth of a panel of five human cell lines derived from different cancer types and were compared with the previously published 2-(3′,4′,5′-trimethoxybenzoyl)-3-methyl-6-methoxy benzofuran derivative **TR187** and the reference compound CA-4 (Table 1). CA-4 had nanomolar activity against the HeLa and MDA-MB-231 cancer cell lines, while A549, HT-29, and MCF-7 cells were more resistant to CA-4, with IC_50_ values of 180, 3100, and 370 nM, respectively. The 2-(3′,4′,5′-trimethoxybenzoyl) 7-*N*-hydroxyacrylamide benzo[*b*]furan derivative **6a** and the corresponding *N*-hydoxypropiolamide cognate **11a** were significantly more active than the rest of the derivatives, with IC_50_ values of 5–23 and 0.4–19 nM, respectively, superior to those obtained with compound **TR187** (IC_50_: 3–72 nM) in the five cell lines. The activity of **6a** was similar to that observed with CA-4 against HeLa and MDA-MB-231 cells, and it was the only compound of both these series equipotent to CA-4 against MDA-MB-231 cells. Derivative **11a** was 10-fold more potent than CA-4 against HeLa cells, while it was 4-fold less active than CA-4 against MDA-MB-231 cells. Along with compounds **6a** and **11a**, derivatives **6c**, **6e**, **6g**, and **11c** showed excellent antiproliferative activity against the CA-4 resistant A549, HT-29, and MCF-7 cells, with IC_50_ values ranging from single- to double-digit nanomolar concentrations.

**Table 1:** In vitro growth inhibitory activity of compounds **6a**–**i**, **8**, **11a**–**h**, **TR187**, and CA-4.

Compound	IC_50_ (nM) ^a^				
	HeLa	MDA-MB-231	A549	HT-29	MCF-7
**6a**	5.2 ± 0.03	5.0 ± 0.04	23.5 ± 0.24	7.5 ± 0.11	7.0 ± 0.2
**6b**	36.8 ± 4.13	72.6 ± 5.03	187.0 ± 1.02	401 ± 2.03	40.0 ± 4.0
**6c**	4.0 ± 0.01	26.9 ± 1.74	43.9 ± 0.55	29.0 ± 0.01	19.0 ± 1
**6d**	47.6 ± 1.22	322 ± 12.40	222.0 ± 1.09	881.0 ± 8.23	59.0 ± 10
**6e**	6.0 ± 0.05	44.7 ± 3.12	54.2 ± 2.03	7.9 ± 0.02	33 ± 6
**6f**	3.9 ± 0.01	41.9 ± 2.33	168.0 ± 5.31	75.3 ± 3.09	24 ± 8
**6g**	0.36 ± 0.01	23.9 ± 0.77	36.7 ± 0.91	80.3 ± 1.31	30 ± 0.7
**6h**	>10,000	>10,000	>10,000	>10,000	>10,000
**6i**	14.8 ± 0.32	93.7 ± 2.04	304.0 ± 1.46	272 ± 2.03	69 ± 6
**11a**	0.4 ± 0.04	16.1 ± 0.01	18.7 ± 0.72	6.6 ± 0.96	9.5 ± 0.7
**11b**	69.4 ± 1.45	377 ± 1.09	972 ± 7.47	2.16 ± 0.86	>10,000
**11c**	7.3 ± 0.03	43.9 ± 3.00	45.3 ± 2.93	96.5 ± 2.45	20 ± 0.7
**11d**	1.43 ± 0.67	4.09 ± 0.06	5.07 ± 0.22	>10,000	n.d.
**11e**	8.4 ± 0.98	96.7 ± 1.65	94.4 ± 3.03	4.89 ± 0.03	15 ± 0
**11f**	60.5 ± 2.26	345 ± 4.03	276.0 ± 6.74	4568 ± 21.45	240 ± 10
**11g**	15.2 ± 0.02	101.0 ± 0.03	206.0 ± 2.92	1.58 ± 0.06	n.d.
**11h**	>10,000	>10,000	>10,000	>10,000	>10,000
**8**	18.2 ± 0.02	81.2 ± 6.0	122 ± 8.6	207 ± 14	n.d.
**TR187**	8.5 ± 0.11	3.1 ± 0.01	24.0± 0.44	35.2 ± 0.82	72.0 ± 4
CA-4	4.6 ± 0.1	4.5 ± 2.1	180 ± 50	3100 ± 100	370 ± 100

^a^ IC_50_ = compound concentration required to inhibit tumor cell proliferation by 50%. Data are expressed as the mean ± SE from the dose–response curves of at least three independent experiments. n.d. not determined.

For compounds **6a** and **11a**, removing the C-6 methoxy group produced inactive compounds (derivatives **6h** and **11h**, respectively) with IC_50_ values greater than 10 μM, demonstrating that the presence at the 6-position of the 2-(3′,4′,5′-trimethoxybenzoyl)benzo[*b*]furan molecular skeleton of the methoxy group was essential for potent antiproliferative activity. The methyl at the C-3 position of the benzofuran nucleus also influenced antiproliferative activity. With compound **6a**, the effect of this substituent was a 2–38-fold increased potency in comparison with its parent C-3 unsubstituted counterpart **6i**. In general, the human cervix carcinoma HeLa cells were more sensitive to both these two series of compounds as compared with the other four cell lines, with derivatives **6g** and **11a** being 10-fold more potent than CA-4, while compounds **6a**, **6c,** and **6f** had nearly equivalent activity to CA-4. 

With the exception of derivative **11a**, the antiproliferative activities of all compounds with the *N*-hydroxypropiolamide moiety were reduced against the HT-29 cells as compared with the other cell lines, while HeLa and MCF-7 cells were more sensitive to this class of molecules. Comparing compounds that shared a common benzoyl moiety at the 2-position of the 6-methoxy benzo[*b*]furan skeleton, the *N*-hydroxypropiolamide derivatives were in general less potent than their *N*-hydroxyacrylamide congeners (i.e., **11a** vs. **6a**, **11b** vs. **6b**, **11c** vs. **6c**, **11d** vs. **6d**, **11e** vs. **6e**, **11f** vs. **6f**, and **11g** vs. **6g**). 

The results shown in Table 1 showed that for both series of compounds, the number and position of methoxy substituents in the phenyl portion of the benzoyl moiety at the C-2 position of the benzofuran ring influenced antiproliferative activity and selectivity against the different cancer cell lines. 

Comparing compound **6a** with the corresponding 3′, 5′-dimethoxybenzoyl derivative **6c**, these two derivatives were equipotent against HeLa cells, while a moderate two-fivefold reduction in activity with respect to compound **6a** was observed against the other four cancer cell lines. The reduction in potency was more dramatic replacing the 3′,4′,5′-trimethoxy benzoyl function of compound **6a** with the 3′, 4′-dimethoxybenzoyl moiety (compound **6b**)**.** This latter compound was 5–53-fold less active than **6a**. The reduced activity of **6b** as compared with **6a** was more pronounced against MDA-MB-231 and HT-29 cells (14- and 53-fold, respectively). A similar effect was observed in the series of *N*-hydroxypropiolamide derivatives **11a**–**c**, with the cytotoxicity being increased based on the number and position of methoxy groups, with the order being 3′,4′,5′ (**11a**) > 3′,5′ (**11c**) >> 3′,4′ (**11b**). 

In comparing both series of compounds with a methoxy group at each of the three possible positions on the phenyl of the 2-benzoyl moiety, the greatest activity occurred when the methoxy group was located at the C-2′ (**6f** and **11f**) or C-3′ (**6e** and **11e**) position, the least when located at the C-4′ position (**6d** and **11d**). 

Comparing the 5-*N*-hydroxyacrylamide benzo[*b*]furan derivatives **6d**, **6e**, and **6f**, characterized by the presence of a single methoxy group on the 2-benzoyl moiety, the 3′-methoxy and 2′-methoxy benzoyl analogues **6e** and **6f**, respectively, were equipotent against MDA-MB-231 and MCF-7 cells, with **6f** being three- and ninefold more active than **6e** against A549 and HT-29 cells, respectively. An opposite effect was observed on HeLa cells, with derivative **6f** being twofold more active than **6e**. The data shown in Table 1 also suggest that a methoxy substitution located at the 3′- and 2′-position makes compounds **6e** and **6f**, respectively, more potent than the 4′-methoxy isomer **6d** against all five cancer cell lines. In the series of *N*-hydroxypropiolamide derivatives **11d**–**f**, a comparison of mono-methoxy substituent effects with **11f** and **11e**, respectively, exceeding that of its *para*-methoxy counterpart **11d** by two- orders of magnitude, with the order of activity being 3′-OMe (**11e**) > 2′-OMe (**11f**) >> 4′-OMe (**11d**). In comparing the 2′-OMe and 3′-OMe derivatives **11f** and **11e**, respectively, the greatest activity occurred with the 3′-OMe, which was from 3- to 16-fold more potent than the 2′-OMe against four of the five cancer cell lines, while the two compounds were equipotent against HT-29 cells. Moving the methoxy group from the 3′- to the 4′-position on the benzoyl moiety (compounds **11e** and **11d**, respectively) was deleterious for antiproliferative activity, with IC_50_ values at high micromolar levels, ranging from 1.5 to 10 µM across the five cell lines. 

Comparing the 3′,5′-dimethoxybenzoyl derivative **6c** with the unsubstituted benzoyl derivative **6g**, removing both the methoxy groups on the 2-benzoyl moiety produced a 10-fold increase in activity against HeLa cells, while **6c** and **6g** were equipotent against the MDA-MB-231 and A549 cells. A slight reduction in potency (twofold) was observed only against HT-29 and MCF-7 cells. In contrast, in the series of *N*-hydroxypropiolamide derivatives, the replacement of the 3′,5′-dimethoxybenzoyl moiety of derivative **11c** by an unsubstituted benzoyl function in the compound **11g** caused a reduction in activity (2–16-fold) in all cell lines, which was more pronounced for the HT-29 cells. 

The activity of compound **6g** based on the 2-benzoyl unsubstituted moiety was superior to that of the corresponding 3′,4′-dimethoxy (**6b**) and monomethoxy (**11d**–**f**) benzoyl derivatives. Moreover, only against HeLa cancer cells compound **6g** was 14-fold more active than the 3′,4′,5′-trimethoxybenzoyl derivative **6a**, while this latter compound was 1.5–11-fold less potent than **6g** as compared with the other cell lines.

#### 2.2.2. In Vitro Inhibition of Tubulin Polymerization and Colchicine Binding

In order to understand whether these molecules were exerting their activities, at least in part, through an interaction with the colchicine site of tubulin, we compared the inhibitory effects on tubulin polymerization and on the binding of [^3^H]colchicine to tubulin of the most promising antiproliferative compounds (**6a**–**f**, **6i**, **11a**–**c**, and **11e**–**f**). CA-4 and derivative **TR187** were used as positive controls in contemporaneous experiments (Table 2). CA-4 and **TR 187** inhibited tubulin polymerization with half-maximal inhibitory concentrations of 0.91 μM and 0.62 μM, respectively. 

In the in vitro tubulin polymerization assay, the results showed that all tested compounds inhibited tubulin assembly, with compounds **6c** and **11e** being the most active with IC_50_ values of 0.42 μM (Table 2), more than twice as potent as CA-4 (IC_50_: 0.91 μM). Compounds **6a**, **6g**, **11a**, and **11c** were also more potent than CA-4 in inhibiting tubulin assembly, while several derivatives (**6b**, **6d**–**f**, and **6i**) showed antitubulin activity essentially equivalent to that of CA-4. Only compounds **11b** and **11f** were less active as inhibitors of tubulin polymerization, with IC_50_ values of 2.7 μM and 5.4 μM (three and sixfold less potent than CA-4, respectively), which is consistent with their low antiproliferative potency. All these data demonstrated that the antiproliferative activity of all tested molecules was related to inhibition of tubulin polymerization.

When comparing inhibition of tubulin polymerization with the growth inhibitory effects, we found a good correlation for most, but not all, of the active compounds. While **11e** was generally less potent than **6c** as an antiproliferative agent, the two compounds were equipotent as inhibitors of tubulin assembly. In addition, although several compounds, such as **6b**, **6g**, **11c**, and **11e** showed lower antiproliferative activity on MDA-MB-231 cancer cells when compared with CA-4, they were comparable to or more potent than CA-4 as inhibitors of tubulin assembly.

In the [^3^H]colchicine binding studies, derivatives **6c**, **6e**–**g**, **11c**, and **11e** had quantitatively similar effects, varying within a narrow range (76–86%) of inhibition, and they were slightly less potent than CA-4, which, in these experiments, inhibited colchicine binding by 98%. Thus, these compounds significantly inhibited the binding of [^3^H]colchicine to tubulin at 5 μM, suggesting that they bound to tubulin at a site overlapping the colchicine site to inhibit tubulin polymerization. Inhibition of colchicine binding by compounds **11b**, **11f**, **6b**, **6d**, and **6i** was lower, varying within the 27–43% range. 

For the most active compounds, **6a**, **6c**, **6g**, **11c**, and **11e**, a good correlation was observed between antiproliferative activities, inhibition of tubulin polymerization, and inhibition of colchicine binding. The correlation between these three assays was imperfect for compounds **6b** and **6e**. Thus, while these two molecules were equipotent in the tubulin assembly assay, compound **6e** was twofold more potent than **6b** as an inhibitor of colchicine binding. In comparison with **6a**, compound **6i** was almost threefold less potent as an inhibitor of tubulin assembly and 1.5-fold less active as an inhibitor of [^3^H]colchicine binding, suggesting that the presence of the methyl group at the 3-position of benzo[*b*]furan ring was favorable for an increased inhibition of the tubulin polymerization assembly and the binding of [^3^H]colchicine to tubulin.

It is interesting to note that for most of the tested compounds (derivatives **6c**, **6e–g**, **11c**, and **11e**), the trimethoxyphenyl moiety, a well-defined pharmacophore for the inhibition of tubulin polymerization found in colchicine, CA-4 and podophyllotoxin [69], was not essential for potent inhibition of tubulin polymerization.

#### 2.2.3. In Vitro HDAC Inhibitory Activity

To assess the HDAC enzyme inhibitory activity of the synthesized compounds with potent tubulin polymerization inhibitory activity, a commercial kit that exploits all HDACs contained in a nuclear extract from HeLa cells was used. Trichostatin (TSA), a pan-HDAC inhibitor, was used as reference compound.

**Figure 3:**
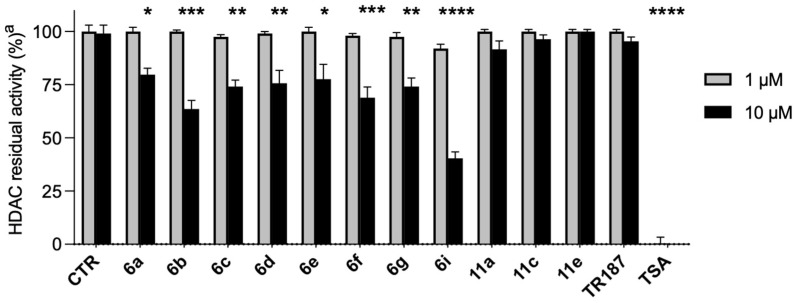
^a^ Percentage of HDAC residual activity after treatment with compounds **6a**–**g**, **6i**, **11a**, **11c**, **11e**, **TR187**, and TSA). The inhibition rate of each compound was determined at the indicated concentration using the Fluor De Lys assay^®^, and the data were expressed as the mean of two independent experiments. * *p* < 0.05; ** *p* < 0.01; *** *p* < 0.001; **** *p* < 0.0001 vs. control.

As can be seen from Figure 3, the compounds were tested at two different concentrations, 1 µM and 10 µM. The compounds, except TSA, at 1 µM showed negligible activity. However, at 10 µM, **6b**, **6f**, **6g**, and **6i** showed moderate ability to inhibit enzymatic activity (40–75% residual activity).

**Figure 4:**
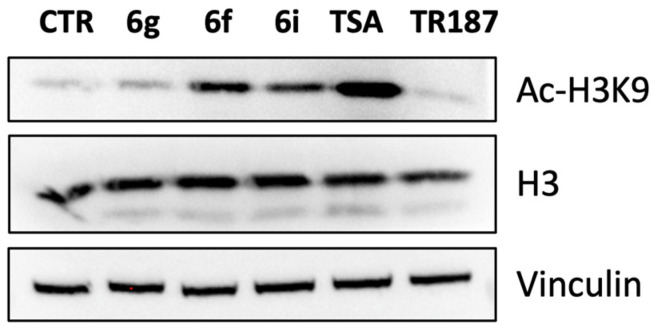
Western blot analysis of histone H3 and its acetylated form (Ac-H3K9) after treatment of HT-29 cells with the indicated compound at 10 µM for 6 h. To confirm equal protein loading an anti-vinculin antibody was used.

With the aim of verifying the potential ability of these compounds to inhibit HDAC, HT-29 cells were treated with some of the best compounds (see Figure 4) for 6 h and analyzed using Western blot. As can be seen from Figure 4, compounds **6f** and **6i** significantly increased acetylated histone H3 (Ac-H3K9), confirming the data shown in Figure 3 and demonstrating their potential as effective HDAC inhibitors in vitro. Of note, the reference antitubulin compound **TR187**, as observed in the fluorometric assay, does not show any inhibitory activity towards HDAC even at the cellular level, indicating that fusion with hydroxamic acid via a rigid two-atom length olefinic bond may be required to obtain compounds endowed with dual inhibitory activity.

To further explore the ability of the synthesized compounds to inhibit certain HDAC isoforms, some compounds were evaluated for their capacity to inhibit HDAC6 and isoforms 1, 8, and 10. In these experiments, panobinostat, a non-selective HDAC inhibitor, served as the reference compound (Table 3). Among the newly tested compounds, all displayed limited ability to inhibit HDAC6, with compounds **6a**, **6f**, and **6i** exhibiting the most notable activity, with IC_50_ values in the low micromolar range. The remaining compounds had inhibition values exceeding 10 µM. Regarding HDAC1 and 10 inhibition, only compound **6a** demonstrated IC_50_ values below 10 µM, indicating moderate potency against these isoforms. For HDAC8, none of the tested compounds exhibited any discernible activity. These results indicated that the antiproliferative activity of both series of hydroxamic acid derivatives mainly resulted from inhibition of tubulin polymerization rather than from inhibition of HDACs.

#### 2.2.4. Molecular Modeling Studies

**Figure 5:**
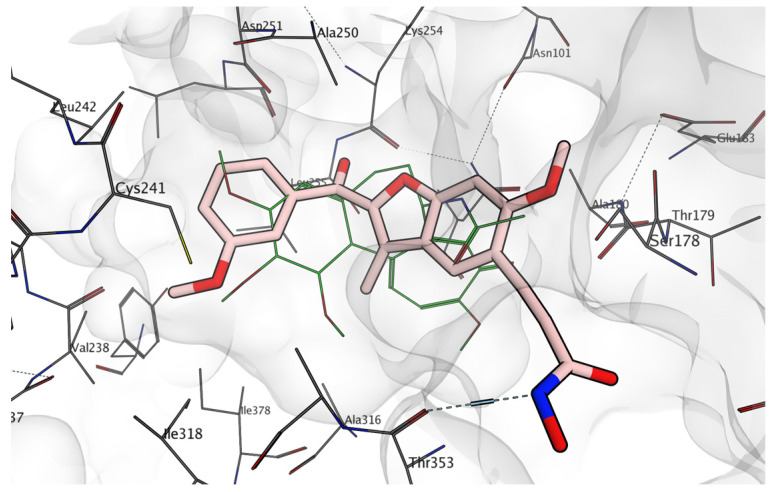
Docking results of compound **11e** (carbon atoms represented in pink) in the tubulin binding site. Co-crystallized colchicine is represented in green lines; nitrogen atoms are represented in blue; oxygen atoms are represented in red, protein carbon atoms are represented in grey.

**Figure 6:**
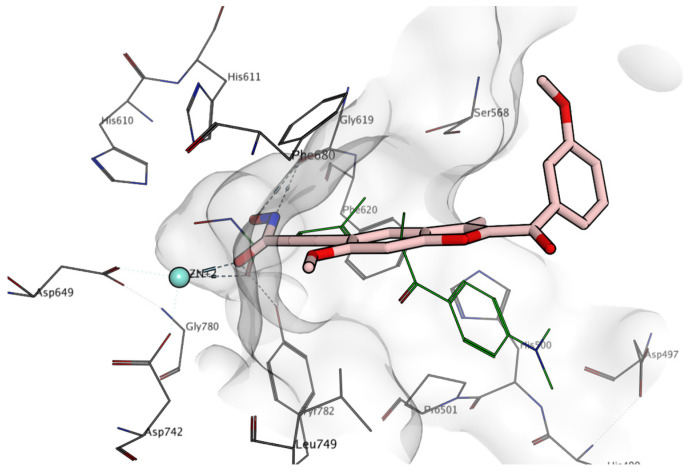
Docking results of compound **11e** (carbon atoms are depicted in pink) in the HDAC6 binding site. Co-crystallized trichostatin A is represented in green; zinc ion is represented in cyan; nitrogen atoms are represented in blue; oxygen atoms are represented in red, protein carbon atoms are represented in grey.

**Figure 7:**
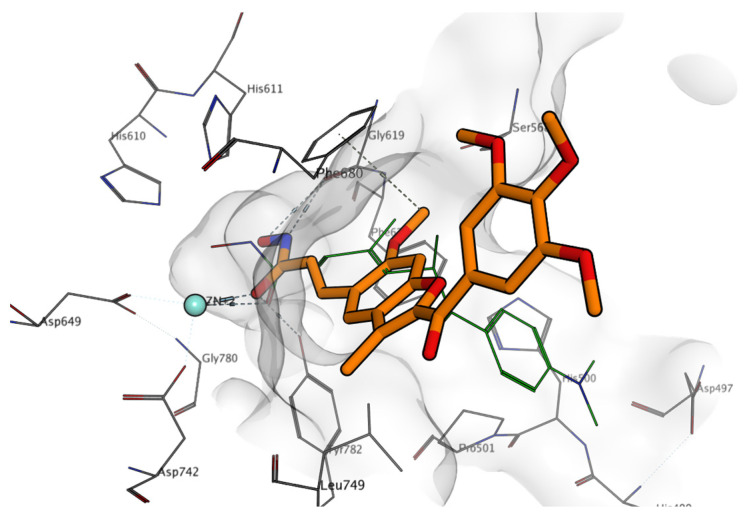
Docking results of compound **6a** (carbon atoms are depicted in orange) in the *h*HDAC6 binding site. Co-crystallized trichostatin A is represented in green lines; zinc ion is represented in cyan; nitrogen atoms are represented in blue; oxygen atoms are represented in red, protein carbon atoms are represented in grey.

To investigate further the differences in activity observed for the compounds with the two targets, we performed a series of molecular docking simulations on both tubulin and hDAC6 (Figure 5, Figure 6 and Figure 7). As shown in Figure 5, the tubulin binding site can accommodate compound **11e** well, with the methoxy-substituted phenyl ring in contact with βCys241, an interaction observed with the majority of the reported inhibitors that bind to the colchicine site [69]. Interestingly, the 3-methoxy group is in direct contact with βCys241, and this supports the experimental observation that, for these compounds, this single substitution is sufficient to engage with this key subpocket. The hydroxyamide group is facing towards the external part of the pocket, forming a hydrogen bond with βThr353. This putative binding mode supports the experimental results observed in the tubulin polymerization assay (Table 2). It should be noted that this orientation is also observed with the other compounds reported, including the hydroxyacrylamide analogues **6a**–**i** (e.g., **6a**, Figure 7). 

Compound **11e**, on the other hand, does not show any activity at the highest concentration tested against HDAC6 (Table 3). The docking results in this case show that the chelating moiety can potentially reach the Zn^2+^ ion deep in the pocket (Figure 6). However, the rigidity of the compound forces the aromatic rings of **11e** to be placed outside the pocket, losing contact also with the rim of the binding site, and remaining exposed to the solvent. This would inevitably represent a penalty in the binding affinity of the compound. Similar docking results were obtained for the other compounds described here. 

Interestingly, for the hydroxyacrylamide analogues **6a**–i, because of the different geometry of the side chain, the benzofuran ring can be placed closer to the protein. For example, in the case of compound **6a** (Figure 7), this orientation allows also for a contact between the methyl substituent with Leu749, rationalizing the modest activity observed for this compound against hHDAC6. 

#### 2.2.5. Compounds **6f**, **6g**, and **6i** Induced G2/M Arrest of the Cell Cycle

**Figure 8:**
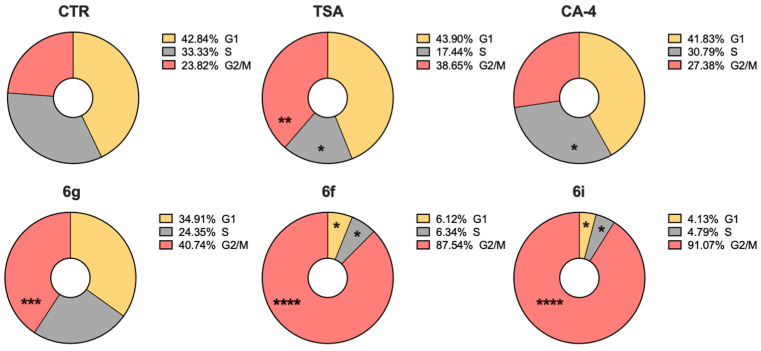
Percentage of HT-29 cells in each phase of the cell cycle treated with **6g**, **6f**, **6i**, and the two reference compounds TSA and CA-4 at 250 nM for 24 h. Cells were fixed and labeled with PI and analyzed using flow cytometry as described in the experimental section. Data represent the mean fraction of cells (%) of three independent experiments. Two-way ANOVA statistical analysis with Tukey correction for multiple comparison was applied. Only significant comparisons between treatment and CTR are shown. * *p* < 0.05; ** *p* < 0.01; *** *p* < 0.001; **** *p* < 0.0001.

Considering that both tubulin polymerization inhibitors and HDAC inhibitors affect cell proliferation, we investigated the effects of selected compounds on the cell cycle by incubating HT-29 cells, a cell line resistant to CA-4 [70,71], with compounds **6f**, **6g**, and **6i** at 10 µM for 24 h. The results shown in Figure 8 show that compounds **6f** and **6i** induced an enormous arrest of the cell cycle in G2/M with a concomitant reduction in cells in the S and G1 phases. Although significant, the effect of **6g** was not as great. These results suggest that compounds **6f**, **6i**, and **6g** are able to arrest the cell cycle in the metaphase, even in a line resistant to CA-4, in excellent agreement with the data on inhibition of tubulin polymerization. 

#### 2.2.6. Compounds **6f**, **6g**, and **6i** Induced Apoptosis in HT-29 Cells

**Figure 9:**
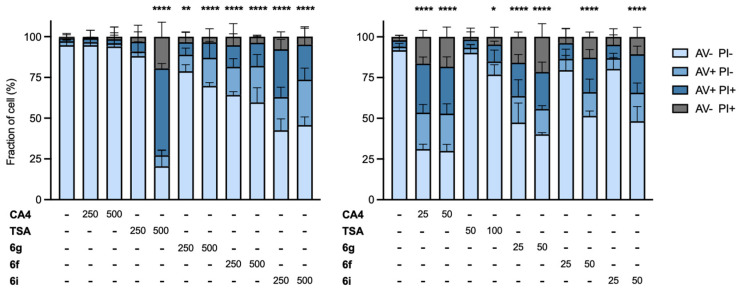
Cytofluorimetric analysis of cell death via annexin V (AV) and propidium iodide (PI) staining in HT-29 (**left panel**) and HeLa cells (**right panel**) after treatment with **6f**, **6g**, **6i**, and the reference compounds TSA and CA-4 for 96 and 48 h, respectively. Histograms represent the mean ± standard error mean (SEM) of three independent experiments. Two-way ANOVA statistical analysis with Tukey correction for multiple comparisons was applied. Only significant comparison of live cell (AV-PI-) between treatment and control are shown. * *p* < 0.05; ** *p* < 0.01; **** *p* < 0.0001.

To evaluate the ability of the selected compounds to induce apoptosis, we performed a bi-parametric cytofluorimetric assay using annexin-V-fluorescein isothiocyanate (FITC) and propidium iodide (PI), which stain phosphatidylserine (exposed only in early and late apoptotic cells) and DNA (in late apoptotic and necrotic cells), respectively. As shown in Figure 9 (left-hand panel), HT-29 cells are completely resistant to CA-4 and are sensitive only to the higher dose of TSA (500 nM). Compounds **6f**, **6g**, and **6i** were able to induce cell apoptosis in HT-29 cells, with **6i** being the most active compound but with lower cytotoxicity than TSA. 

In contrast, in the tubulin-sensitive HeLa cells, **6g** and CA4 had the greatest cytotoxic effects at the lower dose tested (25 nM). This was in agreement with the potent inhibitory effect of **6g** on tubulin polymerization.

#### 2.2.7. Evaluation of Compounds **6f**, **6g**, and **6i** in Human Peripheral Cells (PBMC)

To evaluate the potential cytotoxicity of the three best compounds towards non-tumor cells, compounds **6f**, **6g**, and **6i** were assayed in vitro against human peripheral mononuclear cells (PBMC) obtained from healthy donors. As depicted in Table 4, all compounds showed no activity in resting PBMC, while they showed a lower degree of toxicity in proliferating PBMC [phytohaemagglutinin (PHA) stimulated], having a GI_50_ of 0.51, 0.15, and 2.32 μM for **6f**, **6g**, and **6i**, respectively. 

## 3. Discussion

In this manuscript we have described the synthesis and biological evaluation of two series of synthetic antitubulin compounds based on the 2-aroyl-5-*N*-hydroxyacrylamide-6-methoxy benzo[*b*]furan skeleton and their corresponding 5-*N*-hydroxypropiolamide congeners, corresponding to compounds **6a**–**i** and **11a**–**h**, respectively. Two of the synthesized compounds, **6a** and **11a**, had the best antiproliferative activities and were more active than the rest of the derivatives. Comparing the 3-unsubstituted derivative **6i** with its 3-methyl counterpart **6**a showed that the addition of a C3 methyl group produced a 2–38-fold increase in antiproliferative activity against all five cancer cell lines.

Derivative **6a** was the only compound with activity comparable (Hela and MDA-MB-231 cells) or more potent (A549, HT-29, and MCF-7 cells) than the reference compound CA-4, with antiproliferative GI_50_ values ranging from 5 to 23 nM. For the two series of derivatives, we observed that the presence of three vicinal methoxy groups on the benzoyl moiety was not essential for activity, while the 6-methoxy substituent is important for maximal activity. Substitution of the trimethoxybenzoyl group in compounds **6a** and **11a** with 3′,5′-dimethoxybenzoyl (compounds **6c** and **11c**) or 3′-methoxybenzoyl (derivatives **6e** and **11e**) resulted in significant antiproliferative activity, demonstrating that the presence of the 3′,4′,5′-trimethoxybenzoyl moiety is optimal but not essential for activity. This finding differs from that in previous reports that indicated that the presence of a trimethoxy substituent on the 2-benzoyl moiety in the core structure of different series of benzoheterocycles, such as benzo[*b*]thiophene, benzo[*b*]furan, and indole, represent a common essential structural feature and a prerequisite for potent activity [69]. Relative to the activity of the two 2-unsubstituted benzoyl derivatives **6g** and **11g**, the insertion of a methoxy group at the *ortho*- or *meta*-positions of the benzoyl moiety was tolerated (compounds **6f**/**11f** and **6e**/**11e**, respectively), while the presence of a *para*-methoxy group (**6d** and **11d**) caused substantial loss in antiproliferative activity.

Compounds **6a**, **6c**, **6e**–**g**, **11a**, and **11c** potently inhibited tubulin polymerization, with activities higher or comparable to that of reference compound CA-4, correlating well with their antiproliferative potency against all tested cancer cell lines. The same derivatives strongly inhibited [^3^H]colchicine from binding to its site in tubulin. In particular, compound **6c** was the most potent inhibitor of tubulin polymerization and one of the most potent inhibitors of colchicine binding (IC_50_ = 0.42 μM for assembly, 81% inhibition of the binding of 5 μM colchicine, with the inhibitor and tubulin at 5 and 0.5 μM, respectively), and the antiproliferative activity of **6c**, in terms of GI_50_’s, ranged from 4 to 44 nM in the five tumor lines examined.

Although many agents in the present series have activities comparable (**6i**, **6b**, and **6d**–**f**) or superior (**6a**, **6g**, **11a**, and **11c**) to that of CA-4 as inhibitors of tubulin assembly, none were as active as CA-4 as an inhibitor of colchicine binding to tubulin. 

Results of anti-proliferative activity and tubulin and HDAC enzyme inhibitory activities were found not to be correlated because highly cytotoxic and potent inhibitors of tubulin polymerization (such as compounds **6a**–**g**, **6i**, **11a**, **11c**, and **11e**) showed relatively poor HDAC inhibitory activities against the HDAC6 isoform, with derivatives **6a**, **6f**, and **6i** exhibiting the most notable activity, with IC_50_ values in the one-digit micromolar range. Compound **6a** displayed almost comparable efficacy toward HDAC1, 6, and 10 isoforms (IC_50_ = 6.59 μM, 6.00 μM, and 4.38 μM, respectively) but significantly lower activity against HDAC8 (IC_50_ = >30 μM). All these data suggest that these compounds synthesized to contain the hydroxamic acid pharmacophore aimed at the HDACs were exerting their activities mainly by an interaction with the colchicine site of tubulin.

In cell culture experiments, compounds **6f**, **6g**, and **6i** significantly arrest the cell cycle in G2/M, as would be expected from their potent activity as inhibitors of tubulin polymerization, although, even if they have only a modest activity against HDACs, we cannot exclude a contribution from this latter activity to their antiproliferative effects. Interestingly, this activity against HDACs is also maintained in CA-4-resistant HT-29 cells. As regards the mode of cell death, the three compounds induce apoptosis even at low concentrations, in good agreement with their cytotoxic potency.

Overall, these data suggest that these new derivatives have promise as dual HDAC–tubulin inhibitors, with **6i** being the most promising compound on which to base future modeling and synthetic efforts. These should be directed at modulating the linker region and improving the fit of the SRC into the HDACs without greatly compromising affinity for the colchicine site of tubulin. 

## 4. Materials and Methods

### 4.1. Chemistry

^1^H nuclear magnetic resonance (NMR) spectroscopy was carried out using one of the following instruments: a Bruker Avance 400 or a Bruker Avance III 400 (Bruker, Milan, Italy). ^13^C NMR spectra were recorded on a Varian 400 Mercury Plus (Palo Alto, CA, USA) or a Bruker Avance III 400 spectrometer. Chemical shifts (δ) are described in ppm upfield, and the spectra were recorded in appropriate deuterated solvents, as indicated. In all cases, NMR data were consistent with the proposed structures. Characteristic chemical shifts (δ) are described in ppm using conventional abbreviations for designation of major peaks: e.g., s, singlet; d, doublet; t, triplet; q, quartet; dd, doublet of doublets; dt, doublet of triplets; and br, broad. Mass spectra were recorded with an ESI single quadrupole mass spectrometer (Waters ZQ 2000; Waters Instruments, Wilmslow, UK), and the values are expressed as [M + 1]^+^. Melting points (mp) were determined on a Buchi-Tottoli apparatus and are uncorrected. The purity of tested compounds was determined through combustion elemental analyses conducted by the Microanalytical Laboratory of the Chemistry Department of the University of Ferrara with a Yanagimoto MT-5 CHN recording elemental analyzer. All tested compounds yielded data consistent with a purity of at least 95% as compared with the theoretical values. Reaction courses and product mixtures were routinely monitored with TLC on silica gel (precoated F254 Merck plates, Merck, Darmstadt, Germany), and compounds were visualized with aqueous KMnO_4_. Flash chromatography was performed using 230–400 mesh silica gel and the indicated solvent system. Organic solutions were dried over anhydrous Na_2_SO_4_. All reagents and solvents were obtained from commercial sources and used as supplied. 

^1^H-NMR and ^13^C-NMR spectra of compounds **6a-i**, **8** and **11a-h** were provided in the supplementary materials.

#### 4.1.1. General Procedure A for the Preparation of 2-Aroyl-5-Bromo Benzo[b]furanes **2a**–**i**

A mixture of 1-(5-bromo-2-hydroxy-4-methoxyphenyl)ethanone **1a**, 1-(5-bromo-2-hydroxyphenyl)ethanone **1b**, or 5-bromo-2-hydroxy-4-methoxybenzaldehyde **1c** (1 mmol), the appropriate substituted α-bromo acetophenone (1 mmol, 1 equiv.), and potassium carbonate (138 mg, 1 mmol, 1 equiv.) in acetonitrile (5 mL) was stirred at 78 °C for 3 h. After cooling, the reaction mixture was evaporated, and the residue was portioned in a mixture of ethyl acetate (EtOAc) (10 mL) and water (5 mL). The organic layer was washed with brine, dried, and concentrated under reduced pressure to obtain a residue purified via flash chromatography.

(5-Bromo-6-methoxy-3-methylbenzofuran-2-yl)(3,4,5-trimethoxyphenyl)methanone **2a**.

Following general procedure A, the crude residue obtained from **1a** [72] and 2-bromo-1-(3,4,5-trimethoxyphenyl)ethanone [73] was purified via flash chromatography, using ethyl acetate:petroleum ether 2:8 (*v*:*v*) as eluent, to furnish **2a** as a whitish foam. Yield: 64%, mp 148–150 °C. ^1^H NMR (400 MHz, DMSO-*d*_6_) δ ppm: 2.53 (s, 3H), 3.80 (s, 3H), 3.88 (s, 6H), 3.96 (s, 3H), 7.33(s, 2H), 7.56 (s, 1H), 8.15 (s, 1H). MS (ESI): [M + 1]^+^ = 435.03, 437.07. 

(5-Bromo-6-methoxy-3-methylbenzofuran-2-yl)(3,4-dimethoxyphenyl)methanone **2b**.

Following general procedure A, the crude residue obtained from **1a** and commercially available 2-bromo-1-(3,4-dimethoxyphenyl)ethanone was purified via flash chromatography, using ethyl acetate:petroleum ether 2.5:7.5 (*v*:*v*) as eluent, to furnish **2b** as a white solid. Yield: 72%, mp 191–193 °C. ^1^H NMR (400 MHz, DMSO-*d*_6_) δ ppm: 2.50 (s, 3H), 3.83 (s, 3H), 3.86 (s, 3H), 3.93 (s, 3H), 7.11(d, J = 8.8 Hz, 1H), 7.50 (s, 1H), 7.53 (d, J = 2.0 Hz, 1H), 7.73 (dd, J = 8.8 and 2.0 Hz, 1H), 8.11 (s, 1H). MS (ESI): [M + 1]^+^ = 405.06, 407.08. 

(5-Bromo-6-methoxy-3-methylbenzofuran-2-yl)(3,5-dimethoxyphenyl)methanone **2c**. 

Following general procedure A, the crude residue obtained from **1a** and 2-bromo-1-(3,5-dimethoxyphenyl)ethanone [74] was purified via flash chromatography, using ethyl acetate:petroleum ether 1.5:8.5 (*v*:*v*) as eluent, to furnish **2c** as a white solid. Yield: 72%, mp 180–181 °C. ^1^H NMR (400 MHz, CDCl_3_) δ ppm: 2.57 (s, 3H), 3.89 (s, 6H), 3.93 (s, 3H), 6.69 (s, 1H), 7.06 (s, 1H), 7.20 (s, 2H), 7.86 (s, 1H). MS (ESI): [M + 1]^+^ = 405.14, 407.09. 

(5-Bromo-6-methoxy-3-methylbenzofuran-2-yl)(4-methoxyphenyl)methanone **2d**. 

Following general procedure A, the crude residue obtained from **1a** and commercially available 2-bromo-1-(4-methoxyphenyl)ethanone was purified via flash chromatography, using ethyl acetate:petroleum ether 1:9 (*v*:*v*) as eluent, to furnish **2d** as a white solid. Yield: 72%, mp 138–140 °C. ^1^H NMR (400 MHz, CDCl_3_) δ ppm: 2.58 (s, 3H), 3.91 (s, 3H), 3.97 (s, 3H), 6.99 (d, J = 8.8 Hz, 2H), 7.06 (s, 1H), 7.84 (s, 1H), 8.10 (d, J = 8.8 Hz, 2H). MS (ESI): [M + 1]^+^ = 375.11, 377.16. 

(5-Bromo-6-methoxy-3-methylbenzofuran-2-yl)(3-methoxyphenyl)methanone **2e**.

Following general procedure A, the crude residue obtained from **1a** and commercially available 2-bromo-1-(3-methoxyphenyl)ethanone was purified via flash chromatography, using ethyl acetate:petroleum ether 1.5:8.5 (*v*:*v*) as eluent, to furnish **2e** as a white solid. Yield: 69%, mp 106–108 °C. ^1^H NMR (400 MHz, CDCl_3_) δ ppm: 2.58 (s, 3H), 3.89 (s, 3H), 3.97 (s, 3H), 7.06 (s, 1H), 7.14–7.16 (m, 1H), 7.40 (t, J = 8.0 Hz, 1H), 7.56 (s, 1H), 7.65 (d, J = 8.0 Hz, 1H), 7.86 (s, 1H). MS (ESI): [M + 1]^+^ = 375.01, 377.02. 

(5-Bromo-6-methoxy-3-methylbenzofuran-2-yl)(2-methoxyphenyl)methanone **2f**. 

Following general procedure A, the crude residue obtained from **1a** and commercially available 2-bromo-1-(2-methoxyphenyl)ethanone was purified via flash chromatography, using ethyl acetate:petroleum ether 2:8 (*v*:*v*) as eluent, to furnish **2f** as a white solid. Yield: 83%, mp 148–150 °C. ^1^H NMR (400 MHz, CDCl_3_) δ ppm: 2.41 (s, 3H), 3.78 (s, 3H), 3.93 (s, 3H), 6.98 (s, 1H), 7.00 (d, J = 8.0 Hz, 1H), 7.06 (t, J = 8.0 Hz, 1H), 7.45 (d, J = 8.0 Hz, 1H), 7.65 (t, J = 8.0 Hz, 1H), 7.82 (s, 1H). MS (ESI): [M + 1]^+^ = 375.03, 377.05. 

(5-Bromo-6-methoxy-3-methylbenzofuran-2-yl)(phenyl)methanone **2g**. 

Following general procedure A, the crude residue obtained from **1a** and commercially available 2-bromo-1-phenylethanone was purified via flash chromatography, using ethyl acetate:petroleum ether 1:9 (*v*:*v*) as eluent, to furnish **2g** as a white solid. Yield: 64%, mp 167–168 °C. ^1^H NMR (400 MHz, CDCl_3_) δ ppm: 2.58 (s, 3H), 3.97 (s, 3H), 7.06 (s, 1H), 7.52–7.54 (m, 2H), 7.58–7.60 (m, 1H), 7.86 (s, 1H), 8.04 (d, J = 7.2 Hz, 2H). MS (ESI): [M + 1]^+^ = 345.17, 347.18. 

(5-Bromo-3-methylbenzofuran-2-yl)(3,4,5-trimethoxyphenyl)methanone **2h**. 

Following general procedure A, the crude residue obtained from commercially available **1b** and 2-bromo-1-(3,4,5-trimethoxyphenyl)ethanone was purified via flash chromatography, using ethyl acetate:petroleum ether 1.5:8.5 (*v*:*v*) as eluent, to furnish **2h** as a white solid. Yield: 75%, mp 136–138 °C. ^1^H NMR (400 MHz, CDCl_3_) δ ppm: 2.60 (s, 3H), 3.93 (s, 6H), 3.96 (s, 3H), 7.39 (s, 2H), 7.41 (d, J = 8.4 Hz, 1H), 7.57 (dd, J = 8.4 and 2.0 Hz, 1H), 7.84 (d, J = 2.0 Hz, 1H). MS (ESI): [M + 1]^+^ = 405.00, 407.27. 

5-Bromo-6-methoxybenzofuran-2-yl)(3,4,5-trimethoxyphenyl)methanone **2i**. 

Following general procedure A, the crude residue obtained from commercially available **1c** and 2-bromo-1-(3,4,5-trimethoxyphenyl)ethanone was purified via flash chromatography, using ethyl acetate:petroleum ether 3:7 (*v*:*v*) as eluent, to furnish **2i** as a whitish foam. Yield: 70%, mp 158–160 °C. ^1^H NMR (400 MHz, CDCl_3_) δ ppm: 3.95 (s, 6H), 3.96 (s, 3H), 3.99 (s, 3H), 7.14 (d, J = 0.9 Hz, 1H), 7.29 (s, 2H), 7.43 (d, J = 0.9 Hz, 1H), 7.91 (s, 1H). MS (ESI): [M + 1]^+^ = 421.2, 423.16. 

#### 4.1.2. General Procedure B for Preparing Intermediates **3a**–**i**

A mixture of compound **2a**–**i** (0.5 mmol), *tert*-butyl acrylate (0.15 mL, 1 mmol, 2 equiv.), triethylamine (0.14 mL, 1 mmol, 2 equiv.), PPh_3_ (65 mg, 0.25 mmol, 0.5 equiv.), and K_2_CO_3_ (69 mg, 0.5 mmol, 1 equiv.) in anhydrous DMF (1 mL) was repeatedly evacuated over 5 min and flushed with argon. Then, Pd(OAc)_2_ (56 mg, 0.25 mmol, 0.5 equiv.) was added, and the evacuation–flushing was repeated again. The resulting mixture was heated for 5 h at 80 °C. The reaction mixture was cooled to ambient temperature, diluted with dichloromethane (DCM), and filtered through a pad of Celite. The filtrate was concentrated in vacuo, and the resulting brown residue was purified via flash column chromatography over silica gel to afford compounds **3a**–**i**.

(*E*)-Tert-butyl 3-(6-methoxy-3-methyl-2-(3,4,5-trimethoxybenzoyl)benzofuran-5-yl)acrylate **3a**.

Following general procedure B, the crude product was purified via flash column chromatography by using petroleum ether-ethyl acetate 8-2 as eluent to yield compound **3a** as a yellow oil. Yield: 98%. ^1^H NMR (400 MHz, CDCl_3_) δ ppm: 1.55 (s, 9H), 2.61 (s, 3H), 3.94 (s, 3H), 3.95 (s, 6H), 3.96 (s, 3H), 6.52 (d, J = 16.0 Hz, 1H), 6.99 (s, 1H), 7.37 (s, 2H), 7.84 (s, 1H), 8.00 (d, J = 16.0 Hz, 1H). MS (ESI): [M + 1]^+^ = 483.39. 

(*E*)-Tert-butyl 3-(6-methoxy-3-methyl-2-(3,4-dimethoxybenzoyl)benzofuran-5-yl)acrylate **3b**. 

Following general procedure B, the crude product was purified via flash column chromatography by using petroleum ether-ethyl acetate 8-2 as eluent to yield compound **3b** as a yellow oil. Yield: 74%. ^1^H NMR (400 MHz, CDCl_3_) δ ppm: 1.55 (s, 9H), 2.61 (s, 3H), 3.95 (s, 3H), 3.97 (s, 3H), 3.98 (s, 3H), 6.49 (d, J = 16.0 Hz, 1H), 6.97 (d, J = 8.4 Hz, 1H), 7.02 (s, 1H), 7.64 (d, J = 2.0 Hz, 1H), 7.80 (s, 1H), 7.85 (d, J = 16.0 Hz, 1H), 8.04 (s, 1H). MS (ESI): [M + 1]^+^ = 453.50.

(*E*)-Tert-butyl 3-(6-methoxy-3-methyl-2-(3,5-dimethoxybenzoyl)benzofuran-5-yl)acrylate **3c**.

Following general procedure B, the crude product was purified via flash column chromatography by using petroleum ether-ethyl acetate 8.5–1.5 as eluent to yield compound **3c** as a yellow oil. Yield: 73%. ^1^H NMR (400 MHz, CDCl_3_) δ ppm: 1.55 (s, 9H), 2.60 (s, 3H), 3.86 (s, 6H), 3.94 (s, 3H), 6.46 (d, J = 16.0 Hz, 1H), 6.87 (t, J = 2.4 Hz, 1H), 7.01 (s, 1H), 7.20 (d, J = 2.4 Hz, 2H), 7.80 (s, 1H), 8.06 (d, J = 16.0 Hz, 1 H). MS (ESI): [M + 1]^+^ = 453.40. 

(*E*)-Tert-butyl 3-(6-methoxy-3-methyl-2-(4-methoxybenzoyl)benzofuran-5-yl)acrylate **3d**. 

Following general procedure B, the crude product was purified via flash column chromatography by using petroleum ether-ethyl acetate 8.5–1.5 as eluent to yield compound **3d** as a yellow solid. Yield: 80%, mp 154–156 °C. ^1^H NMR (400 MHz, CDCl_3_) δ ppm: 1.55 (s, 9H), 2.61 (s, 3H), 3.91 (s, 3H), 3.95 (s, 3H), 6.47 (d, J = 16.0 Hz, 1H), 6.99–7.02 (m, 3H), 7.75 (s, 1H), 8.02 (d, J = 16.0 Hz, 1H), 8.11 (d, J = 8.4 Hz, 2H). MS (ESI): [M + 1]^+^ = 423.32. 

(*E*)-Tert-butyl 3-(6-methoxy-3-methyl-2-(3-methoxybenzoyl)benzofuran-5-yl)acrylate **3e**. 

Following general procedure B, the crude product was purified via flash column chromatography by using petroleum ether-ethyl acetate 8.5-1.5 as eluent to yield compound **3e** as a yellow oil. Yield: 65%. ^1^H NMR (400 MHz, CDCl_3_) δ ppm: 1.55 (s, 9H), 2.60 (s, 3H), 3.88 (s, 3H), 3.94 (s, 3H), 6.47 (d, J = 16.0 Hz, 1H), 7.01 (s, 1H), 7.14–7.20 (m, 1H), 7.42 (t, J = 8.2 Hz, 1H), 7.50–7.60 (m, 1H), 7.65–7.70 (d, J = 8.2 Hz, 1H), 7.80 (s, 1H), 8.01 (d, J = 16.0 Hz, 1H). MS (ESI): [M + 1]^+^ = 423.32. 

(*E*)-Tert-butyl 3-(6-methoxy-3-methyl-2-(2-methoxybenzoyl)benzofuran-5-yl)acrylate **3f**.

Following general procedure B, the crude product was purified via flash column chromatography by using petroleum ether-ethyl acetate 9-1 as eluent to yield compound **3f** as a yellow oil. Yield: 83%. ^1^H NMR (400 MHz, CDCl_3_) δ ppm: 1.54 (s, 9H), 2.42 (s, 3H), 3.79 (s, 3H), 3.91 (s, 3H), 6.46 (d, J = 16.0 Hz, 1H), 6.94 (s, 1H), 7.01 (d, J = 7.6 Hz, 1H), 7.06 (dd, J = 7.6 and 0.8 Hz, 1H), 7.43 (dd, J = 7.6 and 1.8 Hz, 1H), 7.49–7.53 (m, 1H), 7.76 (s, 1H), 7.97 (d, J = 16.1 Hz, 1H). MS (ESI): [M + 1]^+^ = 423.30. 

(*E*)-Tert-butyl 3-(2-benzoyl-6-methoxy-3-methylbenzofuran-5-yl)acrylate **3g**. 

Following general procedure B, the crude product was purified via flash column chromatography by using petroleum ether-ethyl acetate 9-1 as eluent to yield compound **3g** as a white solid. Yield: 64%, mp 167–168 °C. ^1^H NMR (400 MHz, DMSO-*d*_6_) δ ppm: 1.55 (9H), 2.61 (s, 3H), 3.95 (s, 3H), 6.47 (d, J = 16.0 Hz, 1H), 7.01 (s, 1H), 7.50–7.53 (m, 2H), 7.60–7.64 (m, 1H), 7.80 (s, 1H), 8.00 (d, J = 16.0 Hz, 1H), 8.04 (d, J = 6.8 Hz, 2H). MS (ESI): [M + 1]^+^ = 393.33. 

(*E*)-Tert-butyl 3-(3-methyl-2-(3,4,5-trimethoxybenzoyl)benzofuran-5-yl)acrylate **3h**. 

Following general procedure B, the crude product was purified via flash column chromatography by using petroleum ether-ethyl acetate 8.5-1.5 as eluent to yield compound **3h** as a yellow oil. Yield: 61%. ^1^H NMR (400 MHz, CDCl_3_) δ ppm: 1.56 (s, 9H), 2.65 (s, 3H), 3.94 (s, 6H), 3.97 (s, 3H), 6.41 (d, J = 16.0 Hz, 1H), 7.41 (s, 2H), 7.52 (d, J = 8.2 Hz, 1H), 7.65 (dd, J = 8.2 and 2.0 Hz, 1H), 7.75 (d, J = 16.0 Hz, 1H), 7.82 (d, J = 2.0 Hz, 1H). MS (ESI): [M + 1]^+^ = 453.28. 

(*E*)-Tert-butyl 3-(6-methoxy-2-(3,4,5-trimethoxybenzoyl)benzofuran-5-yl)acrylate **3i**. 

Following general procedure B, the crude product was purified via flash column chromatography by using petroleum ether-ethyl acetate 8-2 as eluent to yield compound **3i** as a yellow oil. Yield: 91%. ^1^H NMR (400 MHz, CDCl_3_) δ ppm: 1.55 (s, 9H), 3.95 (s, 6H), 3.96 (s, 3H), 3.99 (s, 3H), 6.46 (d, J = 16.0 Hz, 1H), 7.10 (s, 1H), 7.29 (s, 2H), 7.47 (s, 1H), 7.84 (s, 1H), 7.96 (d, J = 16.0 Hz, 1H). MS (ESI): [M + 1]^+^ = 469.28. 

#### 4.1.3. General Procedure C for Preparing Intermediates **4a**–**i**

Trifluoroacetic acid (3.0 mL, 22 mmol, 44 equiv.) was added to the respective acrylic acid *tert*-butyl ester derivative **3a**–**i** (0.5 mmol), and the mixture was stirred at room temperature for 1 h. The reaction was then cooled at 0 °C and quenched with water under stirring to afford a white solid. The precipitated solid was collected via filtration, dried under vacuum on P_2_O_5_, and the acrylic acid product was used for the next reaction without further purification.

(*E*)-3-(6-Methoxy-3-methyl-2-(3,4,5-trimethoxybenzoyl)benzofuran-5-yl)acrylic acid **4a**.

Following general procedure C, compound **4a** was obtained as a pink solid. Yield: 87%, mp 218–220 °C. ^1^H NMR (400 MHz, DMSO-*d*_6_) δ ppm: 2.54 (s, 3H), 3.80 (s, 3H), 3.85 (s, 6H), 3.94 (s, 3H), 6.63 (d, J = 16.4 Hz, 1H), 7.30 (s, 2H), 7.44 (s, 1H), 7.88 (d, J = 16.4 Hz, 1H), 8.25 (s, 1H). MS (ESI): [M + 1]^+^ = 427.40. 

(*E*)-3-(2-(3,5-Dimethoxybenzoyl)-6-methoxy-3-methylbenzofuran-5-yl)acrylic acid **4b**.

Following general procedure C, compound **4b** was isolated as a red solid. Yield: >95%, mp 170–172 °C. ^1^H NMR (400 MHz, DMSO-*d*_6_) δ ppm: 2.54 (s, 3H), 3.83 (s, 3H), 3.86 (s, 3H), 3.94 (s, 3H), 6.64 (d, J = 16.4 Hz, 1H), 7.12 (d, J = 8.4 Hz, 1H), 7.40 (s, 1H), 7.53 (d, J = 2.0 Hz, 1H), 7.74 (dd, J = 8.4 and 2.0 Hz, 1H), 7.91 (d, J = 16.4 Hz, 1H), 8.23 (s, 1H). MS (ESI): [M + 1]^+^ = 395.17. 

(*E*)-3-(2-(3,5-Dimethoxybenzoyl)-6-methoxy-3-methylbenzofuran-5-yl)acrylic acid **4c**.

Following general procedure C, derivative **4c** was obtained as a red solid. Yield: 96%, mp 202–204 °C. ^1^H NMR (400 MHz, DMSO-*d*_6_) δ ppm: 2.54 (s, 3H), 3.81 (s, 6H), 3.94 (s, 3 H), 6.63 (d, J = 16.0 Hz, 1H), 6.78 (t, J = 2.4 Hz, 1 H), 7.07 (d, J = 2.4 Hz, 2H), 7.42 (s, 1H), 7.88 (d, J = 16.0 Hz, 1H), 8.25 (s, 1 H). MS (ESI): [M + 1]^+^ = 397.18. 

(*E*)-3-(6-Methoxy-2-(4-methoxybenzoyl)-3-methylbenzofuran-5-yl)acrylic acid **4d**. 

Following general procedure C, compound **4d** was isolated as a yellow solid. Yield: >95%, mp 203–205 °C. ^1^H NMR (400 MHz, DMSO-*d*_6_) δ ppm: 2.55 (s, 3H), 3.86 (s, 3H), 3.94 (s, 3H), 6.63 (d, J = 16.0 Hz, 1H), 7.10 (d, J = 8.4 Hz, 2H), 7.39 (s, 1H), 7.88 (d, J = 16.0 Hz, 1H), 8.01 (d, J = 8.4 Hz, 2H), 8.24 (s, 1H), 13.2 (bs, 1H). MS (ESI): [M + 1]^+^ = 367.18. 

(*E*)-3-(6-Methoxy-2-(3-methoxybenzoyl)-3-methylbenzofuran-5-yl)acrylic acid **4e**. 

Following general procedure C derivative **4e** was obtained as a yellow solid. Yield: >95%, mp 208–210 °C. ^1^H NMR (400 MHz, DMSO-*d*_6_) δ ppm: 2.55 (s, 3H), 3.83 (s, 3H), 3.93 (s, 3H), 6.63 (d, J = 16.4 Hz, 1H), 7.21 (dd, J = 8.2 and 2.0 Hz, 1H), 7.42 (s, 1H), 7.45–7.48 (m, 2H), 7.54 (d, J = 8.2 Hz, 1H), 7.88 (d, J = 16.4 Hz, 1H), 8.26 (s, 1H). MS (ESI): [M + 1]^+^ = 367.27. 

(*E*)-3-(6-Methoxy-2-(2-methoxybenzoyl)-3-methylbenzofuran-5-yl)acrylic acid **4f**. 

Following general procedure C, compound **4f** was obtained as a yellow solid. Yield >95%, mp 178–180 °C. ^1^H NMR (400 MHz, DMSO-*d*_6_) δ ppm: 2.39 (s, 3H), 3.73 (s, 3H), 3.92 (s, 3H), 6.64 (d, J = 16.4 Hz, 1H), 7.09 (td, J = 7.4 and 1.0 Hz, 1H), 7.20 (dd, J = 8.5 and 1.0 Hz, 1H), 7.34 (s, 1H), 7.37 (dd, J = 7.5 and 2.0 Hz, 1H), 7.55 (ddd, J = 8.4, 7.4 and 1.8 Hz, 1H), 7.90 (d, J = 16.4 Hz, 1H), 8.23 (s, 1H). MS (ESI): [M + 1]^+^ = 367.17. 

(*E*)-3-(2-Benzoyl-6-methoxy-3-methylbenzofuran-5-yl)acrylic acid **4g**. 

Following general procedure C, compound **4g** was obtained as a pink solid. Yield: 87%, mp 202–204 °C. ^1^H NMR (400 MHz, DMSO-*d*_6_) δ ppm: 2.55 (s, 3H), 3.93 (s, 3H), 6.63 (d, J = 16.4 Hz, 1H), 7.39 (s, 1H), 7.52–7.55 (m, 2H), 7.62–7.65 (m, 1H), 7.88 (J = 16.4 Hz, 1H), 7.94 (dd, J = 8.4 and 1.6 Hz, 2H), 8.26 (s, 1H). MS (ESI): [M + 1]^+^ = 337.40. 

(*E*)-3-(3-Methyl-2-(3,4,5-trimethoxybenzoyl)benzofuran-5-yl)acrylic acid **4h**. 

Following general procedure C, compound **4h** was obtained as a brown solid. Yield: 89%, mp 206–207 °C. ^1^H NMR (400 MHz, DMSO-*d*_6_) δ ppm: 2.54 (s, 3H), 3.77 (s, 3H), 3.84 (s, 6H), 6.61 (d, J = 16.0 Hz, 1H), 7.32 (s, 2H), 7.71 (d, J = 16.0 Hz, 1H), 7.76 (d, J = 2.4 Hz, 1H), 7.92 (dd, J = 8.2 and 1.6 Hz, 1H), 8.22 (d, J = 1.6 Hz, 1H). MS (ESI): [M + 1]^+^ = 397.18. 

(*E*)-3-(6-Methoxy-2-(3,4,5-trimethoxybenzoyl)benzofuran-5-yl)acrylic acid **4i**. 

Following general procedure C, compound **4i** was obtained as a yellow solid. Yield: 87%, mp 180–181 °C. ^1^H NMR (400 MHz, DMSO-*d*_6_) δ ppm: 3.76 (s, 3H), 3.86 (s, 6H), 3.94 (s, 3H), 6.52 (d, J = 16.0 Hz, 1H), 7.25 (s, 2H), 7.50 (s, 1H), 7.88 (d, J = 16.0 Hz, 1H), 8.05 (s, 1H), 8.13 (s, 1H). MS (ESI): [M + 1]^+^ = 413.40.

#### 4.1.4. General Procedure D for the Synthesis of Intermediates **5a**–**i**

To a solution of acrylic acid derivative **4a**–**i** (0.25 mmol) in DMF (2 mL), *N*,*N*-diisopropylethylamine or DIPEA (0.11 mL, 0.625 mmol, 2.5 equiv.) was added, and the reaction mixture was stirred at room temperature. After 10 min, the reaction mixture was cooled at 0 °C, HOBt (40.5 mg, 0.3 mmol, 1.2 equiv.) and EDCI (58 mg, 0.3 mmol, 1.2 equiv.) were added, and the solution was stirred for 30 min at room temperature. Subsequently, O-(tetrahydro-2H-pyran-2-yl)hydroxylamine (NH_2_OTHP) (35 mg, 0.3 mmol, 1.2 equiv) was added. After stirring for 4 h at room temperature, the reaction was quenched with water and extracted with DCM (3 × 10 mL), and the organic layer was washed with brine, dried over Na_2_SO_4_, filtered, and evaporated under reduced pressure. The resulting residue was purified via flash column chromatography on silica gel using the appropriate mixture of ethyl acetate and petroleum ether as eluent to furnish the THP-protected hydroxamic acid derivatives **5a**–**i**.

(*E*)-3-(6-Methoxy-3-methyl-2-(3,4,5-trimethoxybenzoyl)benzofuran-5-yl)-*N*-((tetrahydro-2*H*-pyran-2-yl)oxy)acrylamide **5a**.

Following general procedure D, the crude residue was purified via flash chromatography, using ethyl acetate:petroleum ether 6:4 (*v*:*v*) as eluent, to furnish **5a** as a yellow solid. Yield: 69%, mp 93–95 °C. ^1^H NMR (400 MHz, DMSO-*d*_6_) δ ppm: 1.54 (bs, 4H), 1.68 (bs, 3H), 2.53 (s, 3H), 3.50 (d, J = 11.0 Hz, 1H), 3.77 (s, 3H), 3.85 (s, 6H), 3.95 (s, 3H), 4.95 (bs, 1H), 6.60 (d, J = 16.0 Hz, 1H), 7.30 (s, 2H), 7.45 (s, 1H), 7.80 (d, J = 16.0 Hz, 1H), 7.98 (s, 1H), 11.2 (s, 1H). MS (ESI): [M + 1]^+^ = 526.63. 

(*E*)-3-(6-Methoxy-3-methyl-2-(3,4-trimethoxybenzoyl)benzofuran-5-yl)-*N*-((tetrahydro-2*H*-pyran-2-yl)oxy)acrylamide **5b**.

Following general procedure D, the crude residue was purified via flash chromatography, using ethyl acetate:petroleum ether 6:4 (*v*:*v*) as eluent, to furnish **5b** as a yellow oil. Yield: 82%. ^1^H NMR (400 MHz, DMSO-*d*_6_) δ ppm: 1.52 (bs, 4H), 1.68 (m, 3H), 2.53 (s, 3H), 3.52 (d, J = 11.3 Hz, 1H), 3.83 (s, 3H), 3.86 (s, 3H), 3.95 (s, 3H), 4.95 (bs, 1H), 6.65 (d, J = 16.0 Hz, 1H), 7.12 (d, J = 8.4 Hz, 1H), 7.41 (s, 1H), 7.53 (d, J = 2.0 Hz, 1H), 7.71–7.81 (m, 2H), 8.00 (s, 1H), 11.18 (s, 1H). MS (ESI): [M + 1]^+^ = 496.39. 

(*E*)-3-(6-Methoxy-3-methyl-2-(3,5-trimethoxybenzoyl)benzofuran-5-yl)-*N*-((tetrahydro-2*H*-pyran-2-yl)oxy)acrylamide **5c**. 

Following general procedure D, the crude residue was purified via flash chromatography, using ethyl acetate:petroleum ether 1:1 (*v*:*v*) as eluent, to furnish **5c** as a yellowish oil. Yield: 91%. ^1^H NMR (400 MHz, CDCl_3_) δ ppm: 1.26 (bs, 2H), 1.65 (bs, 3H), 1.80 (bs, 2H), 2.60 (s, 3H), 3.62–3.65 (m, 1H), 3.86 (s, 6H), 3.95 (s, 3H), 5.00 (bs, 1H), 6.65 (t, J = 2.4 Hz, 1H), 7.02 (s, 1H), 7.20 (d, J = 2.4 Hz, 2H), 7.80 (s, 1H), 8.10 (d, J = 16.0 Hz, 1H), 8.42 (s, 1H). MS (ESI): [M + 1]^+^ = 496.48. 

(*E*)-3-(6-Methoxy-2-(4-methoxybenzoyl)-3-methylbenzofuran-5-yl)-*N*-((tetrahydro-2*H*-pyran-2-yl)oxy)acrylamide **5d**. 

Following general procedure D, the crude residue was purified via flash chromatography, using ethyl acetate:petroleum ether 6:4 (*v*:*v*) as eluent, to furnish **5d** as a yellow solid. Yield: 91%, mp 98–100 °C. ^1^H NMR (400 MHz, DMSO-*d*_6_) δ ppm: 1.54 (bs, 4H), 1.73 (bs, 2H), 2.54 (s, 3H), 3.56 (d, J = 12.8 Hz, 1H), 3.86 (m, 3H), 3.94 (s, 3H), 4.94 (bs, 1H), 6.60 (d, J = 16.0 Hz, 1H), 7.09 (d, J = 8.8 Hz, 2H), 7.39 (s, 1H), 7.80 (d, J = 16.0 Hz, 1H), 8.01 (m, 3H), 11.2 (bs, 1H). MS (ESI): [M + 1]^+^ = 464.17. 

(*E*)-3-(6-Methoxy-2-(3-methoxybenzoyl)-3-methylbenzofuran-5-yl)-*N*-((tetrahydro-2*H*-pyran-2-yl)oxy)acrylamide **5e**. 

Following general procedure D, the crude residue was purified via flash chromatography, using ethyl acetate:petroleum ether 6:4 (*v*:*v*) as eluent, to furnish **5e** as a yellow oil. Yield: 78%. ^1^H NMR (400 MHz, DMSO-*d*_6_) δ ppm: 1.50 (bs, 4H), 1.70 (bs, 2H), 2.54 (s, 3H), 3.52 (d, J = 10.2 Hz, 1H), 3.82 (s, 3H), 3.96 (s, 3H), 4.94 (bs, 1H), 6.62 (d, J = 16.0 Hz, 1H), 7.22 (ddd, J = 8.2, 2.6 and 1.2 Hz, 1H), 7.48 (dd, J = 2.6 and 1.2 Hz, 1H), 7.49–7.52 (m, 2H), 7.56–7.58 (m, 1H), 7.78 (d, J = 16.0 Hz, 1H), 8.02 (s, 1H), 11.2 (bs, 1H). MS (ESI): [M + 1]^+^ = 466.29.

(*E*)-3-(6-Methoxy-2-(2-methoxybenzoyl)-3-methylbenzofuran-5-yl)-*N*-((tetrahydro-2*H*-pyran-2-yl)oxy)acrylamide **5f**.

Following general procedure D, the crude residue was purified via flash chromatography, using ethyl acetate: petroleum ether 6:4 (*v*:*v*) as eluent, to furnish **5f** as a yellowish oil. Yield: 80%. ^1^H NMR (400 MHz, DMSO-*d*_6_) δ ppm: 1.48 (m, 4H), 1.75 (bs, 3H), 2.39 (s, 3H), 3.54 (d, J = 11.3 Hz, 1H), 3.74 (s, 3H), 3.92 (s, 3H), 5.02 (bs, 1H), 6.64 (d, J = 16.0 Hz, 1H), 7.09 (td, J = 7.4 and 1.0 Hz, 1H), 7.20 (dd, J = 8.5 and 1.0 Hz, 1H), 7.35 (s, 1H), 7.37 (dd, J = 7.6 and 1.8 Hz, 1H), 7.55 (ddd, J = 8.4, 7.4 and 1.8 Hz, 1H), 7.76 (d, J = 16.0 Hz, 1H), 7.99 (s, 1H), 11.20 (s, 1H). MS (ESI): [M + 1]^+^ = 466.21.

(*E*)-3-(2-Benzoyl-6-methoxy-3-methylbenzofuran-5-yl)-*N*-((tetrahydro-2*H*-pyran-2-yl)oxy)acrylamide **5g**.

Following general procedure D, the crude residue was purified via flash chromatography, using ethyl acetate:petroleum ether 1:1 (*v*:*v*) as eluent, to furnish **5g** as a yellow oil. Yield: 88%.^1^H NMR (400 MHz, DMSO-*d*_6_) δ ppm: 1.54 (bs, 4H), 1.66 (bs, 3H), 2.55 (s, 3H), 3.54 (d, J = 11.8 Hz, 1H), 3.98 (s, 3H), 4.95 (bs, 1H), 6.60 (d, J = 16.0 Hz, 1H), 7.40 (s, 1H), 7.55 (t, J = 7.6 Hz, 2H), 7.64 (d, J = 7.6 Hz, 1H), 7.78 (d, J = 16.0 Hz, 1H), 7.95 (d, J = 7.6 Hz, 2H), 8.03 (s, 1H), 11.2 (bs, 1H). MS (ESI): [M + 1]^+^ = 436.27. 

(*E*)-3-(3-Methyl-2-(3,4,5-trimethoxybenzoyl)benzofuran-5-yl)-*N*-((tetrahydro-2*H*-pyran-2-yl)oxy)acrylamide **5h**. 

Following general procedure D, the crude residue was purified via flash chromatography, using ethyl acetate:petroleum ether 1.5–8.5 (*v*:*v*) as eluent, to furnish **5h** as a yellow oil. Yield: 89%. ^1^H NMR (400 MHz, DMSO-*d*_6_) δ ppm: 1.44 (bs, 4H), 1.63 (bs, 3H), 2.57 (s, 3H), 3.55 (d, J = 11.4 Hz, 1H), 3.79 (s, 3H), 3.86 (s, 6H), 4.92 (bs, 1H), 6.59 (d, J = 16.0 Hz, 1H), 7.34 (s, 2H), 7.66 (d, J = 16.0 Hz, 1H), 7.72 (d, J = 8.2 Hz, 1H), 7.88 (d, J = 8.2 and 2.0 Hz, 1H), 8.10 (d, J = 2.0 Hz, 1H), 11.23 (s, 1H). MS (ESI): [M + 1]^+^ = 496.45. 

(*E*)-3-(6-Methoxy-2-(3,4,5-trimethoxybenzoyl)benzofuran-5-yl)-*N*-((tetrahydro-2*H*-pyran-2-yl)oxy)acrylamide **5i**. 

Following general procedure D, the crude residue was purified via flash chromatography, using ethyl acetate:petroleum ether 7:3 (*v*:*v*) as eluent, to furnish **5i** as a yellow oil. Yield: 52%. ^1^H NMR (400 MHz, DMSO-*d*_6_) δ ppm: 1.45 (m, 4H), 1.73 (bs, 3H), 3.49 (d, J = 10.6 Hz, 1H), 3.79 (s, 3H), 3.89 (s, 6H), 3.98 (s, 3H), 5.00 (bs, 1H), 6.59 (d, J = 16.0 Hz, 1H), 7.28 (s, 2H), 7.52 (s, 1H), 7.77 (d, J = 16.0 Hz, 1H), 7.84 (s,1H), 7.98 (s, 1H), 11.24 (s, 1H). MS (ESI): [M + 1]^+^ = 512.46.

#### 4.1.5. General Procedure E for Preparing Target Compounds **6a**–**i**

The THP protected intermediate **6a**–**i** (0.2 mmol) was dissolved in 4 M HCl in 1,4-dioxane (0.1 mmol/mL dioxane), and the mixture was stirred for 1 h at room temperature. The reaction was monitored with TLC, which indicated a complete conversion. The solvent was removed via evaporation and the residue was suspended with ethyl ether. Then, the precipitated solid was removed via filtration, washed with ethyl ether, and dried in vacuo to yield final compounds **6a**–**i**. 

(*E*)-*N*-Hydroxy-3-(6-methoxy-3-methyl-2-(3,4,5-trimethoxybenzoyl)benzofuran-5-yl)acrylamide **6a**.

Following general procedure E, the desired compound **6a** was obtained as a white solid. Yield: 83%, mp 180–182 °C. ^1^H NMR (400 MHz, DMSO-*d*_6_) δ ppm: 2.53 (s, 3H), 3.76 (s, 3H), 3.85 (s, 6H), 3.94 (s, 3H), 6.62 (d, J = 16.0 Hz, 1H), 7.30 (s, 2H), 7.43 (s, 1H), 7.74 (d, J = 16.0 Hz, 1H), 7.99 (s, 1H), 9.04 (bs, 1H), 10.7 (bs, 1H). ^13^C NMR (400 MHz, DMSO-*d*_6_) δ ppm: 10.42, 56.57 (2C), 56.91, 60.67, 95.73, 107.43 (2C), 120.11, 121.12, 122.03, 122.35, 127.49, 133.10, 133.81, 141.91, 148.14, 153.04 (2C), 156.15, 159.78, 163.58, 183.65. MS (ESI) *m*/*z* calcd. for C_23_H_23_NO_8_ [M + 1]^+^: 442.44, found: 442.40. Anal. calcd for C_23_H_23_NO_8_. C, 62.58; H, 5.25; N, 3.17; found: C, 62.62; H, 5.32; N, 3.23.

(*E*)-3-(2-(3,4-Dimethoxybenzoyl)-6-methoxy-3-methylbenzofuran-5-yl)-*N*-hydroxyacrylamide **6b**.

Following general procedure E, compound **6b** was isolated as a yellow solid. Yield: 81%, mp 198–199 °C. ^1^H NMR (400 MHz, DMSO-*d*_6_) δ ppm: 2.55 (s, 3H), 3.85 (s, 3H), 3.88 (s, 3H), 3.96 (s, 3H), 6.62 (d, J = 16.0 Hz, 1H), 7.13 (d, J = 8.4 Hz, 1H), 7.41 (s, 1H), 7.55 (d, J = 2.0 Hz, 1H), 7.73–7.80 (m, 2H), 7.99 (s, 1H). ^13^C NMR (400 MHz, DMSO-*d*_6_) δ ppm: 10.07, 55.78, 56.00, 56.62, 95.42, 111.04, 112.11, 119.83, 120.76, 121.69, 122.10, 124.42, 126.52, 130.10, 133.53, 148.24, 148.71, 153.11, 155.71, 159.33, 163.30, 182.88. MS (ESI) *m*/*z* calcd. for C_22_H_21_NO_7_ [M + 1]^+^: 412.41, found: 412.35. Anal. calcd for C_22_H_21_NO_7_. C, 64.23; H, 5.14; N, 3.40; found: C, 64.31; H, 5.22; N, 3.48. 

(*E*)-3-(2-(3,5-Dimethoxybenzoyl)-6-methoxy-3-methylbenzofuran-5-yl)-*N*-hydroxyacrylamide **6c**. 

Following general procedure E, the desired compound **6c** was isolated as a yellow solid. Yield: 62%, mp 170–172 °C. ^1^H NMR (400 MHz, DMSO-*d*_6_) δ ppm: 2.53 (s, 3H), 3.80 (s, 6H), 3.93 (s, 3H), 6.60 (d, J = 16.0 Hz, 1H), 6.78 (t, J = 2.4 Hz, 1H), 7.09 (d, J = 2.4 Hz, 2H), 7.41 (s, 1H), 7.71 (d, J = 16.0 Hz, 1H), 7.98 (s, 1H), 10.2 (bs, 1H). ^13^C NMR (400 MHz, DMSO-*d*_6_) δ ppm: 10.38, 55.97 (2C), 56.91, 95.70, 104.61, 107.41 (2C), 120.20, 121.20, 122.11, 122.29, 127.98, 133.70, 139.88, 148.00, 156.16, 159.89, 160.74 (2C), 161.51, 184.33. MS (ESI) *m*/*z* calcd. for C_22_H_21_NO_7_ [M + 1]^+^: 412.41, found: 412.30. Anal. calcd for C_22_H_21_NO_7_. C, 64.23; H, 5.14; N, 3.40; found: C, 64.35; H, 5.26; N, 3.51.

(*E*)-*N*-Hydroxy-3-(6-methoxy-2-(4-methoxybenzoyl)-3-methylbenzofuran-5-yl)acrylamide **6d**. 

Following general procedure E, compound **6d** was isolated as a yellow solid. Yield: 90%, mp 168–170 °C. ^1^H NMR (400 MHz, DMSO-*d*_6_) δ ppm: 2.56 (s, 3H), 3.88 (s, 3H), 3.95 (s, 3H), 6.63 (d, J = 16.0 Hz, 1H), 7.07–7.15 (m, 2H), 7.39 (s, 1H), 7.76 (d, J = 16.0 Hz, 1H), 7.99 (s, 1H), 8.01–8.07 (m, 2H). ^13^C NMR (400 MHz, DMSO-*d*_6_) δ ppm: 10.26, 56.02, 56.84, 95.64, 114.28 (2C), 120.10, 121.02, 121.94, 122.33, 126.85, 130.43, 132.19 (2C), 133.75, 148.46, 155.91, 159.57, 163.37, 163.52, 183.19. MS (ESI) *m*/*z* calcd. for C_21_H_19_NO_6_ [M + H]^+^: 382.38, found: 382.30. Anal. calcd for C_21_H_19_NO_6_. C, 66.13; H, 5.02; N, 3.67; found: C, 66.23; H, 5.16; N, 3.85.

(*E*)-*N*-Hydroxy-3-(6-methoxy-2-(3-methoxybenzoyl)-3-methylbenzofuran-5-yl)acrylamide **6e**. 

Following general procedure E, the desired compound **6e** was isolated as a yellowish solid. Yield: 82%, mp 100–102 °C. ^1^H NMR (400 MHz, DMSO-*d*_6_) δ ppm: 2.54 (s, 3H), 3.82 (s, 3H), 3.93 (s, 3H), 6.58 (d, J = 16.0 Hz, 1H), 7.21–7.24 (m, 1H), 7.39 (s, 1H), 7.44–7.50 (m, 2H), 7.55 (d, J = 8.4 Hz, 1H), 7.71 (d, J = 16.0 Hz, 1H), 7.99 (s, 1H). ^13^C NMR (400 MHz, DMSO-*d*_6_) δ ppm: 10.34, 55.80, 56.89, 95.69, 114.48, 118.79, 120.22, 121.21, 121.96, 122.11, 122.31, 127.86, 130.10, 133.70, 139.35, 148.12, 156.14, 159.54, 159.85, 163.50, 184.57. MS (ESI) *m*/*z* calcd. for C_21_H_19_NO_6_ [M + H]^+^: 382.38, found: 382.22. Anal. calcd for C_21_H_19_NO_6_. C, 66.13; H, 5.02; N, 3.67; found: C, 66.24; H, 5.13; N, 3.78.

(*E*)-*N*-Hydroxy-3-(6-methoxy-2-(2-methoxybenzoyl)-3-methylbenzofuran-5-yl)acrylamide **6f**. 

Following general procedure E, compound **6f** was obtained as a yellow solid. Yield: 84%, mp 122–124 °C. ^1^H NMR (400 MHz, DMSO-*d*_6_) δ ppm: 2.37 (s, 3H), 3.72 (s, 3H), 3.90 (s, 3H), 6.57 (d, J = 16.0 Hz, 1H), 7.07 (t, J = 7.4 Hz, 1H), 7.19 (d, J = 8.4 Hz, 1H), 7.32 (s, 1H), 7.36 (dd, J = 7.4 and 1.6 Hz, 1H), 7.49–7.57 (m, 1H), 7.72 (d, J = 16.0 Hz, 1H), 7.96 (s, 1H), 10.70 (s, 1H). ^13^C NMR (400 MHz, DMSO-*d*_6_) δ ppm: 9.75, 56.29, 56.86, 95.54, 112.55, 120.09, 121.05, 121.35, 121.94, 122.51, 126.30, 128.81, 129.43, 132.63, 133.75, 148.65, 156.20, 157.27, 159.85, 163.54, 185.84. MS (ESI) *m*/*z* calcd. for C_21_H_19_NO_6_ [M + H]^+^: 382.38, found: 382.28. Anal. calcd for C_21_H_19_NO_6_. C, 66.13; H, 5.02; N, 3.67; found: C, 66.24; H, 5.15; N, 3.81.

(*E*)-3-(2-Benzoyl-6-methoxy-3-methylbenzofuran-5-yl)-*N*-hydroxyacrylamide **6g**. 

Following general procedure E, compound **6g** was obtained as a yellowish solid. Yield: 64%, mp 150 °C. ^1^H NMR (400 MHz, DMSO-*d*_6_) δ ppm: 2.54 (s, 3H), 3.92 (s, 3H), 6.61 (d, J = 16.0 Hz, 1H), 7.38 (s, 1H), 7.56 (t, J = 8.0 Hz, 2H), 7.64–7.69 (m, 1H), 7.75 (d, J = 16.0 Hz, 1H), 7.94 (dd, J = 8.4 and 1.6 Hz, 2H), 8.00 (s, 1H). ^13^C NMR (400 MHz, DMSO-*d*_6_) δ ppm: 10.31, 56.87, 95.67, 120.20, 121.24, 122.07, 122.30, 122.75, 128.92 (2C), 129.59 (2C), 133.05, 133.70, 138.06, 148.17, 156.13, 159.81, 163.49, 184.94. MS (ESI) *m*/*z* calcd. for C_20_H_17_NO_5_ [M + H]^+^: 352.11, found: 352.17. Anal. calcd for C_20_H_17_NO_5_. C, 68.37; H, 4.88; N, 3.99; found: C, 68.51; H, 4.96; N, 4.12.

(*E*)-*N*-Hydroxy-3-(3-methyl-2-(3,4,5-trimethoxybenzoyl)benzofuran-5-yl)acrylamide **6h**.

Following general procedure E, compound **6h** was obtained as a white solid. Yield: 51%, mp 188–190 °C. ^1^H NMR (400 MHz, DMSO-*d*_6_) δ ppm: 2.56 (s, 3H), 3.79 (s, 3H), 3.86 (s, 6H), 6.57 (d, J = 15.8 Hz, 1H), 7.34 (s, 2H), 7.62 (d, J = 15.8 Hz, 1H), 7.73–7.78 (m, 2H), 8.07 (s, 1H), 10.28 (s, 1H). ^13^C NMR (400 MHz, DMSO-*d*_6_) δ ppm: 10.28, 56.53 (2C), 60.68, 107.57 (2C), 113.30, 119.39, 121.82, 126.59, 128.15, 129.63, 131.28, 132.72, 138.49, 142.20, 148.70, 153.08 (2C), 154.60, 163.18, 184.23. MS (ESI) *m*/*z* calcd. for C_22_H_21_NO_7_ [M + H]^+^: 412.41, found: 412.33. Anal. calcd for C_22_H_21_NO_7_. C, 64.23; H, 5.14; N, 3.40; found: C, 64.31; H, 5.25; N, 3.53.

(*E*)-*N*-Hydroxy-3-(6-methoxy-2-(3,4,5-trimethoxybenzoyl)benzofuran-5-yl)acrylamide **6i**.

Following general procedure E, the desired compound **6i** was obtained as a yellow solid. Yield: 90%, mp 116–118 °C. ^1^H NMR (400 MHz, DMSO-*d*_6_) δ ppm: 3.77 (s, 3H), 3.87 (s, 6H), 3.96 (s, 3H), 6.51 (d, J = 15.8 Hz, 1H), 7.26 (s, 2H), 7.49 (s, 1H), 7.74 (d, J = 15.8 Hz, 1H), 7.83 (s, 1H), 7.96 (s, 1H), 9.02 (s, 1H), 10.76 (s, 1H). ^13^C NMR (400 MHz, DMSO-*d*_6_) δ ppm: 56.58 (2C), 60.66, 65.37, 95.78, 107.14 (2C), 118.19, 120.00, 120.61, 122.47, 122.73, 132.51, 133.66, 142.06, 151.79, 153.22 (2C), 157.88, 159.58, 163.40, 182.17. MS (ESI) *m*/*z* calcd. for C_22_H_21_NO_8_ [M + H]^+^: 428.41, found: 428.40. Anal. calcd for C_22_H_21_NO_8_. C, 61.82; H, 4.95; N, 3.28; found: C, 61.93; H, 5.04; N, 3.36. 

#### 4.1.6. 6-Methoxy-3-methyl-5-(2-trimethylsilylethynyl)-1-benzofuran-2-yl]-(3,4,5-trimethoxyphenyl) methanone **7**

A mixture of (5-bromo-6-methoxy-3-methyl-1-benzofuran-2-yl)-(3,4,5-trimethoxy phenyl)methanone **2a** (200 mg, 0.460 mmol), ethynyl(trimethyl)silane (0.1 mL, 0.690 mmol), triethylamine (0.19 mL, 1.38 mmol), bis(triphenylphosphine)palladium(II) dichloride [PdCl_2_(PPh_3_)_2_] (32.25 mg, 0.050 mmol), and copper (I) iodide (8.75 mg, 0.050 mmol) in THF (4 mL) was degassed and then submitted to a microwave reactor (5 min. at 100 °C). Ethynyl(trimethyl)silane (0.02 mL, 0.170 mmol), triethylamine (0.05 mL, 0.340 mmol), PdCl_2_(PPh_3_)_2_ (8.06 mg, 0.010 mmol), and copper (I) iodide (2.19 mg, 0.010 mmol) were added again: the reaction mixture was degassed and then submitted to a microwave reactor (5 min at 100 °C). The mixture was diluted with DCM, filtered, and concentrated. The residue was purified via flash chromatography (silica 25 g+ 25 g, cyclohexane (Cy)/EtOAc from 10:0 to 6:4) affording the target compound **7** as a yellowish foam. Yield: 57%, mp 122–124 °C. ^1^H NMR (400 MHz, DMSO-*d*_6_) δ ppm 0.25 (s, 9H), 2.52 (s, 3H), 3.79 (s, 3H), 3.86 (s, 6H), 3.92 (s, 3H), 7.33 (s, 2H), 7.45 (s, 1H), 7.94 (s, 1H). MS (ESI): [M + 1]^+^ = 453.27.

#### 4.1.7. 5-Ethynyl-6-methoxy-3-methyl-benzofuran-2-yl-(3,4,5-trimethoxyphenyl)methanone **8**

Tetrabutylammonium fluoride (0.22 mL, 0.220 mmol) was added to a stirred solution of compound **7** (118.0 mg, 0.220 mmol) in THF (3 mL). The reaction mixture was stirred at room temperature for 30 min, then brine and EtOAc were added; phases were separated, and the organic phase was dried over sodium sulfate, filtered, and concentrated. The crude product was purified via flash chromatography (silica 10 g + 10 g, Cy/EtOAc from 10:0 to 6:4) affording (5-ethynyl-6-methoxy-3-methyl-benzofuran-2-yl)-(3,4,5-trimethoxyphenyl)methanone **8** as a yellowish solid. Yield: 52%, mp 186–188 °C. ^1^H NMR (400 MHz, DMSO-*d*_6_) δ ppm 2.51 (s, 3H), 3.77 (s, 3H), 3.85 (s, 6H), 3.89 (s, 3H), 4.25 (s, 1H), 7.30 (s, 2H), 7.43 (s, 1H), 7.93 (s, 1H). ^13^C NMR (400 MHz, DMSO-*d*_6_) δ ppm: 9.77, 56.00 (2C), 56.35, 60.10, 79.72, 83.97, 95.22, 106.87 (2C), 108.66, 121.50, 126.54, 126.58, 132.45, 141.38, 147.71, 152.48 (2C), 154.96, 161.37, 183.06. MS (ESI) *m*/*z* calcd. for C_22_H_20_NO_6_ [M + H]^+^: 381.40, found: 381.26. Anal. calcd for C_22_H_20_O_6_. C, 69.46; H, 5.30; found: C, 69.62; H, 5.46

#### 4.1.8. General Procedure F for Preparing Intermediates **9a**–**f**

A solution of propynoic acid (39 μL, 0.630 mmol, 1.1 equiv.) in DMSO (0.4 mL) was added to a mixture of 2-aroyl-5-bromo-3-methylbenzofurane **2a**–**f** (0.570 mmol), palladium tetrakis triphenylphosphine (46.46 mg, 0.040 mmol, 7 mol %), and 2,3,4,6,7,8,9,10-octahydropyrimido[1,2-*a*]azepine (0.26 mL, 1.72 mmol, 3 equiv.) in DMSO (2 mL). The reaction mixture was stirred overnight at 40 °C and then at room temperature for 2 days. The reaction mixture was poured into EtOAc and then a saturated aqueous solution of sodium bicarbonate (NaHCO_3_) was added. The phases were separated, the aqueous phase was acidified with 1 N HCl, and then it was extracted via DCM. The organic layer was dried over Na_2_SO_4_ and concentrated under reduced pressure to afford the corresponding Sonogashira’s coupling products **9a**–**f**, which were used for the next reaction without further purification.

3-[6-Methoxy-3-methyl-2-(3,4,5-trimethoxybenzoyl)-1-benzofuran-5-yl]prop-2-ynoic acid **9a**.

Following general procedure F, compound **9a** was obtained as a yellow solid. Yield: 92%, mp 212–214 °C. ^1^H NMR (400 MHz, DMSO-*d*_6_) δ ppm: 2.54 (s, 3H), 3.80 (s, 3H), 3.88 (s, 6H), 3.96 (s, 3H), 7.33 (s, 2H), 7.53 (s, 1H), 8.13 (s, 1H), 12.6 (bs, 1H). MS (ESI): [M + 1]^+^ = 425.10.

3-[2-(3,4-Dimethoxybenzoyl)-6-methoxy-3-methyl-1-benzofuran-5-yl]prop-2-ynoic acid **9b**.

Following general procedure F, compound **9b** was isolated as a yellow solid. Yield: 58%, mp 245–247 °C. ^1^H NMR (400 MHz, DMSO-*d*_6_) δ ppm: 2.55 (s, 3H), 3.85 (s, 3H), 3.91 (s, 3H), 3.94 (s, 3H), 7.17 (s, 1H), 7.46–7.51 (m, 1H), 7.55–7.59 (m, 1H), 7.79 (s, 1H), 8.14 (s, 1H), 13.1 (bs, 1H). MS (ESI): [M + 1]^+^ = 395.17.

3-[2-(3,5-Dimethoxybenzoyl)-6-methoxy-3-methyl-1-benzofuran-5-yl]prop-2-ynoic acid **9c**.

Following general procedure F, derivative **9c** was obtained as a brownish solid. Yield: 53%, mp 288–290 °C. ^1^H NMR (400 MHz, DMSO-*d*_6_) δ ppm: 2.54 (s, 3H), 3.84 (s, 6H), 3.96 (s, 3 H), 6.82 (t, J = 2.3 Hz, 1 H), 7.10 (d, J = 2.2 Hz, 2H), 7.52 (s, 1H), 8.13 (s, 1 H), 13.0 (bs, 1H). MS (ESI): [M + 1]^+^ = 395.20.

3-[6-Methoxy-2-(4-methoxybenzoyl)-3-methyl-1-benzofuran-5-yl]prop-2-ynoic acid **9d**.

Following general procedure F, compound **9d** was isolated as a yellow solid. Yield: 77%, mp 204–206 °C. ^1^H NMR (400 MHz, DMSO-*d*_6_) δ ppm: 2.54 (s, 3H), 3.89 (s, 3H), 3.92 (s, 3H), 7.10 (d, J = 8.4 Hz, 2H), 7.36 (s, 1H), 7.85 (s, 1H), 8.05 (d, J = 8.4 Hz, 2H), 13.2 (bs, 1H). MS (ESI): [M + 1]^+^ = 365.16.

3-[6-Methoxy-2-(3-methoxybenzoyl)-3-methyl-1-benzofuran-5-yl]prop-2-ynoic acid **9e**.

Following general procedure F, derivative **9e** was obtained as a yellow solid. Yield: 77%, mp 234–236 °C. ^1^H NMR (400 MHz, DMSO-*d*_6_) δ ppm: 2.54 (s, 3H), 3.88 (s, 3H), 3.94 (s, 3H), 7.24 (s, 1H), 7.46–7.54 (m, 3H), 7.56–7.60 (m, 1H), 8.14 (s, 1H), 13.53 (s, 1H). MS (ESI): [M + 1]^+^ = 365.11.

3-[6-Methoxy-2-(2-methoxybenzoyl)-3-methyl-1-benzofuran-5-yl]prop-2-ynoic acid **9f**.

Following general procedure F, compound **9f** was obtained as a yellow solid. Yield: 93%, mp 266–268 °C. ^1^H NMR (400 MHz, DMSO-*d*_6_) δ ppm: 2.38 (s, 3H), 3.74 (s, 3H), 3.93 (s, 3H), 7.07–7.12 (m, 1H), 7.21 (dd, J = 8.2 and 0.8 Hz, 1H), 7.37–7.40 (m, 1H), 7.41 (s, 1H), 7.53–7.59 (m, 1H), 8.08 (1 H, s), 13.2 (bs, 1H). MS (ESI): [M + 1]^+^ = 365.14.

#### 4.1.9. 3-(2-Benzoyl-6-methoxy-3-methyl-benzofuran-5-yl)prop-2-ynoic **9g**

A solution of propynoic acid (33 μL, 0.540 mmol) in DMSO (0.2 mL) was added to a mixture of (5-bromo-6-methoxy-3-methyl-benzofuran-2-yl)-phenyl-methanone **2g** (170.0 mg, 0.490 mmol), palladium tetrakis triphenylphosphine (39.84 mg, 0.030 mmol), and 2,3,4,6,7,8,9,10-octahydropyrimido[1,2-a]azepine (0.22 mL, 1.48 mmol) in DMSO (1 mL). The reaction mixture was stirred overnight at 45 °C. Propynoic acid (33 μL, 0.540 mmol) in DMSO (0.2 mL), 2,3,4,6,7,8,9,10-octahydropyrimido[1,2-a]azepine (0.22 mL, 1.48 mmol), and palladium tetrakis triphenylphosphine (39.84 mg, 0.030 mmol) were added again; the mixture was degassed and then stirred at 45 °C overnight. The reaction mixture was poured into EtOAc and then a saturated aqueous solution of NaHCO_3_ was added. The phases were separated, and the aqueous phase was acidified with 6 N HCl and then extracted via DCM. The phases were separated, and the organic phase was dried over Na_2_SO_4_ and concentrated under reduced pressure to afford 3-(2-benzoyl-6-methoxy-3-methyl-benzofuran-5-yl)prop-2-ynoic acid **9g** as a brownish solid. Yield: 97%, mp 244–246 °C. ^1^H NMR (400 MHz, DMSO-*d*_6_) δ ppm: 2.52 (s, 3H), 3.89 (s, 3H), 7.08 (s, 1H), 7.50–7.52 (m, 2H), 7.64–7.66 (m, 1H), 7.88 (s, 1H), 8.08 (d, J = 7.2 Hz, 2H). 12.8 (bs, 1H). MS (ESI): [M + 1]^+^ = 335.09.

#### 4.1.10. 3-[3-Methyl-2-(3,4,5-trimethoxybenzoyl)benzofuran-5-yl]prop-2-ynoic acid **9h**

A solution of propynoic acid (50 μL, 0.810 mmol) in DMSO (0.400 mL) was added to a mixture of (5-bromo-3-methyl-benzofuran-2-yl)-(3,4,5-trimethoxyphenyl)methanone **2h** (300.0 mg, 0.740 mmol), palladium tetrakis triphenylphosphine (59.88 mg, 0.050 mmol), and 2,3,4,6,7,8,9,10-octahydropyrimido[1,2-*a*]azepine (0.33 mL, 2.22 mmol) in DMSO (2 mL). The reaction mixture was stirred overnight at 45 °C; then, 2,3,4,6,7,8,9,10-octahydropyrimido[1,2-*a*]azepine (0.33 mL, 2.22 mmol), palladium tetrakis triphenylphosphine (59.88 mg, 0.050 mmol), and propynoic acid (50 μL, 0.810 mmol) in DMSO (0.400 mL) were added. The mixture was degassed and then stirred overnight at 45 °C. The reaction mixture was poured into EtOAc and then a saturated aqueous solution of NaHCO_3_ was added. The two phases were separated, and to the aqueous phase was added cold 6 M HCl and then DCM; the phases were separated, and the organic phase was dried over sodium sulfate, filtered, and concentrated, to furnish **9h** as a brown oil. Yield: 66%. ^1^H NMR (400 MHz, DMSO-*d*_6_) δ ppm: 2.56 (s, 3H), 3.80 (s, 6H), 3.86 (s, 3H), 7.34 (s, 2H), 7.76–7.81 (m, 1H), 7.83–7.87 (m, 1H), 8.25 (d, J = 2.0 Hz, 1H), 13.83 (bs, 1H). MS (ESI): [M + 1]^+^ = 395.17.

#### 4.1.11. General Procedure G for Preparing Intermediates **10a**–**h**

A mixture of derivative **9a**–**h** (1 mmol), 1-hydroxybenzotriazole hydrate (HOBt) (254 mg, 1.6 mmol, 1.6 equiv.), and 1-ethyl-3-(3-dimethylaminopropyl)carbodiimide hydrochloride (EDCI) (244 mg, 1.28 mmol, 1.3 equiv.) in DMF (10 mL) was stirred at room temperature for 30 min; then, O-(tetrahydro-2*H*-pyran-2-yl)hydroxylamine (NH_2_OTHP) (186 mg, 1.6 mmol, 1.6 equiv.) was added, and the stirring was continued at 50 °C for 2.5 h. Volatiles were removed under vacuum, the crude residue was dissolved in DCM, and the organic layer was washed with a saturated aqueous solution of NaHCO_3_ and the phases separated; the organic phase was washed with brine, dried over sodium sulfate, filtered, and concentrated. The residue was purified via column chromatography on silica gel using the appropriate mixture of ethyl acetate and petroleum ether as eluent to furnish derivatives **10a**–**h**.

3-[6-Methoxy-3-methyl-2-(3,4,5-trimethoxybenzoyl) -1-benzofuran-5-yl]-*N*-(oxan-2-yloxy)prop-2-ynamide **10a**.

Following general procedure G, the crude residue was purified via flash chromatography, using ethyl acetate: petroleum ether from 2:8 to 1:1 (*v*:*v*) as eluent, to furnish **10a** as an orange solid. Yield: 45%, mp 132–134 °C. ^1^H NMR (400 MHz, DMSO-*d*_6_) δ ppm: 2.54 (s, 3H), 2.74 (d, J = 0.66 Hz, 4H), 2.88–2.91 (m, 4H), 3.80 (s, 3H), 3.88 (s, 6H), 3.95 (s, 3H), 4.95 (bs, 1H), 5.97 (bs, 1H), 7.33 (s, 2H), 7.54 (s, 1H), 7.96 (s, 1H). MS (ESI): [M + 1]^+^ = 524.25.

3-[2-(3,4-Dimethoxybenzoyl)-6-methoxy-3-methyl-1-benzofuran-5-yl]-*N*-(oxan-2-yloxy)prop-2-ynamide **10b**.

Following general procedure G, the crude residue was purified via flash chromatography, using ethyl acetate: petroleum ether from 1:9 to 2:8 (*v*:*v*) as eluent, to furnish **10b** as a yellowish foam. Yield: 61%, mp 114–116 °C. ^1^H NMR (400 MHz, DMSO-*d*_6_) δ ppm: 1.55 (bs, 4H), 1.70 (d, J = 2.0 Hz, 3H), 2.52 (s, 3H), 3.54 (d, J = 11.00 Hz, 1H), 3.87 (s, 3H), 3.88 (s, 3H), 3.98 (s, 3H). 4.95 (t, J = 2.8 Hz, 1H), 7.13–7.17 (m, 1H), 7.50–7.52 (m, 1H), 7.57 (d, J = 2.0 Hz, 1H), 7.74–7.78 (m, 1H), 8.08 (s, 1H), 11.82 (bs, 1H). MS (ESI): [M + 1]^+^ = 494.25.

3-[2-(3,5-Dimethoxybenzoyl)-6-methoxy-3-methyl-1-benzofuran-5-yl]-*N*-(oxan-2-yloxy)prop-2-ynamide **10c**.

Following general procedure G, the crude residue was purified via flash chromatography, using ethyl acetate: petroleum ether from 1:9 to 2:8 (*v*:*v*) as eluent, to furnish **10c** as a yellowish foam. Yield: 37%, mp 146–148 °C. ^1^H NMR (400 MHz, DMSO-*d*_6_) δ ppm: 2.54 (s, 3H), 3.84 (s, 6H), 3.96 (s, 3 H), 6.82 (t, J = 2.3 Hz, 1 H), 7.10 (d, J = 2.2 Hz, 2H), 7.52 (s, 1H), 8.13 (s, 1 H), 13.0 (bs, 1H). MS (ESI): [M + 1]^+^ = 494.11.

3-[6-Methoxy-2-(4-methoxybenzoyl)-3-methyl-1-benzofuran-5-yl]-*N*-(oxan-2-yloxy)prop-2-ynamide **10d**.

Following general procedure G, the crude residue was purified via flash chromatography, using ethyl acetate: petroleum ether from 1:9 to 3:7 (*v*:*v*) as eluent, to furnish **10d** as a yellowish foam. Yield: 52%, mp 140–142 °C. ^1^H NMR (400 MHz, DMSO-*d*_6_) δ ppm: 1.54 (bs, 3H), 1.69 (bs, 3H), 2.53 (s, 3H), 3.53 (d, J = 13.2 Hz, 2H), 3.87–3.91 (m, 3H), 3.94 (s, 3H), 4.94 (bs, 1H), 7.10–7.15 (m, 2H), 7.48–7.52 (m, 1H), 8.04–8.09 (m, 3H), 11.82 (bs, 1H). MS (ESI): [M + 1]^+^ = 464.17.

3-[6-Methoxy-2-(3-methoxybenzoyl)-3-methyl-1-benzofuran-5-yl]-*N*-(oxan-2-yloxy)prop-2-ynamide **10e**.

Following general procedure G, the crude residue was purified via flash chromatography, using ethyl acetate: petroleum ether from 1:9 to 3:7 (*v*:*v*) as eluent, to furnish **10e** as a brownish oil. Yield: 56%. ^1^H NMR (400 MHz, DMSO-*d*_6_) δ ppm: 1.55 (bs, 4H), 1.65–1.73 (m, 3H), 2.58 (s, 3H), 3.54 (d, J = 10.2 Hz, 1H), 3.85 (s, 3H), 3.93 (s, 3H), 4.94 (bs, 1H), 7.27 (ddd, J = 8.14, 2.64, 1.10 Hz, 1H), 7.48 (dd, J = 2.6 and 1.4 Hz, 1H), 7.49–7.54 (m, 2H), 7.56–7.59 (m, 1H), 8.10 (s, 1H), 11.82 (bs, 1H). MS (ESI): [M + 1]^+^ = 464.18.

3-[6-Methoxy-2-(2-methoxybenzoyl)-3-methyl-1-benzofuran-5-yl]-*N*-(oxan-2-yloxy)prop-2-ynamide **10f**.

Following general procedure G, the crude residue was purified via flash chromatography, using ethyl acetate: petroleum ether from 1:9 to 2:8 (*v*:*v*) as eluent, to furnish **10f** as a yellowish oil. Yield: 42%. ^1^H NMR (400 MHz, DMSO-*d*_6_) δ ppm: 1.54 (bs, 3H), 1.69 (bs, 3H), 2.38 (s, 3H), 3.74 (s, 3H), 3.89 (s, 3H), 7.07–7.12 (m, 4H), 7.21 (dd, J = 8.4 and 0.66 Hz, 1H), 7.38 (dd, J = 7.48 and 1.76 Hz, 1H), 7.42 (s, 1H), 7.56 (td, J = 8.0 and 1.8 Hz, 1H), 8.02–8.08 (m, 1H), 11.80 (bs, 1H). MS (ESI): [M + 1]^+^ = 464.16.

3-(2-Benzoyl-6-methoxy-3-methyl-benzofuran-5-yl)-*N*-tetrahydropyran-2-yloxy-prop-2-ynamide **10g**.

Following general procedure G, the crude residue was purified via flash chromatography, using ethyl acetate: petroleum ether from 1:9 to 3:7 (*v*:*v*) as eluent, to furnish **10g** as a yellowish foam. Yield: 34%, mp 162–164 °C. ^1^H NMR (400 MHz, DMSO-*d*_6_) δ ppm: 1.55 (bs, 4H), 1.69 (bs, 3H), 2.55 (s, 3H), 3.54 (d, J = 11.8 Hz, 1H), 3.96 (s, 3H), 4.95 (t, J = 2.64 Hz, 1H), 7.49–7.52 (m, 1H), 7.57–7.63 (m, 2H), 7.67–7.72 (m, 1H), 7.95–8.02 (m, 2H), 8.09–8.13 (m, 1H), 11.8 (bs, 1H). MS (ESI): [M + 1]^+^ = 434.27.

3-[3-Methyl-2-(3,4,5-trimethoxybenzoyl)benzofuran-5-yl]-*N*-tetrahydropyran-2-yloxy-prop-2-ynamide **10h**.

Following general procedure G, the crude residue was purified via flash chromatography, using ethyl acetate: petroleum ether from 1:9 to 3:7 (*v*:*v*) as eluent, to furnish **10h** as a yellowish foam. Yield: 33%, mp 143–145 ° ^1^H NMR (400 MHz, DMSO-*d*_6_) δ ppm: 1.51–1.59 (m, 4H), 1.67–1.73 (m, 3H), 2.56 (s, 3H), 3.55 (d, J = 11.00 Hz, 1H), 3.79–3.82 (m, 3H), 3.85–3.90 (m, 6H), 4.95 (bs, 1H), 7.35 (s, 2H), 7.73–7.78 (m, 1H), 7.83–7.88 (m, 1H), 8.18–8.22 (m, 1H), 11.85 (bs, 1H). MS (ESI): [M + 1]^+^ = 494.27.

#### 4.1.12. General Procedure H for Preparing Target Compounds **11a**–**h**

A mixture of compound **10a**–**h** (0.55 mmol) and 4-methylbenzenesulfonic acid hydrate (22 mg, 0.1 mmol) in methanol (10 mL) was stirred at 50 °C for 2 h; then, the solvent was removed through evaporation, and the residue was purified via flash chromatography on reverse phase.

*N*-Hydroxy-3-[6-methoxy-3-methyl-2-(3,4,5-trimethoxybenzoyl)-1-benzofuran-5-yl] prop-2-ynamide **11a**.

Following general procedure H, the residue was purified via flash chromatography, reverse phase (silica c18 6 g, water + 0.1% formic acid/MeCN + 0.1% formic acid, from 98:2 to 1:1), and afforded **11a** as a yellowish solid. Yield: 49%, mp 268–270 °C. ^1^H NMR (400 MHz, DMSO-*d*_6_) δ ppm: 2.55 (s, 3H), 3.80 (s, 3H), 3.88 (s, 6H), 3.96 (s, 3H), 7.30 (s, 2H), 7.51 (s, 1H), 8.02 (s, 1H), 9.22 (bs, 1H), 11.27 (bs, 1H). ^13^C NMR (400 MHz, DMSO-*d*_6_) δ ppm: 9.73, 56.02 (2C), 56.54, 60.11, 81.67, 84.97, 95.58, 106.63, 106.90 (2C), 121.81, 126.45, 127.32, 132.34, 141.45, 147.92, 150.12, 152.50 (2C), 155.68, 161.40, 183.11. MS (ESI) *m*/*z* calcd. for C_23_H_21_NO_8_ [M + H]^+^: 440.13, found: 440.17. Anal. calcd for C_23_H_21_NO_8_. C, 62.87; H, 4.82; N, 3.19; found: C, 62.96; H, 4.93; N, 3.32.

3-[2-(3,4-Dimethoxybenzoyl)-6-methoxy-3-methyl-1-benzofuran-5-yl]-*N*-hydroxyprop-2-ynamide **11b**.

Following general procedure H, the residue was purified via flash chromatography, reverse phase (silica c18 12 g + 12 g, water + 0.1% formic acid/MeCN + 0.1% formic acid, from 98:2 to 45:55), affording compound **11b** as a yellowish solid. Yield: 65%, mp 276–277 °C. ^1^H NMR (400 MHz, DMSO-*d*_6_) δ ppm: 2.52 (s, 3H), 3.85 (s, 3H), 3.88 (s, 3H), 3.93 (s, 3H), 7.11 (d, J = 8.4 Hz, 1H), 7.48 (s, 1H), 7.53 (d, J = 2.0 Hz, 1H), 7.72 (dd, J = 8.4 and 2.0 Hz, 1H), 8.00 (s, 1H), 9.25 (bs, 1H), 11.25 (bs, 1H). ^13^C NMR (400 MHz, DMSO-*d*_6_) δ ppm: 9.62, 55.47, 55.69, 56.50, 81.77, 84.86, 95.53, 106.46, 110.74, 111.77, 121.82, 124.17, 125.69, 127.21, 129.60, 146.72, 148.40, 150.15, 152.91, 155.52, 161.23, 182.47. MS (ESI) *m*/*z* calcd. for C_22_H_19_NO_7_ [M + H]^+^: 410.12, found: 410.19. Anal. calcd for C_22_H_19_NO_7_. C, 64.54; H, 4.68; N, 3.42; found: C, 64.67; H, 4.83; N, 3.56.

3-[2-(3,5-Dimethoxybenzoyl)-6-methoxy-3-methyl-1-benzofuran-5-yl]-*N*-hydroxyprop-2-ynamide **11c**.

Following general procedure H, the residue was purified via flash chromatography, reverse phase (silica c18 12 g + 12 g, water + 0.1% formic acid/MeCN + 0.1% formic acid, from 98:2 to 1:1), affording compound **11c** as a yellowish solid. Yield: 35%, mp 248–250 °C. ^1^H NMR (400 MHz, DMSO-*d*_6_) δ ppm: 2.52 (s, 3H), 3.83 (s, 6H), 3.93 (s, 3H), 6.81 (t, J = 2.31 Hz, 1H), 7.09 (d, J = 2.20 Hz, 2H), 7.51 (s, 1H), 8.03 (s, 1H), 9.26 (bs, 1H), 11.25 (bs, 1H). ^13^C NMR (400 MHz, DMSO-*d*_6_) δ ppm: 9.70, 55.43 (2C), 56.56, 81.67, 84.90, 95.59, 104.18, 106.65, 106.86 (2C), 121.76, 126.93, 127.42, 139.15, 147.80, 150.12, 155.72, 160.20 (2C), 161.51, 183.84. MS (ESI) *m*/*z* calcd. for C_22_H_19_NO_7_ [M + H]^+^: 410.12, found: 410.16. Anal. calcd for C_22_H_19_NO_7_. C, 64.54; H, 4.68; N, 3.42; found: C, 64.71; H, 4.85; N, 3.57.

*N*-Hydroxy-3-[6-methoxy-2-(4-methoxybenzoyl)-3-methyl-1-benzofuran-5-yl]prop-2-ynamide **11d**.

Following general procedure H, the crude residue was purified via flash chromatography, reverse phase (silica c18 12 g + 12 g, water + 0.1% formic acid/MeCN + 0.1% formic acid, from 98:2 to 45:55), affording compound **11d** as a yellow solid. Yield: 52%, mp 222–224 °C. ^1^H NMR (400 MHz, DMSO-*d*_6_) δ ppm: 2.56 (s, 3H), 3.89 (s, 3H), 3.94 (s, 3H), 7.09 (d, J = 9.2 Hz, 2H), 7.46 (s, 1H), 8.02–8.09 (m, 3H), 9.27 (bs, 1H), 11.25 (bs, 1H). ^13^C NMR (400 MHz, DMSO-*d*_6_) δ ppm: 10.23, 56.13, 57.15, 82.41, 85.52, 96.18, 107.13, 114.41 (2C), 122.46, 126.43, 127.88, 130.36, 132.34 (2C), 148.92, 150.79, 156.14, 161.89, 163.57, 183.30. MS (ESI) *m*/*z* calcd. for C_21_H_17_NO_6_ [M + H]^+^: 380.11, found: 380.17. Anal. calcd for C_21_H_17_NO_6_. C, 66.49; H, 4.52; N, 3.69; found: C, 66.62; H, 4.64; N, 3.81.

*N*-Hydroxy-3-[6-methoxy-2-(3-methoxybenzoyl)-3-methyl-1-benzofuran-5-yl]prop-2-ynamide **11e**.

Following general procedure H, the crude residue was purified via flash chromatography, reverse phase (silica c18 12 g + 12 g, water + 0.1% formic acid/MeCN + 0.1% formic acid, from 98:2 to 40:60), affording compound **11e** as a yellowish solid. Yield: 52%, mp 222–224 °C. ^1^H NMR (400 MHz, DMSO-*d*_6_) δ ppm: 2.56 (s, 3H), 3.84 (s, 3H), 3.92 (s, 3H), 7.22–7.25 (m, 1H), 7.45–7.53 (m, 3H), 7.55–7.60 (m, 1H), 8.02–8.07 (m, 1H), 9.26 (bs, 1H), 11.25 (bs, 1H). ^13^C NMR (400 MHz, DMSO-*d*_6_) δ ppm: 10.32, 55.91, 57.20, 82.33, 85.56, 96.22, 107.30, 114.59, 119.02, 122.07, 122.42, 127.46, 128.07, 130.23, 139.27, 148.56, 150.77, 156.36, 159.65, 162.12, 184.71. MS (ESI) *m*/*z* calcd. for C_21_H_17_NO_6_ [M + H]^+^: 380.11, found: 380.11. Anal. calcd for C_21_H_17_NO_6_. C, 66.49; H, 4.52; N, 3.69; found: C, 66.58; H, 4.62; N, 3.80.

*N*-Hydroxy-3-[6-methoxy-2-(2-methoxybenzoyl)-3-methyl-1-benzofuran-5-yl]prop-2-ynamide **11f**.

Following general procedure H, the crude residue was purified via flash chromatography, reverse phase (silica c18 12 g + 12 g, water + 0.1% formic acid/MeCN + 0.1% formic acid, from 98:2 to 45:55), affording compound **11f** as a yellowish foam. Yield: 36%, mp 243–245 °C. ^1^H NMR (400 MHz, DMSO-*d*_6_) δ ppm: 2.34 (s, 3H), 3.75 (s, 3H), 3.87 (s, 3H), 7.06–7.12 (m, 1H), 7.20 (d, J = 8.2 Hz, 1H), 7.37 (dd, J = 7.5 and 1.8 Hz, 1H), 7.41 (s, 1H), 7.53–7.58 (m, 1H), 8.00 (s, 1H), 9.20–9.33 (m, 1H), 11.23 (bs, 1H). ^13^C NMR (101 MHz, DMSO-*d*_6_) δ 9.26, 55.85, 56.42, 95.54, 112.11, 120.07, 120.61, 121.26, 121.42, 122.45, 125.83, 128.41, 128.88, 132.15, 132.28, 148.07, 155.55, 156.86, 157.97, 164.78, 185.42. MS (ESI) *m*/*z* calcd. for C_21_H_17_NO_6_ [M + H]^+^: 380.11, found: 380.15. Anal. calcd for C_21_H_17_NO_6_. C, 66.49; H, 4.52; N, 3.69; found: C, 66.61; H, 4.64; N, 3.82.

3-(2-Benzoyl-6-methoxy-3-methyl-benzofuran-5-yl)prop-2yne hydroxamic acid **11g**.

Following general procedure H, the crude residue was purified via flash chromatography, reverse phase (silica c18 12 g + 12 g, water + 0.1% formic acid/MeCN + 0.1% formic acid, from 98:2 to 1:1), affording compound **5g** as a yellowish solid. Yield: 44%, mp 205–206 °C. ^1^H NMR (400 MHz, DMSO-*d*_6_) δ ppm: 2.57 (s, 3H), 3.94 (s, 3H), 7.48 (s, 1H), 7.57–7.61 (m, 2H), 7.66–7.71 (m, 1H), 7.96–8.00 (m, 2H), 8.05 (s, 1H), 9.24 (bs, 1H), 11.17 (bs, 1H). ^13^C NMR (400 MHz, DMSO-*d*_6_) δ ppm: 9.62, 56.53, 82.35, 84.91, 95.56, 106.64, 121.78, 126.68, 127.43, 128.39 (2C), 129.05 (2C), 132.60, 137.34, 147.96, 150.12, 155.69, 161.44, 184.43. MS (ESI) *m*/*z* calcd. for C_20_H_15_NO_5_ [M + H]^+^: 350.10, found: 350.17. Anal. calcd for C_20_H_15_NO_5_. C, 68.76; H, 4.33; N, 4.01; found: C, 68.91; H, 4.42; N, 4.12.

3-[3-Methyl-2-(3,4,5-trimethoxybenzoyl)benzofuran-5-yl]prop-2-yne hydroxamic acid **11h**.

Following general procedure H, the crude residue was purified via flash chromatography, reverse phase (silica c18 12 g + 12 g, water + 0.1% formic acid/MeCN + 0.1% formic acid, from 98:2 to 45:55), affording compound **11h** as a yellowish solid. Yield: 42%, mp 254–256 °C. ^1^H NMR (400 MHz, DMSO-*d*_6_) δ ppm: 2.54 (s, 3H), 3.79 (s, 3H), 3.84 (s, 6H), 7.28 (s, 2H), 7.69 (d, J = 8.4 Hz, 1H), 7.80 (d, J = 8.4 Hz, 1H), 8.12 (s, 1H), 9.32 (bs, 1H), 11.30 (bs, 1H). ^13^C NMR (101 MHz, DMSO-*d*_6_) δ 10.50, 56.55, 56.88 (2C), 107.94 (2C), 114.14, 115.82, 121.29, 126.47, 127.27, 129.64, 129.97, 132.71, 132.86, 142.66, 149.37, 153.11, 153.44 (2C), 163.08, 184.55. MS (ESI) *m*/*z* calcd. for C_22_H_19_NO_7_ [M + H]^+^: 410.12, found: 410.25. Anal. calcd for C_22_H_19_NO_7_. C, 64.54; H, 4.68; N, 3.42; found: C, 64.63; H, 4.82 N, 3.55

### 4.2. Biological Assays and Computational Studies

#### 4.2.1. Cell Cultures and Drug Screening

Colon adenocarcinoma (HT-29) cells were grown in RPMI-1640 medium, while non-small-cell lung carcinoma (A549), cervix carcinoma (HeLa), and breast adenocarcinoma (MDA-MB-231 and MCF-7) cells were grown in DMEM medium and supplemented with 115 units/mL of penicillin G, 115 μg/mL of streptomycin, and 10% foetal bovine serum (FBS, all purchased from Invitrogen, Milan, Italy). All the cell lines were purchased from the American Type Culture Collection (ATCC, Manassas, VA, USA). Drug stock solutions were prepared for each compound by dissolving them in DMSO at 10 mM.

Cancer cells were seeded in tissue treated, flat bottom, 384-well plates (Corning, Corning, NY, USA) according to their optimal density, in 27 μL of complete medium per well, using a Microlab STAR 96-CORE liquid handling system (Hamilton Bonaduz, Bonaduz, Switzerland) as described previously [75]. Each compound was tested in a 6-points 2-fold dose–response curve, and each dose was tested in duplicate within each plate, in three independent experiments. The final concentration of DMSO in the wells never exceeded 0.1%, which was the maximum concentration.

After 72 h of compound incubation, 3 μL of resazurin stock solution (10x) was added to each well for a final concentration of 44 μM, and the plates were incubated at 37 °C for an additional 3 h. The fluorescence signal was measured at 590 nm using a Spark 10M spectrophotometer (Tecan Group Ltd., Mannedorf, Switzerland). Only plates with acceptable Z-prime quality metrics (Z′ > 0.6) were further analyzed for the drug screening. Raw data were normalized according to the following equation: cell viability (%) = (*x* − POS)/(NEG − POS) × 100, where x is the relative fluorescence units (RFU) collected from each single well, NEG is the mean of intraplate negative controls (DMSO, corresponding to 100% cell viability), and POS is the mean of intraplate positive controls (Bortezomib 1 μΜ, equal to 0% cell viability). Normalized data were then processed with R 3.6.3 and Rstudio Version March 1 for GI_50_ calculation, defined as the compound concentration required to inhibit cell proliferation by 50% compared to DMSO.

#### 4.2.2. Effects on Tubulin Polymerization and on Colchicine Binding to Tubulin

Bovine brain tubulin was purified as described previously [76]. To evaluate the effects of the compounds on tubulin assembly in vitro [77], varying concentrations were preincubated with 10 μM tubulin in 0.8 M monosodium glutamate (pH 6.6) at 30 °C, and the reaction mixtures were then cooled to 0 °C. After addition of GTP, the mixtures were transferred to 0 °C cuvettes in Beckman Coulter (Brea, CA, USA) DU-7400/DU-7500 recording spectrophotometers equipped with electronic temperature controllers and were warmed to 30 °C, and the assembly of tubulin was observed turbidimetrically. The IC_50_ was defined as the compound concentration that inhibited the extent of assembly by 50% after a 20 min incubation. Inhibition of colchicine binding to tubulin was measured as described before [78], except that the reaction mixtures contained 0.5 μM tubulin and 5 μM each of [^3^H]colchicine and test compound. Only one DEAE-cellulose filter was used per sample, and filtration was via gravity.

#### 4.2.3. In Vitro HDAC Inhibition Assays

Screening of total HDAC inhibitory activity was performed using a Fluor de Lys^®^ HDAC inhibitor drug screening kit (Enzo Life Sciences, Milano, Italy) following the manufacturer’s instructions. Briefly, test compounds were incubated in a 96-well plate at two different concentrations (1 and 10 µM) with HeLa Nuclear extract for 3 h at 37 °C to measure the total HDAC inhibition. The fluorogenic developer FLUOR DE LYS^®^ was then added, and the fluorescence was read in a Spark 10M spectrophotometer (Tecan Group Ltd., Mannedorf, Switzerland) with a 360 nm excitation wavelength and a 460 nm emission wavelength.

Further experiments were also carried out to evaluate the inhibitory activities of test compounds on selected HDAC isoforms. Different concentrations of the compounds were incubated in a low-binding black 96-well plate with 30 ng of human recombinant HDAC6 (BPS Bioscience, San Diego, CA, USA; Cat. # 50056), human recombinant HDAC1 (BPS Bioscience; Cat. # 50051), human recombinant HDAC8 (BPS Bioscience; Cat. # 50008), or 500 ng of human recombinant HDAC10 (BPS Bioscience; Cat. # 50060) in an assay buffer containing 25 mM Tris/HCl, pH 8.0, 137 mM NaCl, 2.7 mM KCl, 1 mM MgCl_2_, and 0.1 mg/mL bovine serum albumin for 30 min at 37 °C. At the end of the incubation, the deacetylation reaction was initiated by adding 200 μM of the fluorogenic acetylated HDAC substrate 3 (BPS Bioscience; Cat. # 50037) for the HDAC6, HDAC1, and HDAC10 assays, or of the fluorogenic HDAC substrate class 2A (BPS Bioscience; Cat. # 50040) for the HDAC8 assays. After 30 min at 37 °C, the reaction was stopped by the addition of an HDAC assay developer (BPS Bioscience; Cat. # 50060). Following an incubation of 15 min at RT, fluorescence was measured in an EnSight multimodal plate reader (PerkinElmer, Boston, MA, USA) with an excitation wavelength of 360 nm and an emission wavelength of 450 nm.

#### 4.2.4. Molecular Modeling

All molecular docking studies were performed on a custom-made machine with Intel i9 12900Kx24 and NVIDIA RTX A5000 (assembled at UCT, Prague, Czech Republic, with components sourced in the Czech Republic), running Ubuntu 22.04. Molecular Operating Environment (MOE) 2022.02 [79] and Maestro (Schrodinger Release 2023-2) [80] were used as molecular modeling software. The structures were downloaded from the PDB data bank (http://www.rcsb.org/; Tubulin: PDB code 4O2B; hDAC6: PDB code 5EDU). The proteins were preprocessed using the Schrodinger Protein Preparation Wizard by assigning bond orders, adding hydrogens, and performing a restrained energy minimization of the added hydrogens using the OPLS_2005 force field. Ligand structures were built with MOE and then prepared using the Maestro LigPrep tool by energy minimizing the structures (OPLS_2005 force field), generating possible ionization states at pH 7 ± 2, and generating tautomers and low-energy ring conformers. A 12 A° docking grid (inner-box 10 A° and outer-box 22 A°) was prepared using as centroid the appropriate co-crystallized ligand. Molecular docking studies were performed using Glide SP precision, keeping the default parameters and setting 5 as the number of output poses per input ligand to include in the solution. The output database was saved as an sdf file. The docking results were visually inspected and evaluated in MOE for the ability of the compounds to bind into the active site.

#### 4.2.5. Cell Cycle Analysis

HT29 cells were treated with the indicated compounds for 24 h and then harvested and fixed in 70% ice-cold ethanol for 24 h. The cells were next treated with a 0.1% *v*/*v* solution of Triton X-100 in phosphate buffered saline (PBS) containing 10 μg/mL RNAse A and 20 μg/mL PI. The cells were incubated at room temperature for 30 min and then analyzed on a BD FACS Celesta^TM^ Flow Cytometer (BD Bioscience, Franklin Lakes, NJ, USA) in the FL3 channel. DNA histograms were analyzed using FlowJo_v10.7.1 software (BD Bioscience, Franklin Lakes, NJ, USA).

#### 4.2.6. Annexin V-PI Flow Cytometric Analysis

The quantification of apoptosis induced by the test compounds was carried out with flow cytometric analysis using the Annexin-V Fluos kit (Roche Diagnostics, Milan, Italy) following the manufacturer’s instructions. Cancer cells treated with the test compounds for the indicated time points and concentrations were then labeled with annexin V/FITC and PI and analyzed with BD FACS Celesta^TM^ Flow Cytometer (BD Bioscience, Franklin Lakes, NJ, USA) using the FL1 and FL3 channel, respectively, and FlowJo_v10.7.1 software.

#### 4.2.7. Western Blot Analysis

HT-29 cells were treated with selected compounds for 6 h at 10 µM, lysed, and then processed as previously described [81].

Antibodies directed against total histone H3 and its acetylated form (Ac-H3K9) were purchased from Cell Signaling Technology, Danvers, Massachusetts, while anti-vinculin antibody was purchased from Santa Cruz Biotechnology. Membranes were visualized using ECL select (GE Healthcare), and images were acquired using the iBright FL1500 Imaging System (Thermo Fisher Scientific, Waltham, MA, USA). Anti-vinculin (1:25,000, Santa Cruz Biotechnology, Dallas, TX, USA) was used as loading controls.

#### 4.2.8. Cytotoxicity Evaluation in Non-Tumoral Cells

Peripheral blood mononuclear cells (PBMC), obtained from human peripheral blood (leucocyte-rich plasma-buffy coats) of healthy volunteers using a Lymphoprep^TM^ (Serumwerk Bernburg AG) gradient, were used for the evaluation of the cytotoxic potential of compounds in normal human cells [82].

After extensive washing with saline solution (Hank’s Buffer Saline Solution, BioConcept, Allschwil, Switzerland), quiescent PBMCs were resuspended (1.0 × 10^6^ cells/mL) in RPMI-1640 medium supplemented with 10% FBS. To evaluate cytotoxicity in proliferating PBMCs cultures, cells were resuspended at a concentration of 5 × 10^5^ cells/mL in a growth medium containing 2.5 g/mL of PHA (Irvine Scientific, Santa Ana, CA, USA). The same cellular density was utilized for resting PBMC cultures, without the addition of PHA. The tested compounds were added at different concentrations, and after a 72 h incubation, cell viability was determined using the resazurin test as described above.

#### 4.2.9. Statistical Analysis

All statistical analyses were performed using GraphPad Prism 10 (GraphPad, La Jolla, CA, USA). The data presented in bar graphs are represented as mean ± SEM. Statistical comparisons among three or more experimental groups were performed using one-way ANOVA followed by Holm–Sidak post-test multiple comparison. Statistical significance is indicated by asterisks placed above the bars to denote a significant difference compared to control cells or specific experimental groups (indicated within brackets, if applicable). The significance levels were defined as follows: * *p* < 0.05, ** *p* < 0.01, *** *p* < 0.001, **** *p* < 0.0001.

## 5. Conclusions

In conclusion, for the most active compounds **6a**, **6c**, **6e**, **6g**, **11a**, and **11c**, a good correlation was observed between their antiproliferative and antitubulin activities. Compounds with substantially lower antiproliferative effects on cancer cells had much lower inhibitory activities on tubulin assembly. The potent antiproliferative activity shown by compounds **6e**, **6g**, and **11c** indicated that the trimethoxyphenyl group on the 2-benzoyl moiety is not a fundamental requirement for achieving activity, while the 6-OMe group is essential for potent cytoxicity. Further studies also suggested that antiproliferative activity was more correlated with tubulin polymerization inhibition than HDAC inhibition.

## Figures and Tables

**Table 2:** Inhibition of tubulin polymerization and colchicine binding by compounds **6a**–**f**, **6i**, **11a**–**c**, **11e**–**f**, **TR187**, and CA-4.

Compounds	Tubulin Assembly ^a^ IC_50_ ± SD (µM)	Colchicine Binding ^b^ % Inhibition ± SD
**6a**	0.58 ± 0.06	69 ± 2
**6b**	1.10 ± 0.20	41 ± 5
**6c**	0.42 ± 0.07	81 ± 4
**6d**	1.70 ± 0.10	27 ± 4
**6e**	1.10 ± 0.10	86 ± 1
**6f**	1.20 ± 0.10	76 ± 2
**6g**	0.69 ± 0.10	86 ± 5
**6i**	1.60 ± 0.30	43 ± 4
**11a**	0.49 ± 0.10	69 ± 0.1
**11b**	2.70 ± 0.07	30 ± 2
**11c**	0.56 ± 0.06	82 ± 1
**11e**	0.42 ± 0.03	81 ± 3
**11f**	5.40 ± 0.10	20 ± 2
**CA-4**	0.75 ± 0.06	98 ± 2

^a^ Inhibition of tubulin polymerization. Tubulin was at 10 μM. ^b^ Inhibition of [^3^H]colchicine binding. Tubulin, [^3^H]colchicine, and tested compound were at 0.5, 5, and 5 µM concentrations, respectively.

**Table 3:** Inhibitory activity of compounds **6a**–**g**, **6i**, **11a**, **11c**, **11c**, and panobinostat against HDAC6, 1, 8, and 10.

Compound	IC_50_ (µM)			
	*h*HDAC6	*h*HDAC1	*h*HDAC8	*h*HDAC10
**6a**	6.00 ± 0.39	6.59 ± 0.42	>30	4.38 ± 0.32
**6b**	>30	-	-	-
**6c**	12.5 ± 0.8	>30	>30	>30
**6d**	12.6 ± 0.8	-	-	-
**6e**	17.1 ± 1.2	13.4 ± 0.9	>30	>30
**6f**	6.12 ± 0.41	-	-	-
**6g**	10.2 ± 0.7	-	-	-
**6i**	5.16 ± 0.34	-	-	-
**11a**	>30	14.1 ± 1.0	>30	>30
**11c**	13.8 ± 0.8	-	-	-
**11e**	>30	-	-	-
Panobinostat	0.0092 ± 0.0006	0.058 ± 0.004	0.274 ± 0.022	0.0056 ± 0.0004

Data are expressed as the mean ± SE of at least three independent experiments.

**Table 4:** Cytotoxicity of compounds **6f**, **6g**, and **6i**, in human peripheral blood mononuclear cells (PBMC).

Cell Line	GI_50_ (µM) ^a^		
	6f	6g	6i
PBMC_resting_ ^b^	>10	>10	>10
PBMC_PHA_ ^c^	0.51 ± 0.10	0.15 ± 0.02	2.32 ± 0.23

^a^ Compound concentration required to reduce cell growth inhibition by 50%. ^b^ PBMC not stimulated with PHA. ^c^ PBMC stimulated with PHA. Values are the mean ± SEM for three separate experiments.

## Data Availability

Data supporting the reported results are available on request from the corresponding authors.

## Supplementary Materials

The following supporting information can be downloaded at: https://www.mdpi.com/article/10.3390/ijms25147519/s1.

## References

[1] Logan C.M., Menko A.S. (2019). Microtubules: Evolving roles and critical cellular interactions. Exp. Biol. Med..

[2] Ilan Y. (2022). Microtubules as a potential platform for energy transfer in biological systems: A target for implementing individualized, dynamic variability patterns to improve organ function. Mol. Cell. Biochem..

[3] Wordeman L., Vicente J.J. (2021). Microtubule targeting agents in disease: Classic drugs, novel roles. Cancers.

[4] Eli S., Castagna R., Mapelli M., Parisini E. (2022). Recent approaches to the identification of novel microtubule-targeting agents. Front. Mol. Biosci..

[5] Henriques A.C., Ribeiro D., Pedrosa J., Sarmento B., Silva P.M.A., Bousbaa H. (2019). Mitosis inhibitors in anticancer therapy: When blocking the exit becomes a solution. Cancer Lett..

[6] Muhlethaler T., Gioia D., Prota A.E., Sharpe M.E., Cavalli A., Steinmetz M.O. (2021). Comprehensive analysis of binding sites in tubulin. Angew. Chem. Int. Ed. Engl..

[7] Yang C.-H., Horwitz S.B. (2017). Taxol^®^: The first microtubule stabilizing agent. Int. J. Mol. Sci..

[8] Prota A.E., Bargsten K., Northcote P.T., Marsh M., Altmann K.H., Miller J.H., Diaz J.F., Steinmetz M.O. (2014). Structural basis of microtubule stabilization by laulimalide and peloruside A. Angew. Chem. Int. Ed..

[9] Field J.J., Diaz J.F., Miller J.H. (2013). The binding sites of microtubule-stabilizing agents. Chem. Biol..

[10] Ravelli R.B., Gigant B., Curmi P.A., Jourdain I., Lachkar S., Sobel A., Knossow M. (2004). Insight into tubulin regulation from a complex with colchicine and a stathmin-like domain. Nature.

[11] Risinger A.L., Du L. (2020). Targeting and extending the eukaryotic druggable genome with natural products: Cytoskeletal targets of natural products. Nat. Prod. Rep..

[12] Liang T., Lu L., Song X., Qi J., Wang J. (2022). Combination of microtubule targeting agents with other antineoplastics for cancer treatment. BBA-Rev. Cancer.

[13] Krause W. (2019). Resistance to anti-tubulin agents: From vinca alkaloids to epothilones. Cancer Drug Resist..

[14] Kanakkanthara A., Miller J.H. (2021). βIII-tubulin overexpression in cancer: Causes, consequences, and potential therapies. Biochim. Biophys. Acta Rev. Cancer.

[15] Ling X., Bernacki R.J., Brattain M.G., Li F. (2004). Induction of survivin expression by taxol (paclitaxel) is an early event, which is independent of taxol-mediated G2/M arrest. J. Biol. Chem..

[16] Kamal M.A., Al-Zahrani M.H., Khan S.H., Khan M.H., Al-Subhi H.A., Kuerban A., Aslam M., Al-Abbasi F.A., Anwar F. (2020). Tubulin proteins in cancer resistance: A review. Curr. Drug Metabol..

[17] Canta A., Chiorazzi A., Cavaletti G. (2009). Tubulin: A target for antineoplastic drugs into the cancer cells but also in the peripheral nervous system. Curr. Med. Chem..

[18] McLoughlin E.C., O’Boyle N.M. (2020). Colchicine-binding site inhibitors from chemistry to clinic: A review. Pharmaceuticals.

[19] Hawash M. (2022). Recent advances of tubulin inhibitors targeting the colchicine binding site for cancer therapy. Biomolecules.

[20] Dong M., Liu F., Zhou H., Zhai S., Yan B. (2016). Novel natural product- and privileged scaffold-based tubulin inhibitors targeting the colchicine binding site. Molecules.

[21] Zhang Y., Li B., Yan R., Xia L., Fan A., Chu Y., Wang L., Wang Z., Jiang A., Zhu H. (2019). A class of novel tubulin polymerization inhibitors exert effective antitumor activity via mitotic catastrophe. Eur. J. Med. Chem..

[22] Lin C.M., Ho H.H., Pettit G.R., Hamel E. (1989). Antimitotic natural products combretastatin A-4 and combretastatin A-2: Studies on the mechanism of their inhibition of the binding of colchicine to tubulin. Biochemistry.

[23] Griggs J., Metcalfe J.C., Hesketh R. (2001). Targeting tumour vasculature: The development of combretastatin A4. Lancet Oncol..

[24] Nagaiah G., Remick S.C. (2010). Combretastatin A4 phosphate: A novel vascular disrupting agent. Future Oncol..

[25] Shiah H.-S., Chiang N.-J., Lin C.-C., Yen C.-J., Tsai H.-J., Wu S.-Y., Su W.-C., Chang K.-Y., Wang C.C., Chang J.-Y. (2021). Phase I dose-escalation study of SCB01A, a microtubule inhibitor with vascular disrupting activity, in patients with advanced Solid Tumors. Oncologist.

[26] Rischin D., Bibby D.C., Chong G., Kremmidiotis G., Leske A.F., Matthews C.A., Wong S.S., Rosen M.A., Desai J. (2011). Clinical, pharmacodynamic, and pharmacokinetic evaluation of BNC105P: A Phase I trial of a novel vascular disrupting agent and inhibitor of cancer cell proliferation. Clin. Cancer Res..

[27] Delmonte A., Sessa C. (2009). AVE8062: A new combretastatin derivative vascular disrupting agent. Exp. Opin. Investig. Drugs.

[28] Markowski M.C., Tutrone R., Pieczonka C., Barnette K.G., Getzenberg R.H., Rodriguez D., Steiner M.S., Saltzstein D.R., Eisenberger M.A., Antonarakis E.S. (2022). A Phase Ib/II study of Sabizabulin, a novel oral cytoskeleton disruptor, in men with metastatic castration-resistant prostate cancer with progression on an androgen receptor-targeting agent. Clin. Cancer Res..

[29] Niu L., Yang J., Yan W., Yu Y., Zheng Y., Ye H., Chen X.Q., Chen L. (2019). Reversible binding of the anticancer drug KXO1(tirbanibulin) to the colchicine-binding site of tubulin explains KXO1’s low clinical toxicity. J. Biol. Chem..

[30] Blauvelt A., Kempers S., Lain E., Schlesinger T., Tyring S., Forman S., Ablon G., Martin G., Wang H., Cutler D.L. (2021). Phase 3 trials of tirbanibulin ointment for actinic keratosis. N. Engl. J. Med..

[31] Jones P.A., Issa J.-P.J., Baylin S. (2016). Targeting the cancer epigenome for therapy. Nat. Rev. Genet..

[32] Thiagalingam S., Cheng K.H., Lee H.J., Mineva N., Thiagalingam A., Ponte J.F. (2003). Histone deacetylases: Unique players in shaping the epigenetic histone code. Ann. N. Y. Acad. Sci..

[33] Milazzo G., Mercatelli D., Di Muzio G., Triboli L., De Rosa P., Perini G., Giorgi F.M. (2020). Histone deacetylases (HDACs): Evolution, specificity, role in transcriptional complexes, and pharmacological actionability. Genes.

[34] Haberland M., Montgomery R.L., Olson E.N. (2009). The many roles of histone deacetylases in development and physiology: Implications for disease and therapy. Nat. Rev. Genet..

[35] Glozak M.A., Sengupta N., Zhang X., Seto E. (2005). Acetylation and deacetylation of non-histone proteins. Gene.

[36] Eckschlager T., Plch J., Stiborova M., Hrabeta J. (2017). Histone deacetylase inhibitors as anticancer drugs. Int. J. Mol. Sci..

[37] Ruijter A.J., Gennip A.H., Caron H.N., Kemp S., Kuilenburg A.B.P. (2003). Histone deacetylases (HDACs) characterization of the classical HDAC family. Biochem. J..

[38] Xu W.S., Parmigiani R.B., Marks P.A. (2007). Histone deacetylase inhibitors: Molecular mechanisms of action. Oncogene.

[39] Marks P.A. (2007). Discovery and development of SAHA as an anticancer agent. Oncogene.

[40] Rashidi A., Cashen A.F. (2015). Belinostat for the treatment of relapsed or refractory peripheral T-cell lymphoma. Future Oncol..

[41] Bertino E.M., Otterson G.A. (2011). Romidepsin: A novel histone deacetylase inhibitor for cancer. Expert. Opin. Investig. Drugs.

[42] Libby E.N., Becker P.S., Burwick N., Green D.J., Holmberg L., Bensinger W.I. (2015). Panobinostat: A review of trial results and future prospects in multiple myeloma. Expert Rev. Hematol..

[43] Shi Y., Jia B., Xu W., Li W., Liu T., Liu P., Zhao W., Zhang H., Sun X., Yang H. (2017). Chidamide in relapsed or refractory peripheral T cell lymphoma: A multicenter real-world study in China. J. Hematol. Oncol..

[44] Schiattarella G.G., Sannino A., Toscano E., Cattaneo F., Trimarco B., Esposito G., Perrino C. (2016). Cardiovascular effects of histone deacetylase inhibitors epigenetic therapies: Systematic review of 62 studies and new hypotheses for future research. Int. J. Cardiol..

[45] Halsall J.A., Turner B.M. (2016). Histone deacetylase inhibitors for cancer therapy: An evolutionarily ancient resistance response may explain their limited success. BioEssays News Rev. Mol. Cell. Dev. Biol..

[46] Amnekar R., Gupta S. (2018). HDAC inhibitors in solid tumors: An incomplete story. J. Clin. Epigenet..

[47] Jenke R., Reßing N., Hansen F.K., Aigner A., Büch T. (2021). Anticancer therapy with HDAC inhibitors: Mechanism-based combination strategies and future perspectives. Cancers.

[48] Morel D., Jeffery D., Aspeslagh S., Almouzni G., Postel-Vinay S. (2020). Combining epigenetic drugs with other therapies for solid tumours-past lessons and future promise. Nat. Rev. Clin. Oncol..

[49] Hubbert C., Guardiola A., Shao R., Kawaguchi Y., Ito A., Nixon A., Yoshida M., Wang X.F., Yao T.P. (2002). HDAC6 is a microtubule-associated deacetylase. Nature.

[50] Chao M.W., Lai M.J., Liou J.P., Chang Y.L., Wang J.C., Pan S.L., Teng C.M. (2015). The synergic effect of vincristine and vorinostat in leukemia in vitro and in vivo. J. Hematol. Oncol..

[51] Zuco V., De Cesare M., Cincinelli R., Nannei R., Pisano C., Zaffaroni N., Zunino F. (2011). Synergistic antitumor effects of novel HDAC inhibitors and paclitaxel in vitro and in vivo. PLoS ONE.

[52] Yoo J., Jeon Y.H., Lee D.H., Kim G.W., Lee S.W., Kim S.Y., Park J., Kwon S.H. (2021). HDAC6-selective inhibitors enhance anticancer effects of paclitaxel in ovarian cancer cells. Oncol. Lett..

[53] Anighoro A., Bajorath J., Rastelli G. (2014). Polypharmacology: Challenges and opportunities in drug discovery. J. Med. Chem..

[54] Bass A.K.A., El-Zoghbi M.S., Nageeb E.S.M., Mohamed M.F.A., Badr M., Abuo-Rahma G.E.D.A. (2021). Comprehensive review for anticancer hybridized multitargeting HDAC inhibitors. Eur. J. Med. Chem..

[55] Liu T., Wan Y., Xiao Y., Xia C., Duan G. (2020). Dual-target inhibitors based on HDACs: Novel antitumor agents for cancer therapy. J. Med. Chem..

[56] Beljkas M., Ilic A., Cebzan A., Radovic B., Djokovic N., Ruzic D., Nikolic K., Oljacic S. (2023). Targeting histone deacetylases 6 in dual-target therapy of cancer. Pharmaceutics.

[57] Brunetti M., Renzoni D., Chakravarty P., Paolini C., De Francesco R., Gallinari P., Steinkühler C., Di Marco S. (2004). Crystal structure of a eukaryotic zinc-dependent histone deacetylase, human HDAC8, complexed with a hydroxamic acid inhibitor. Proc. Natl. Acad. Sci. USA.

[58] Li Y., Wang F., Chen X., Wang J., Zhao Y., Li Y., He B. (2019). Zinc-dependent deacetylase (HDAC) inhibitors with different zinc binding groups. Curr. Top. Med. Chem..

[59] Zhang L., Zhang J., Jiang Q., Zhang L., Song W. (2018). Zinc binding groups for histone deacetylase inhibitors. J. Enzyme Inhib. Med. Chem..

[60] Shirbhate E., Singh V., Jahoriya V., Mishra A., Veerasamy R., Tiwari A.K., Rajak H. (2024). Dual inhibitors of HDAC and other epigenetic regulators: A novel strategy for cancer treatment. Eur. J. Med. Chem..

[61] Zagni C., Floresta G., Monciino G., Rescifina A. (2017). The search for potent, small-molecule HDACIs in cancer treatment: A decade after vorinostat. Med. Res. Rev..

[62] Wang B., Chen X., Gao J., Su L., Zhang L., Xu H., Luan Y. (2019). Anti-tumor activity evaluation of novel tubulin and HDAC dual-targeting inhibitors. Bioorg. Med. Chem. Lett..

[63] Romagnoli R., Baraldi P.G., Carrion M.D., Cara C.L., Cruz-Lopez O., Tolomeo M., Grimaudo S., Cristina A.D., Pipitone M.R., Balzarini J. (2009). Design, synthesis and structure–activity relationship of 2-(3′,4′,5′- trimethoxybenzoyl)-benzo[*b*]furan derivatives as a novel class of inhibitors of tubulin polymerization. Bioorg. Med. Chem..

[64] Kamal A., Reddy N.V., Nayak V.L., Reddy V.S., Prasad B., Nimbarte V.D., Srinivasulu V., Vishnuvardhan M.V., Reddy C.S. (2014). Synthesis and biological evaluation of benzo[b]furans as inhibitors of tubulin polymerization and inducers of apoptosis. ChemMedChem.

[65] Romagnoli R., Baraldi P.G., Lopez-Cara C., Cruz-Lopez O., Carrion M.D., Kimatrai Salvador M., Bermejo J., Estévez S., Estévez F., Balzarini J. (2011). Synthesis and antitumor molecular mechanism of agents based on amino 2-(3′,4′,5′-trimethoxybenzoyl)benzo[*b*]furan: Inhibition of tubulin and induction of apoptosis. ChemMedChem.

[66] Peng X., Sun Z., Kuang P., Chen J. (2020). Recent progress on HDAC inhibitors with dual targeting capabilities for cancer treatment. Eur. J. Med. Chem..

[67] Vaidya G.N., Rana P., Venkatesh A., Chatterjee D.R., Contractor D., Satpute D.P., Nagpure M., Jain A., Kumar D. (2021). Paradigm shift of "classical" HDAC inhibitors to “hybrid” HDAC inhibitors in therapeutic interventions. Eur. J. Med. Chem..

[68] Shuai W., Wang G., Zhang Y., Bu F., Zhang S., Miller D.D., Li W., Ouyang L., Wang Y. (2021). Recent progress on tubulin inhibitors with dual targeting capabilities for cancer therapy. J. Med. Chem..

[69] Li L., Jiang S., Li X., Liu Y., Su J., Chen J. (2018). Recent advances in trimethoxyphenyl (TMP) based tubulin inhibitors targeting the colchicine binding site. Eur. J. Med. Chem..

[70] Schobert R., Effenberger-Neidnicht K., Biersack B. (2011). Stable combretastatin A-4 analogues with sub-nanomolar efficacy against chemoresistant HT-29 cells. Int. J. Clin. Pharmacol. Ther..

[71] Malebari A.M., Greene L.M., Nathwani S.M., Fayne D., O’Boyle N.M., Wang S., Twamley B., Zisterer D.M., Meegan M.J. (2017). β-Lactam analogues of combretastatin A-4 prevent metabolic inactivation by glucuronidation in chemoresistant HT-29 colon cancer cells. Eur. J. Med. Chem..

[72] Tummatorn J., Ruchirawat S., Ploypradith P. (2010). A convergent general strategy for the functionalized 2-aryl cycloalkyl-fused chromans: Intramolecular hetero-Diels-Alder reactions of ortho-quinone methides. Chemistry.

[73] Ansari M., Shokrzadeh M., Karima S., Rajaei S., Fallah M., Ghassemi-Barghi N., Ghasemian M., Emami S. (2020). New thiazole-2(3*H*)-thiones containing 4-(3,4,5-trimethoxyphenyl) moiety as anticancer agents. Eur. J. Med. Chem..

[74] Lindgren A.E.G., Öberg C.T., Hillgren J.M., Elofsson M. (2016). Total synthesis of the resveratrol oligomers (±)-ampelopsin B and (±)-ϵ-viniferin. Eur. J. Org. Chem..

[75] Budassi F., Marchioro C., Canton M., Favaro A., Sturlese M., Urbinati C., Rusnati M., Romagnoli R., Viola G., Mariotto E. (2023). Design, synthesis and biological evaluation of novel 2,4-thiazolidinedione derivatives able to target the human BAG3 protein. Eur. J. Med. Chem..

[76] Hamel E., Lin C.M. (1984). Separation of active tubulin and microtubule-associated proteins by ultracentrifugation and isolation of a component causing the formation of microtubule bundles. Biochemistry.

[77] Hamel E. (2003). Evaluation of antimitotic agents by quantitative comparisons of their effects on the polymerization of purified tubulin. Cell Biochem. Biophys..

[78] Verdier-Pinard P., Lai J.Y., Yoo H.D., Yu J., Marquez B., Nagle D.G., Nambu M., White J.D., Falck J.R., Gerwick W.H. (1998). Structure-activity analysis of the interaction of curacin A, the potent colchicine site antimitotic agent, with tubulin and effects of analogs on the growth of MCF-7 breast cancer cells. Mol. Pharmacol..

[79] ULC, Chemical Computing Group. Molecular Operating Environment (MOE). 2022.02. https://www.chemcomp.com/Products.htm.

[80] Schrodinger Release 2023-2: Maestro, Schrodinger, LLC, New York, NY, 2019. https://www.schrodinger.com/maestro.

[81] Castro-Navas F.F., Schiaffino-Ortega S., Carrasco-Jimenez M.P., Ríos-Marco P., Marco C., Espinosa A., Gallo M.A., Mariotto E., Basso G., Viola G. (2015). New more polar symmetrical bipyridinic compounds: New strategy for the inhibition of choline kinase α1. Future Med. Chem..

[82] Romagnoli R., Baraldi P.G., Prencipe F., Oliva P., Baraldi S., Salvador M.K., Lopez-Cara L.C., Brancale A., Ferla S., Hamel E. (2017). Synthesis and biological evaluation of 2-methyl-4,5-disubstituted oxazoles as a novel class of highly potent antitubulin agents. Sci. Rep..

