# Supplementary material for: Synthesis and Biological Evaluation of Novel 2-Aroyl Benzofuran-Based Hydroxamic Acids as Antimicrotubule Agents
Source: Int J Mol Sci. 2024 Jul 9;25(14):7519. doi: 10.3390/ijms25147519 (PMC11277476; doi:10.3390/ijms25147519)
Supplement: Supplementary file 1 [file ijms-25-07519-s001.zip › ijms-3078126-supplementary.pdf]

Synthesis and Biological Evaluation of Novel 2-Aroyl Benzofuran-Based Hydroxamic Acids as Antimicrotubule Agents

Elena Mariotto, Giampietro Viola, Chiara Padroni, Andrea Brancale, Ernest Hamel, Fabrizio Vincenzi, Katia Varani, Tiziano De Ventura and Romeo Romagnoli*

Supplementary data

^{1}H-NMR spectra of compound **6a** 3

^{13}C-NMR spectra of compound **6a** 4

^{1}H-NMR spectra of compound **6b** 5

^{13}C-NMR spectra of compound **6b** 6

^{1}H-NMR spectra of compound **6c** 7

^{13}C-NMR spectra of compound **6c** 8

^{1}H-NMR pectra of compound **6d** 9

^{13}C-NMR spectra of compound **6d** 10

^{1}H-NMR spectra of compound **6e** 11

^{13}C-NMR spectra of compound **6e** 12

^{1}H-NMR spectra of compound **6f** 13

^{13}C-NMR spectra of compound **6f** 14

^{1}H-NMR spectra of compound **6g** 15

^{13}C-NMR spectra of compound **6g** 16

^{1}H-NMR spectra of compound **6h** 17

^{13}C-NMR spectra of compound **6h** 18

^{1}H-NMR spectra of compound **6i** 19

^{13}C-NMR spectra of compound **6i** 20

^{1}H-NMR spectra of compound **11a** 21

^{13}C-NMR spectra of compound **11a** 22

^{1}H-NMR spectra of compound **11b** 23

^{13}C-NMR spectra of compound **11b** 24

^{1}H-NMR spectra of compound **11c** 25

^{13}C-NMR spectra of compound **11c** 26

^{1}H-NMR spectra of compound **11d** 27

^{13}C-NMR spectra of compound **11d** 28

^{1}H-NMR spectra of compound **11e** 29

^{13}C-NMR spectra of compound **11e** 30

^{1}H-NMR spectra of compound **11f** 31

^{13}C-NMR spectra of compound **11f** 32

^{1}H-NMR spectra of compound **11g** 33

^{13}C-NMR spectra of compound **11g** 34

^{1}H-NMR spectra of compound **11h** 35

^{13}C-NMR spectra of compound **11h** 36

^{1}H-NMR spectra of compound **8** 37

^{13}C-NMR spectra of compound **8** 38

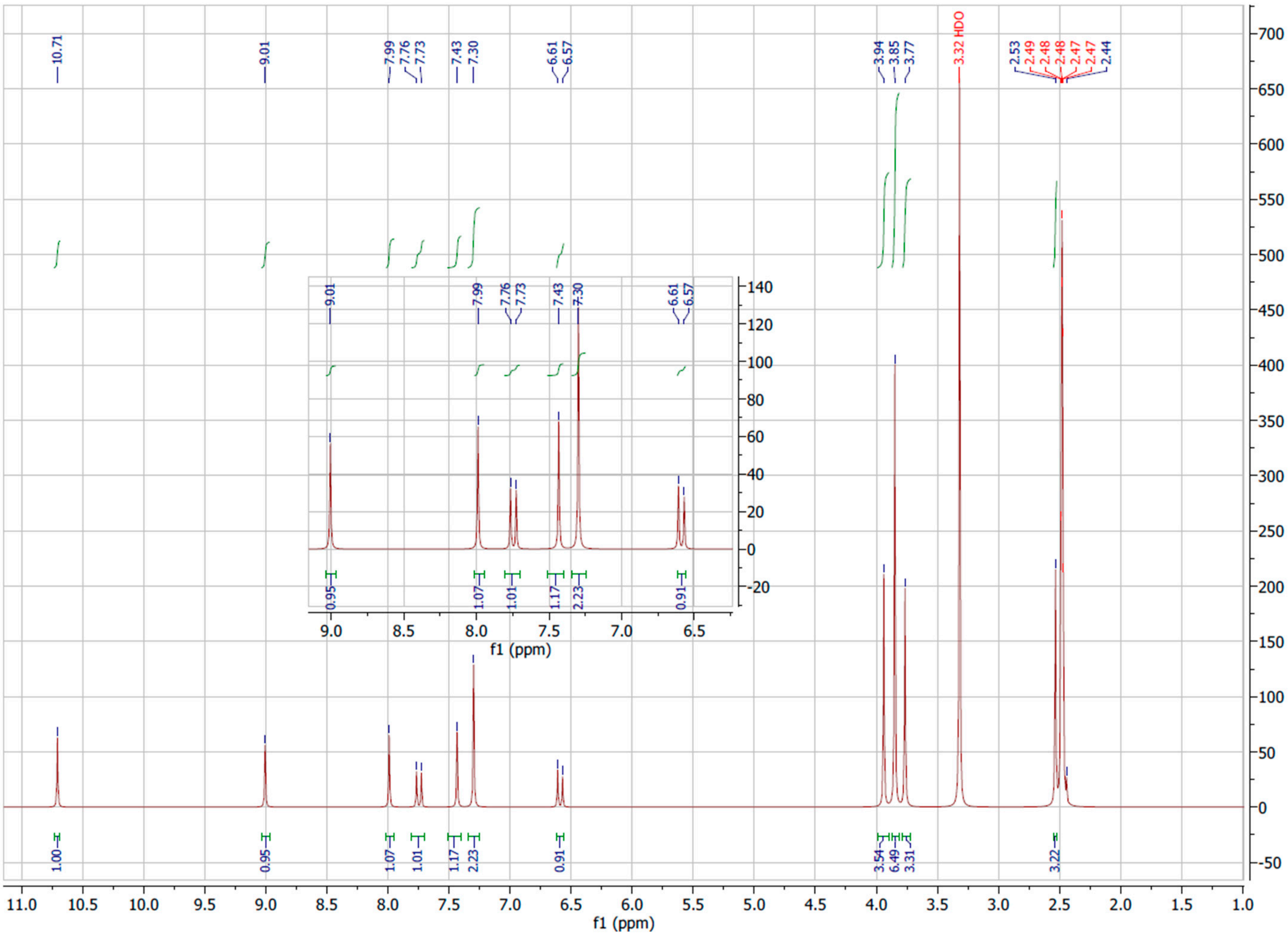

^{1}H-NMR spectra of compound **6a**

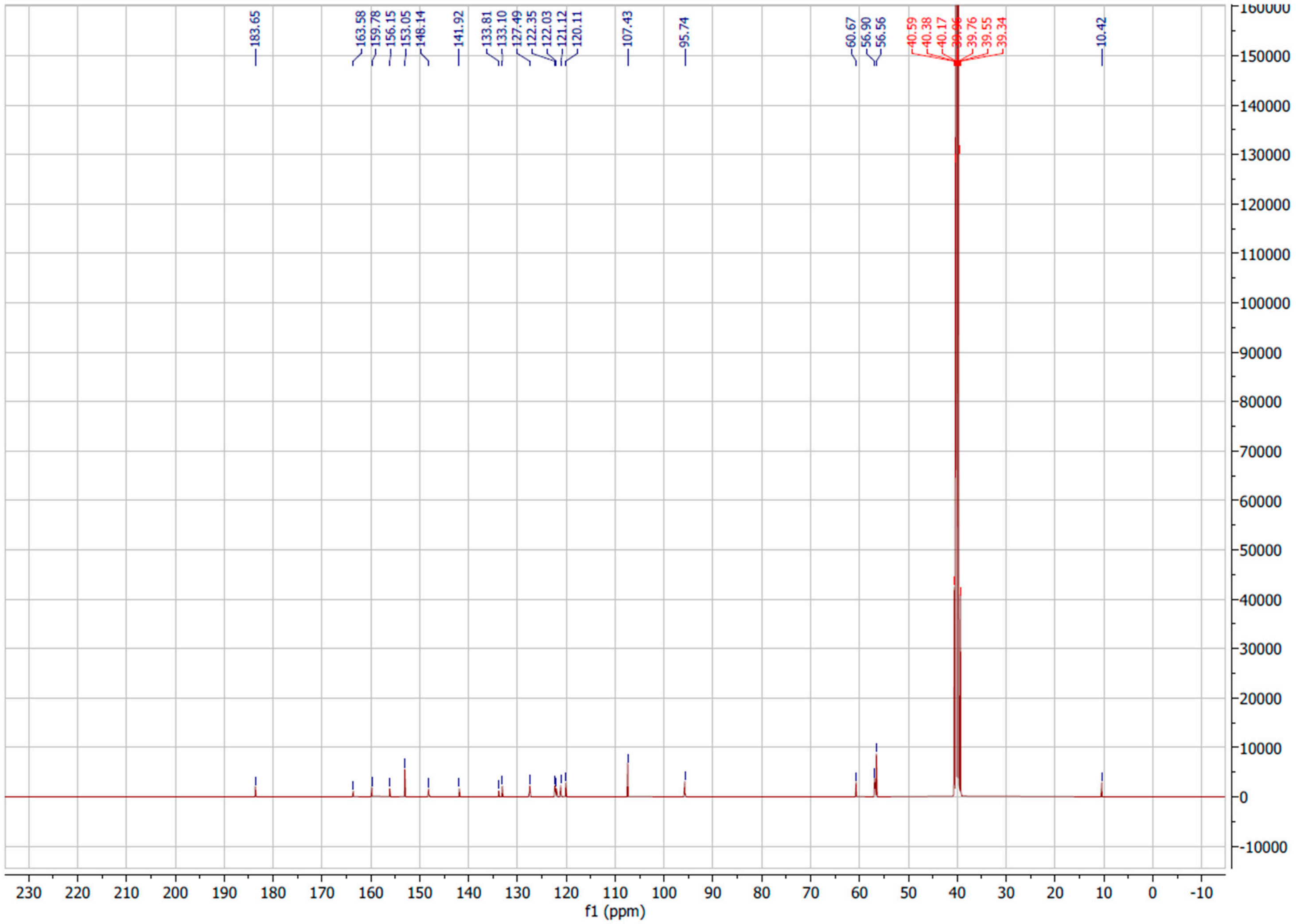

^{13}C-NMR spectra of compound **6a**

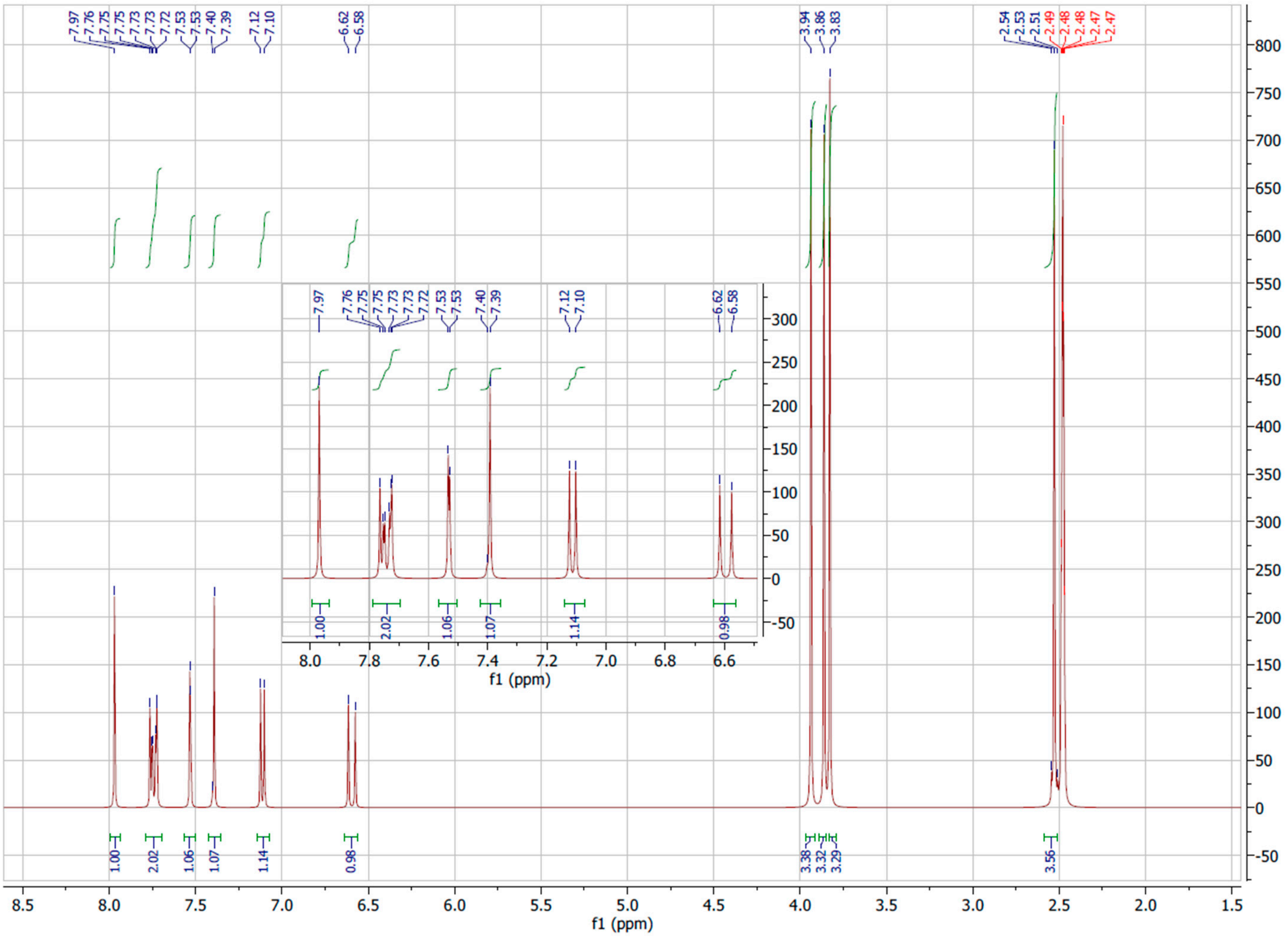

^{1}H-NMR of compound **6b**

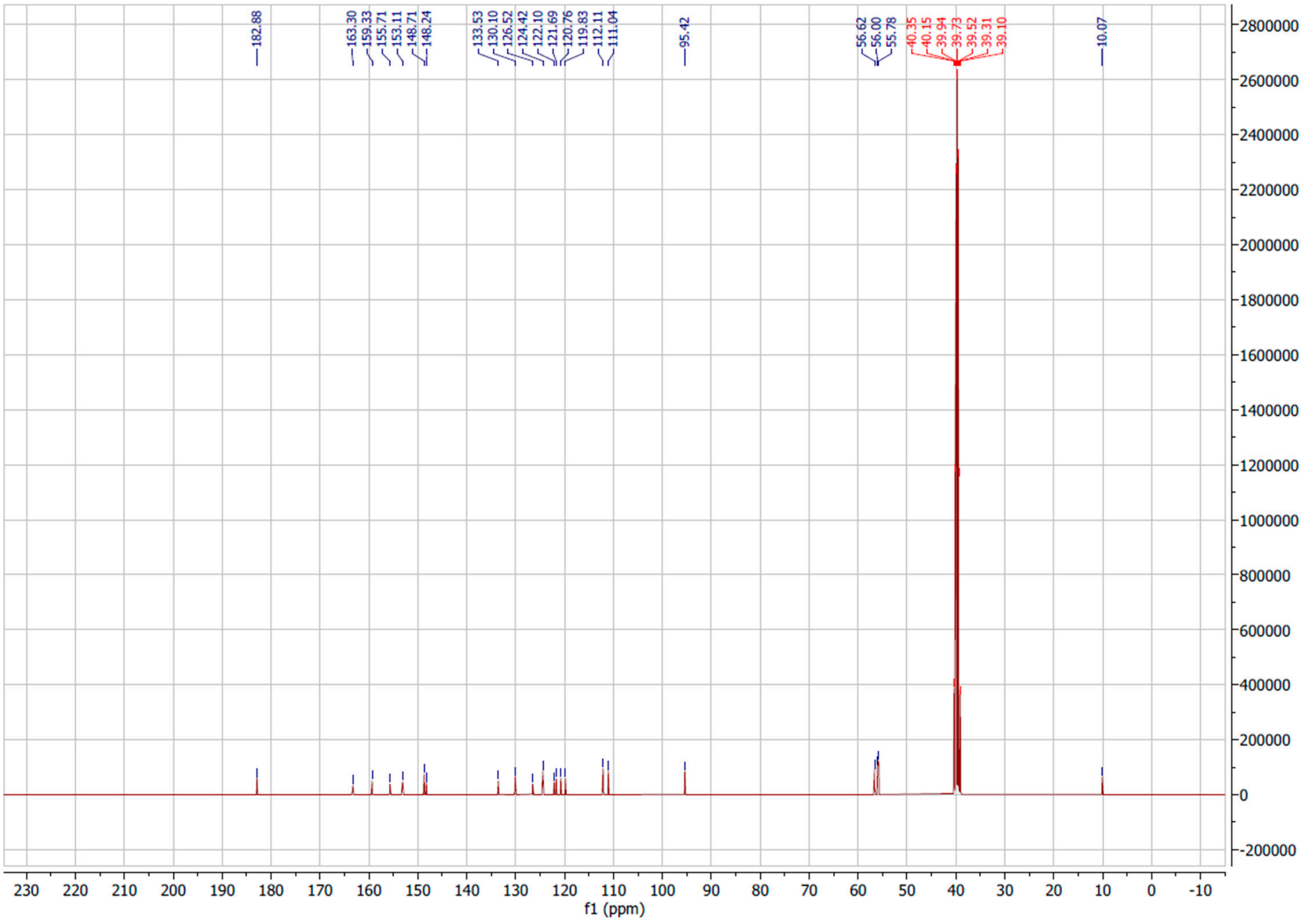

^{13}C-NMR spectra of compound **6b**

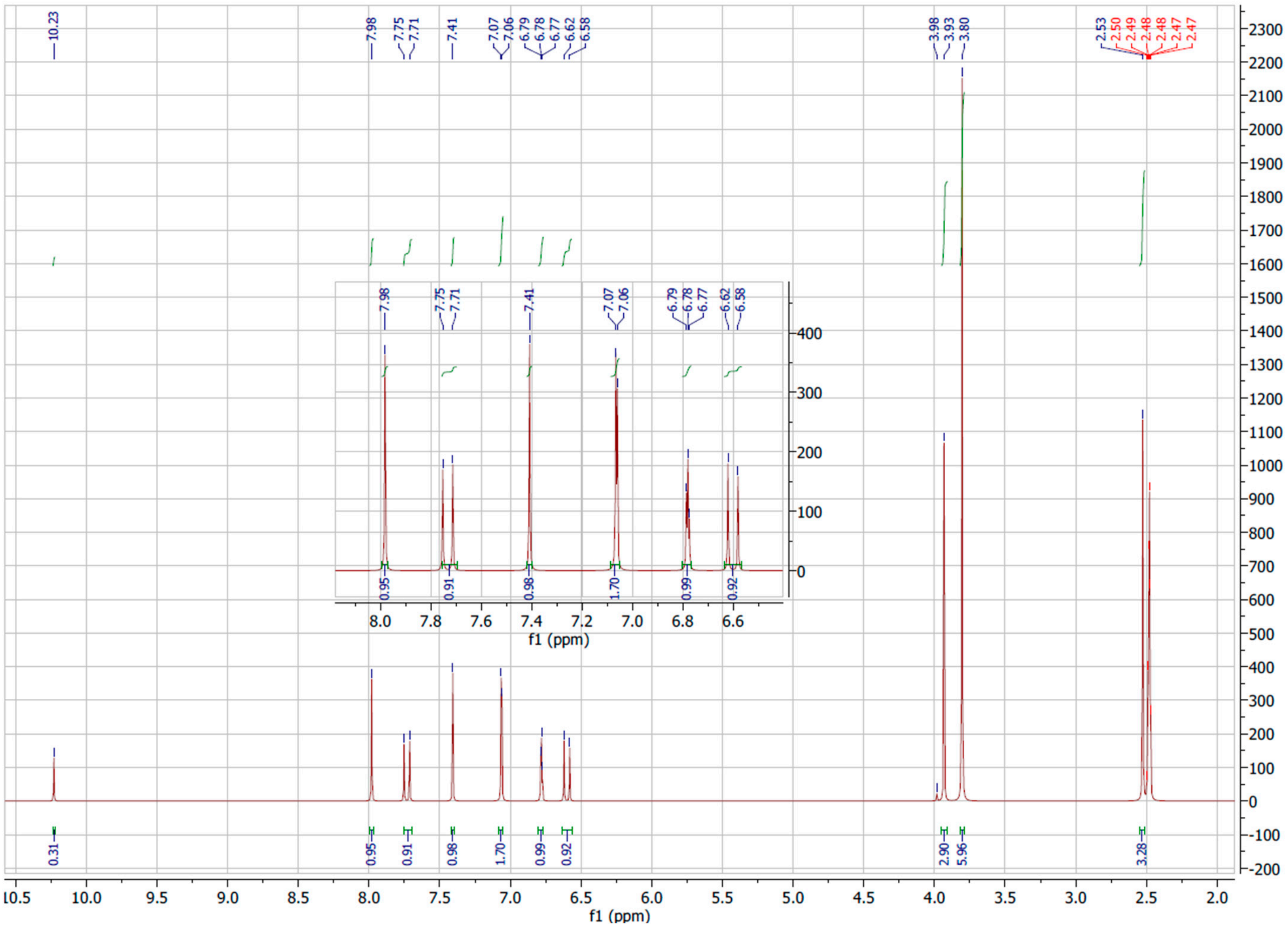

^{1}H-NMR spectra of compound **6c**

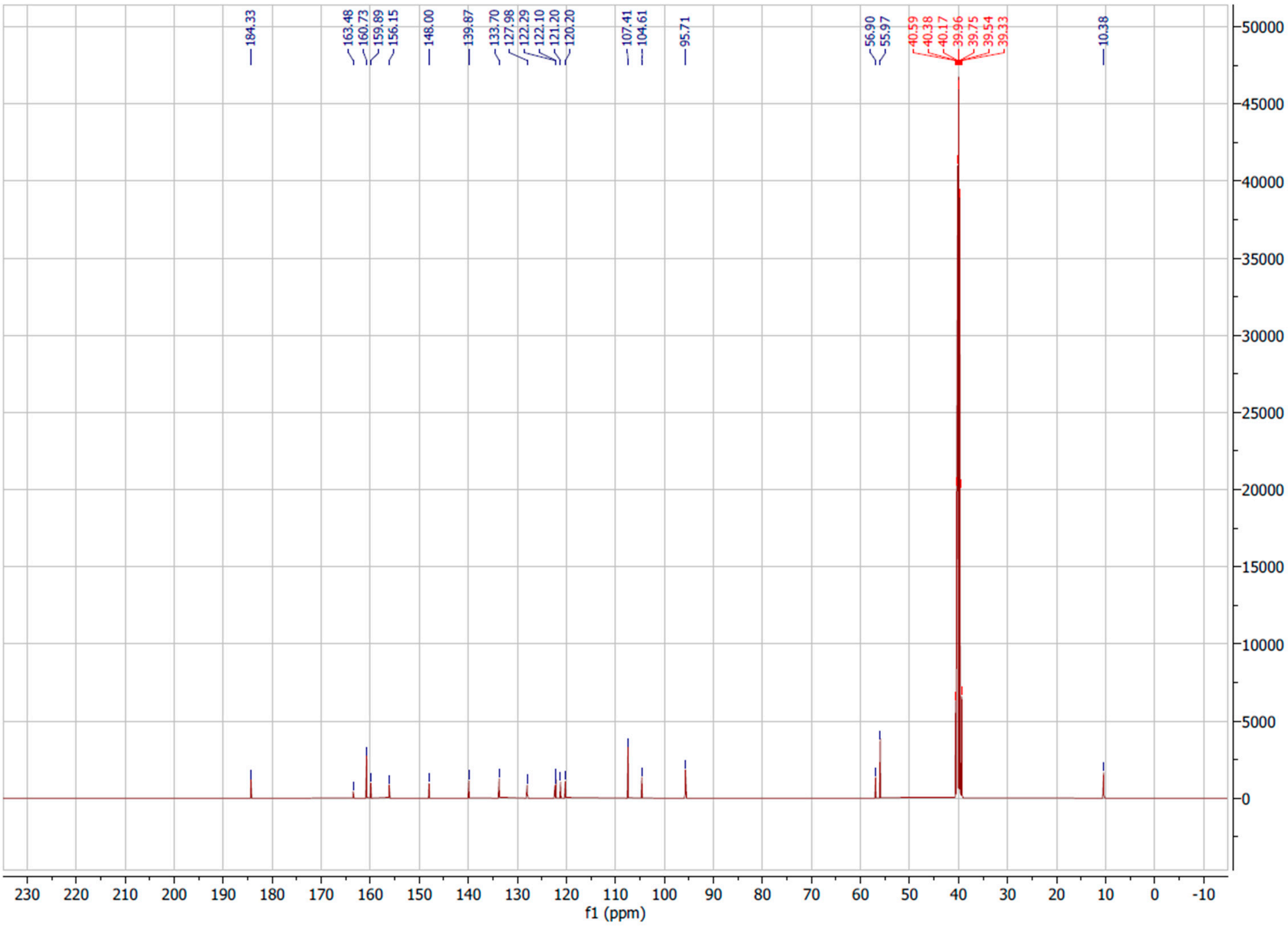

^{13}C-NMR spectra of compound **6c**

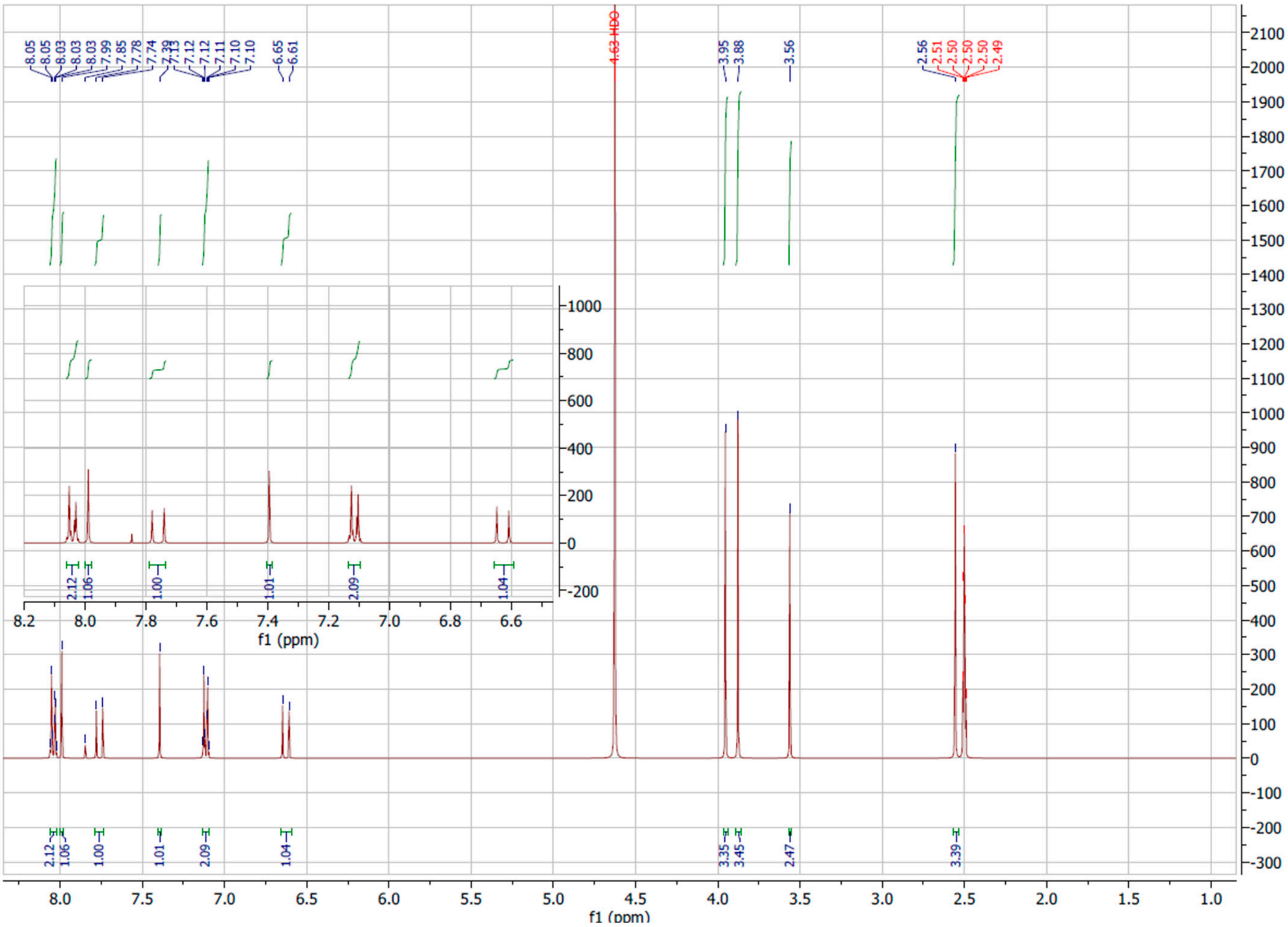

^{1}H-NMR spectra of compound **6d**

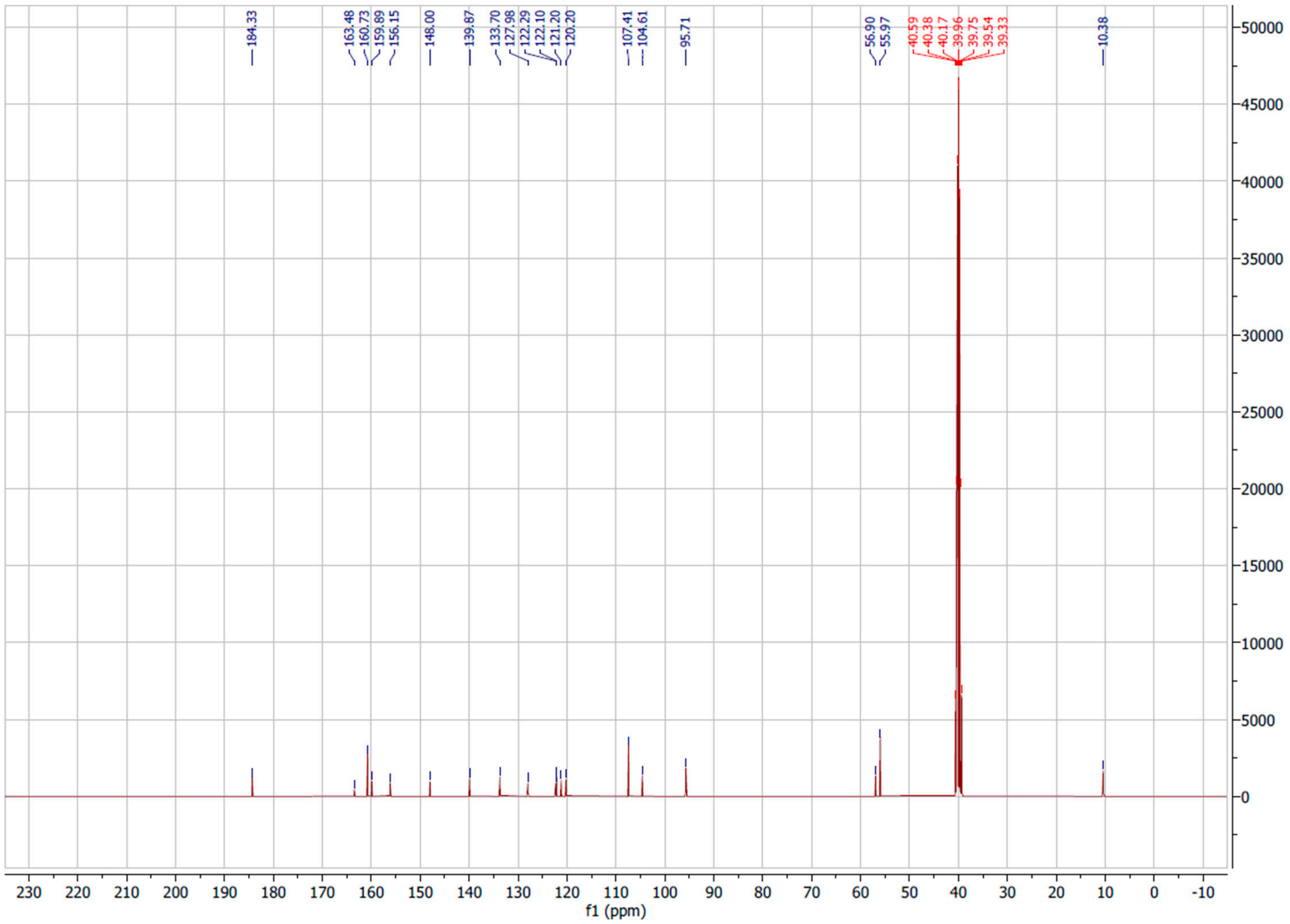

^{13}C-NMR spectra of compound **6d**

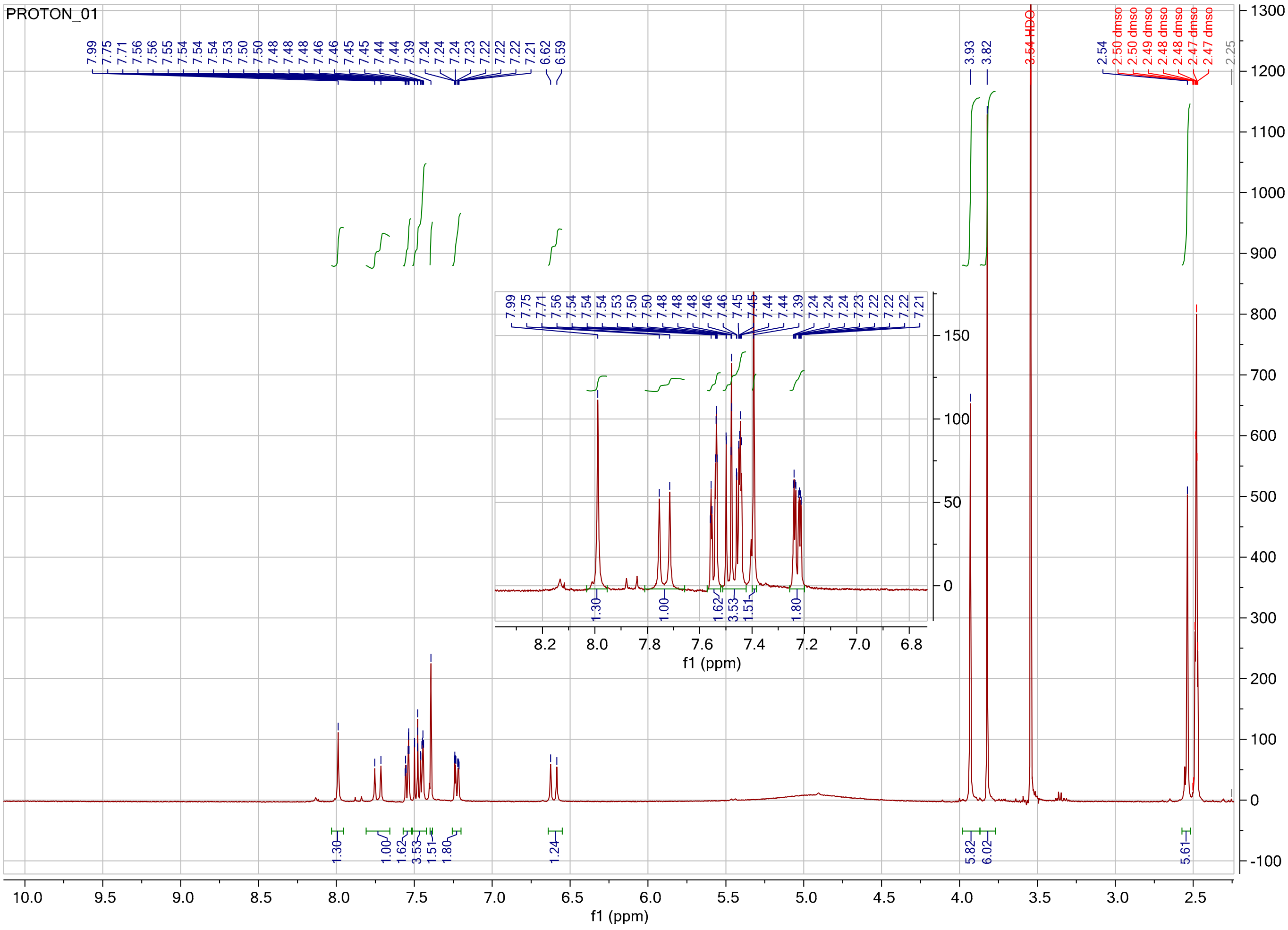

^{1}H-NMR spectra of compound **6e**

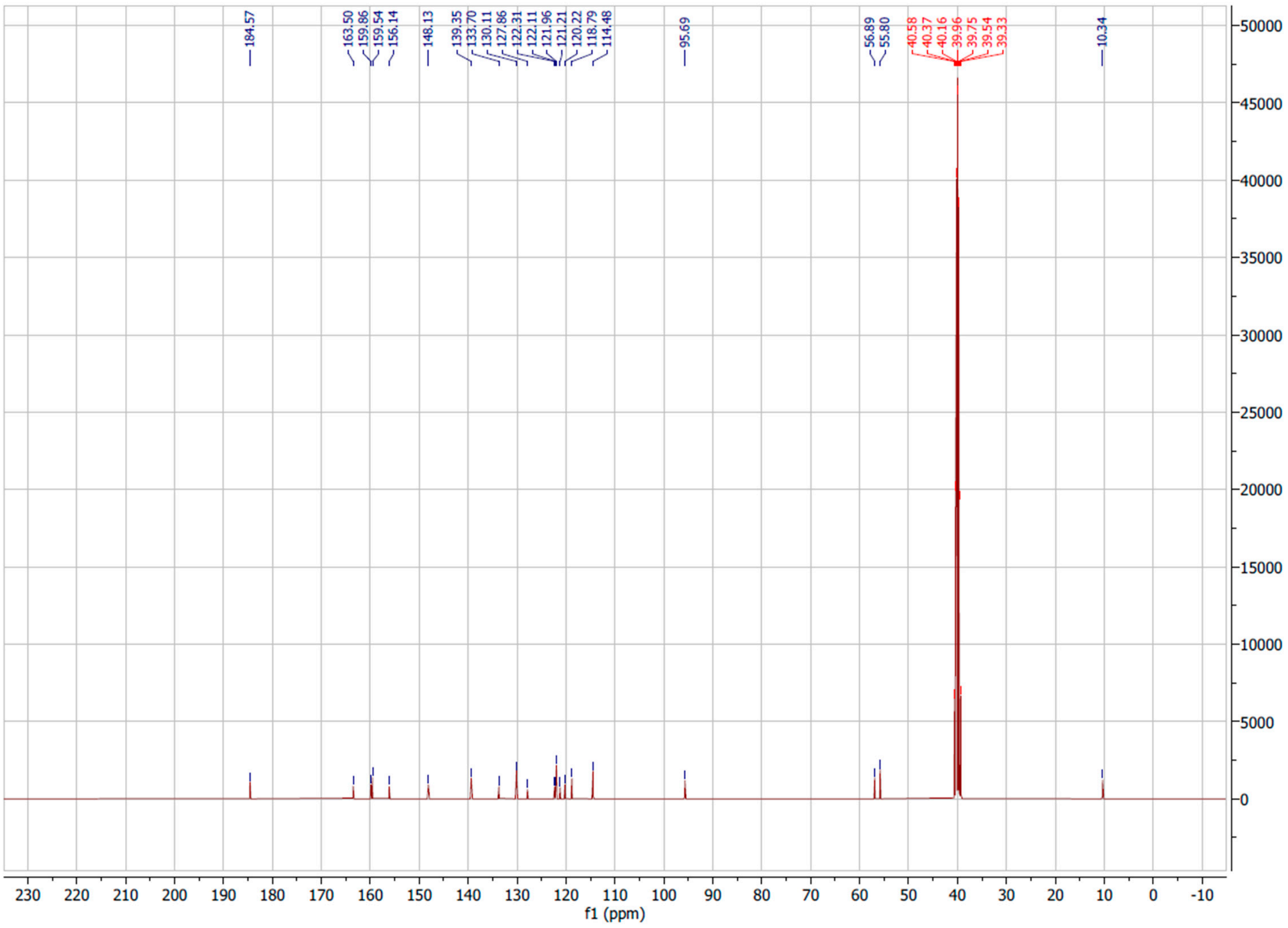

^{13}C-NMR spectra of compound **6e**

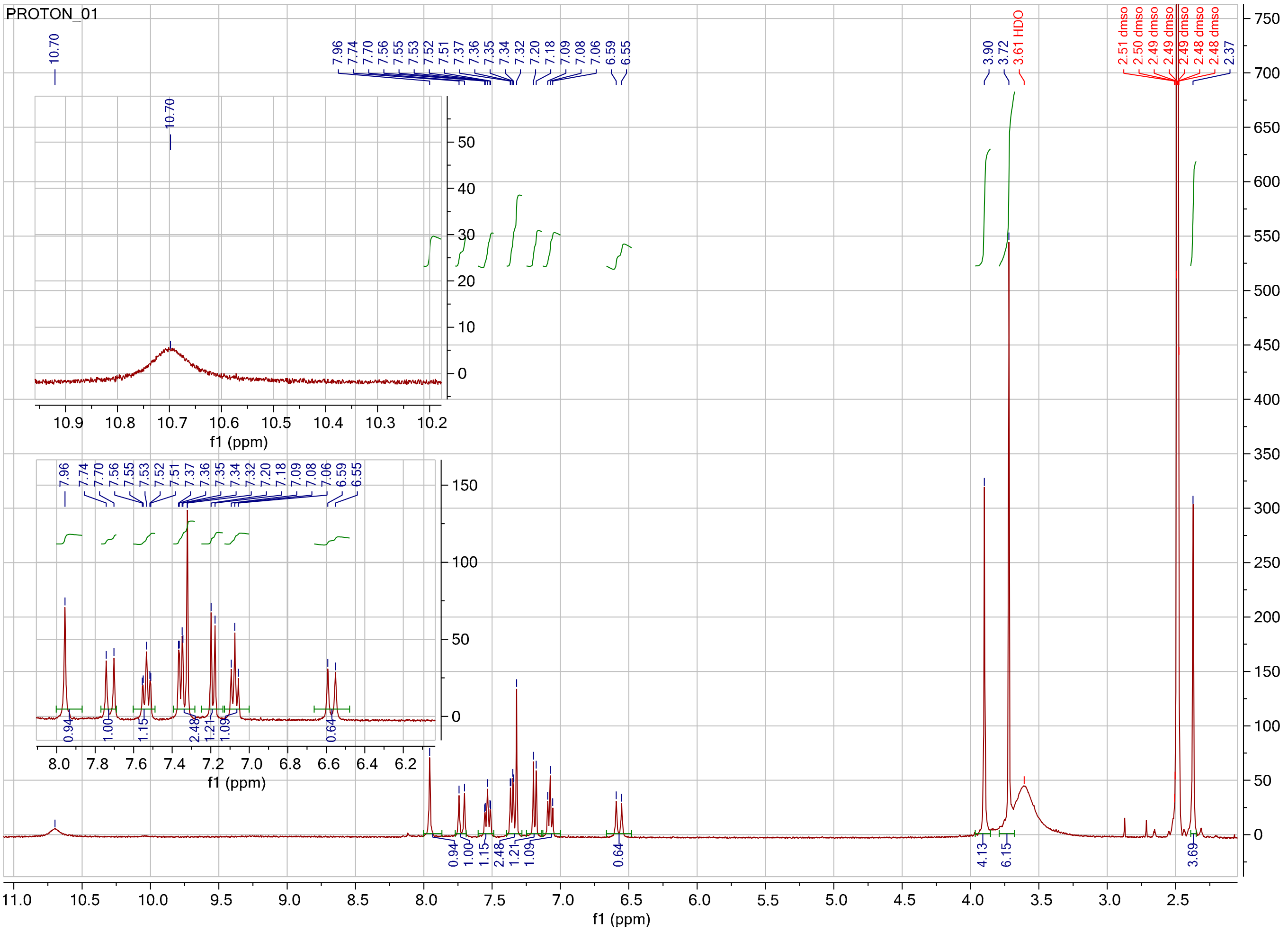

^{1}H-NMR spectra of compound **6f**

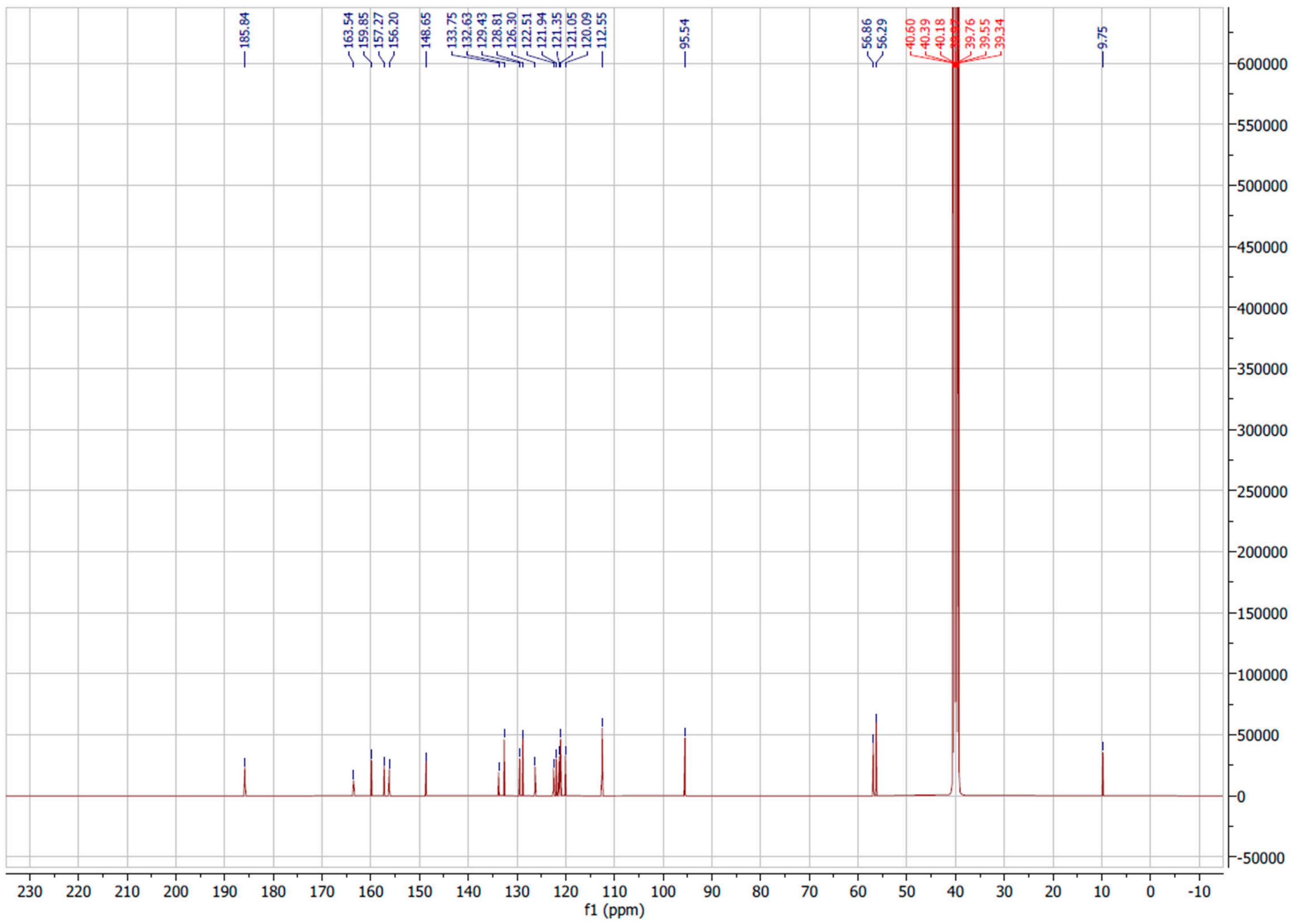

^{13}C-NMR spectra of compound **6f**

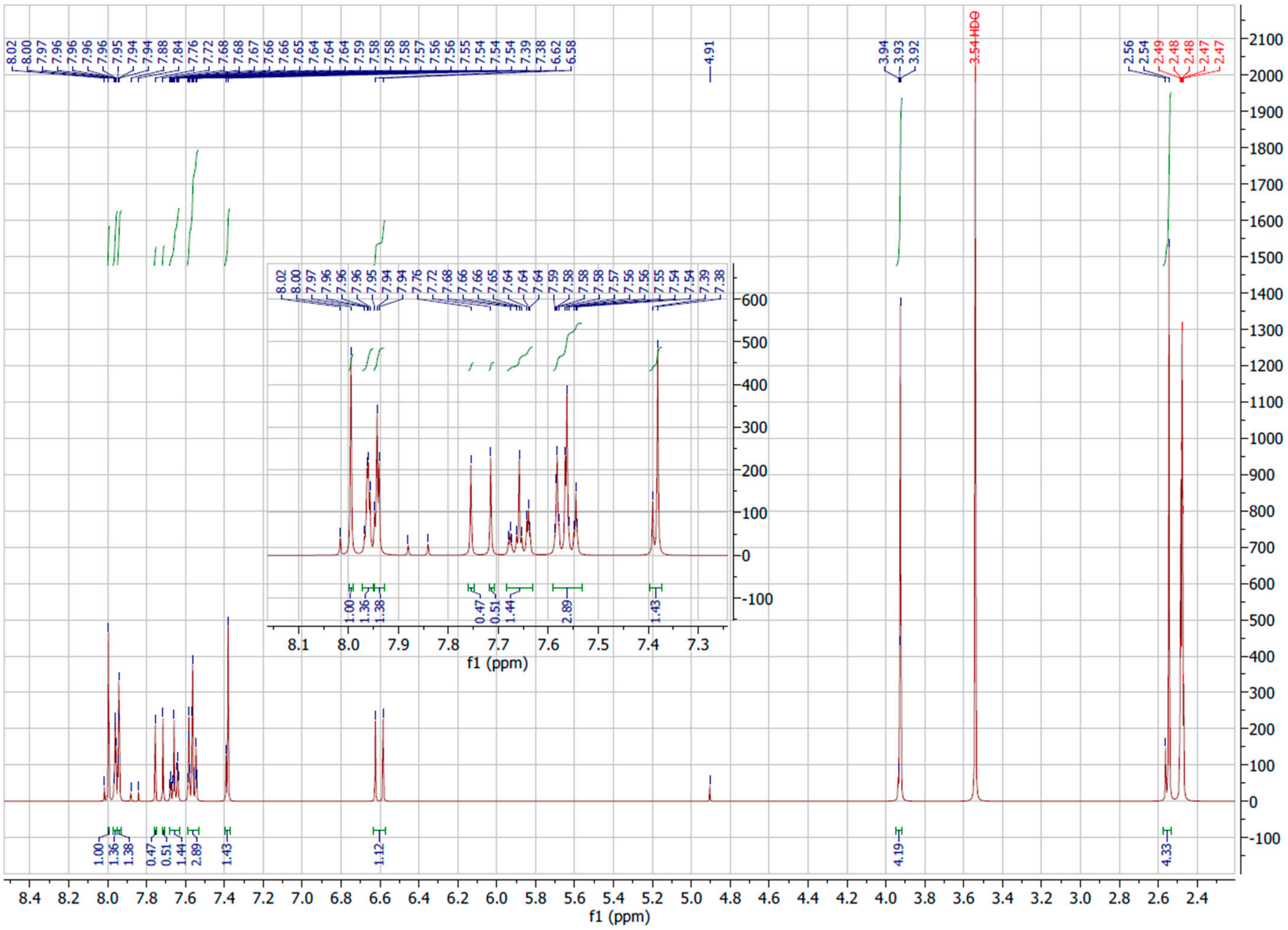

^{1}H-NMR spectra of compound **6g**

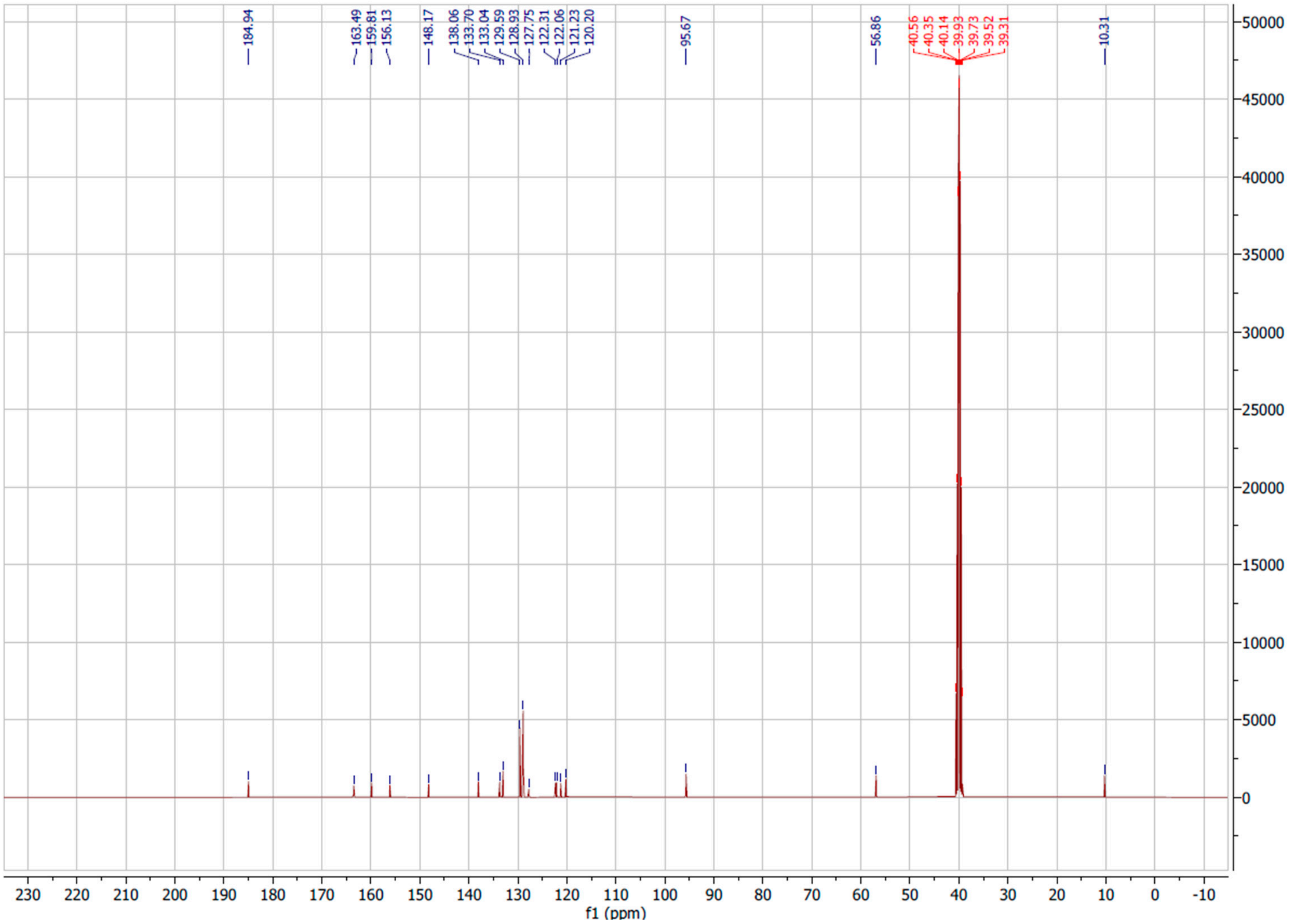

^{13}C-NMR spectra of compound **6g**

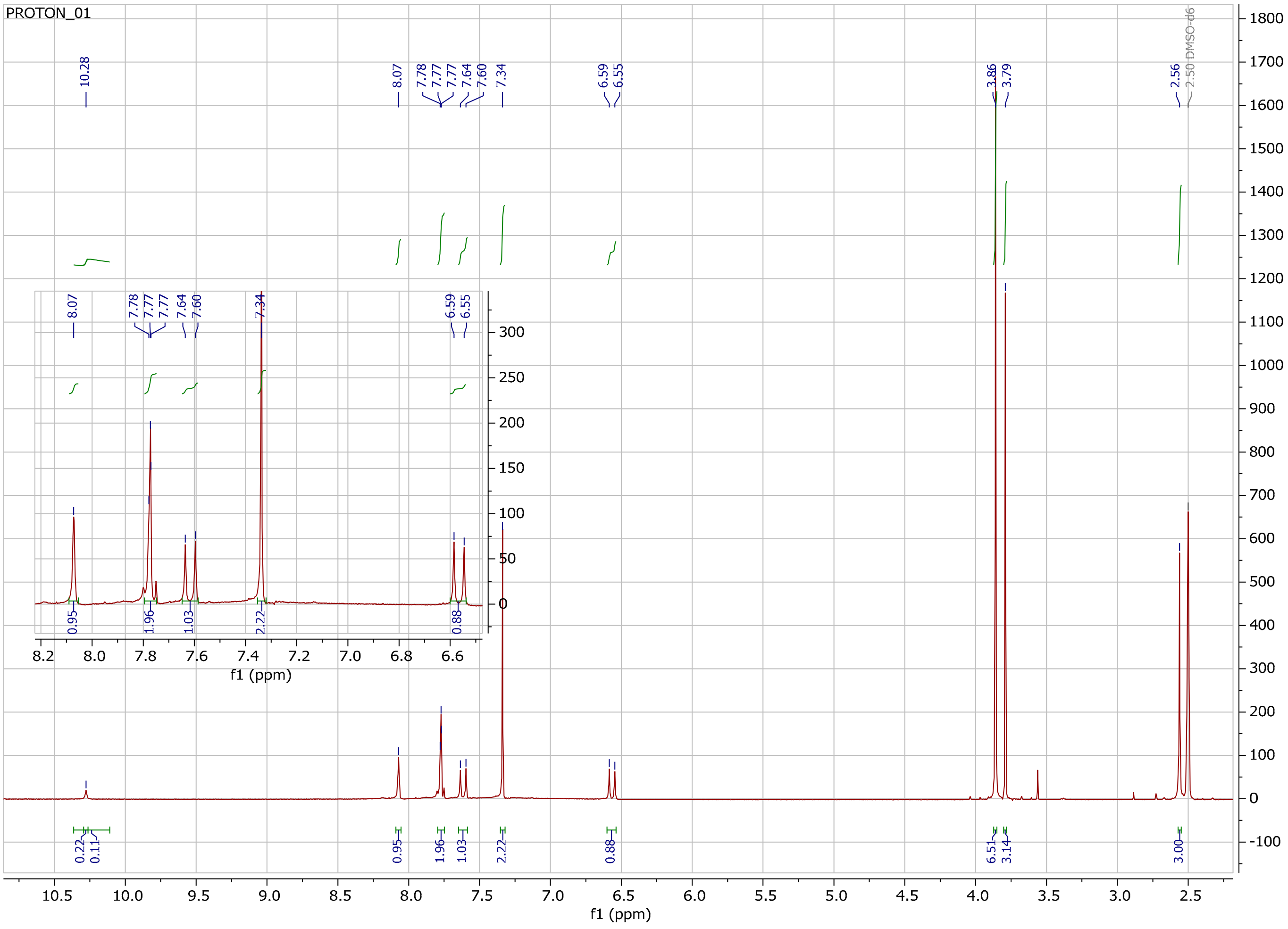

^{1}H-NMR spectra of compound **6h**

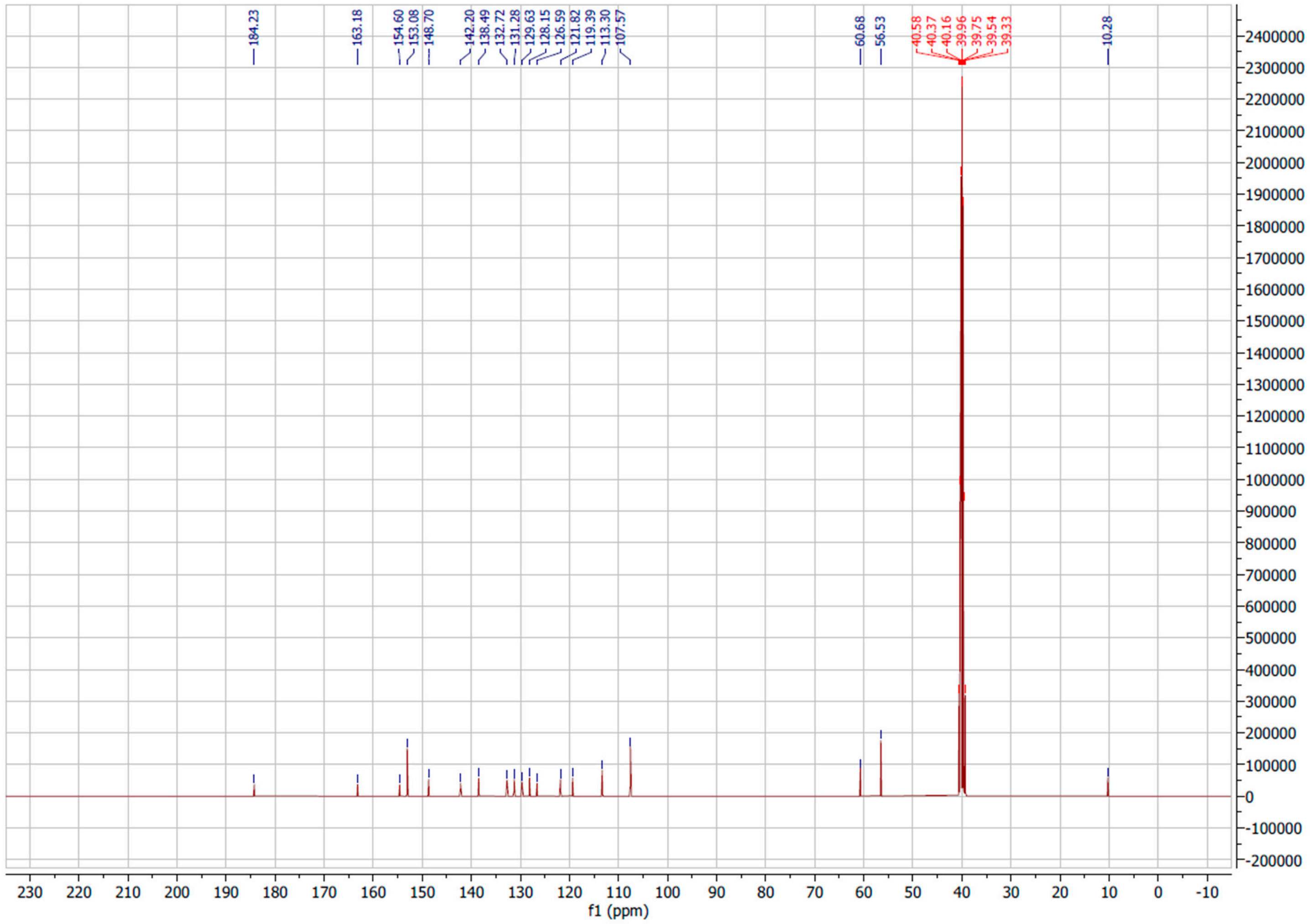

^{13}C-NMR spectra of compound **6h**

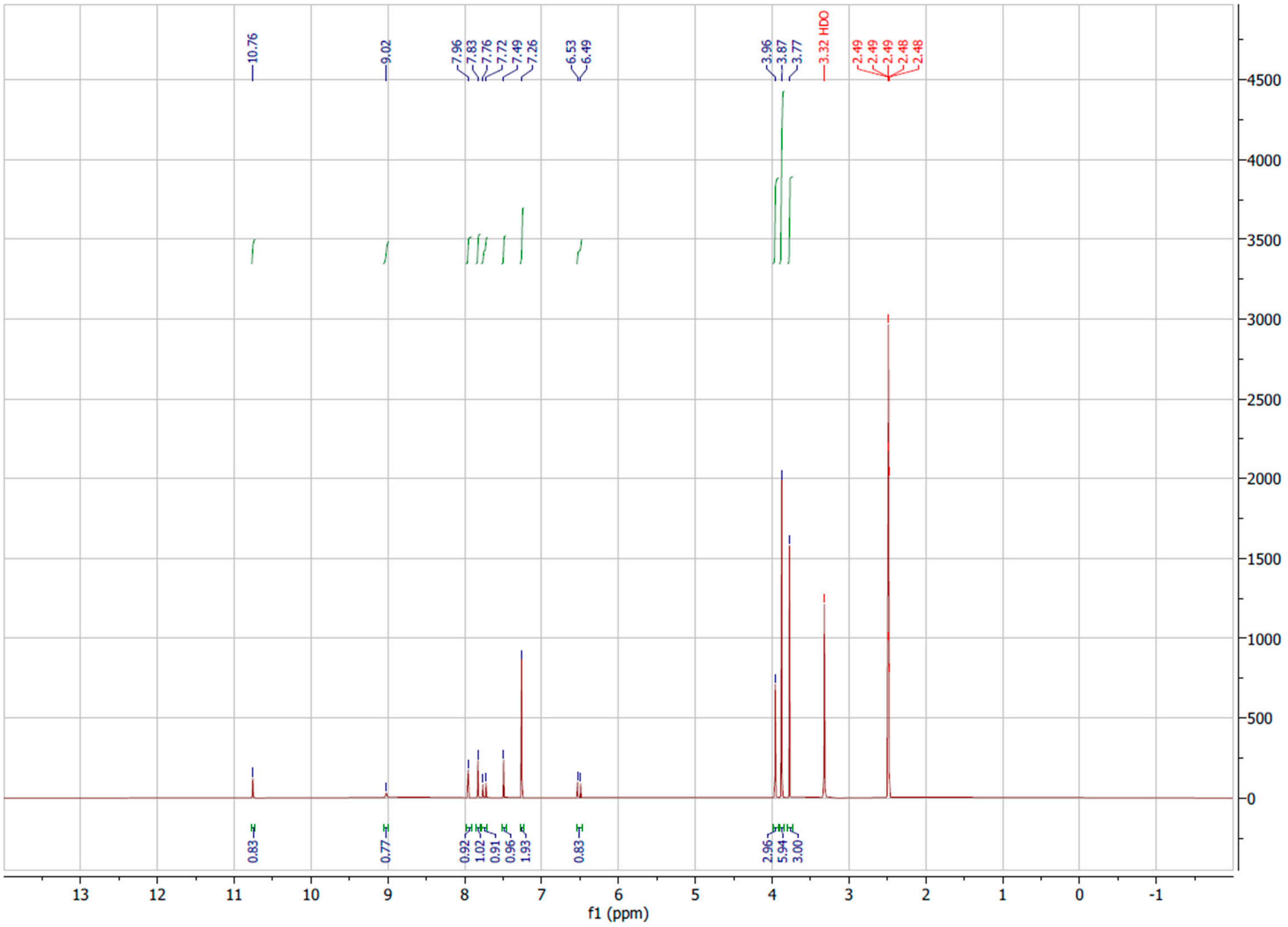

^{1}H-NMR spectra of compound **6i**

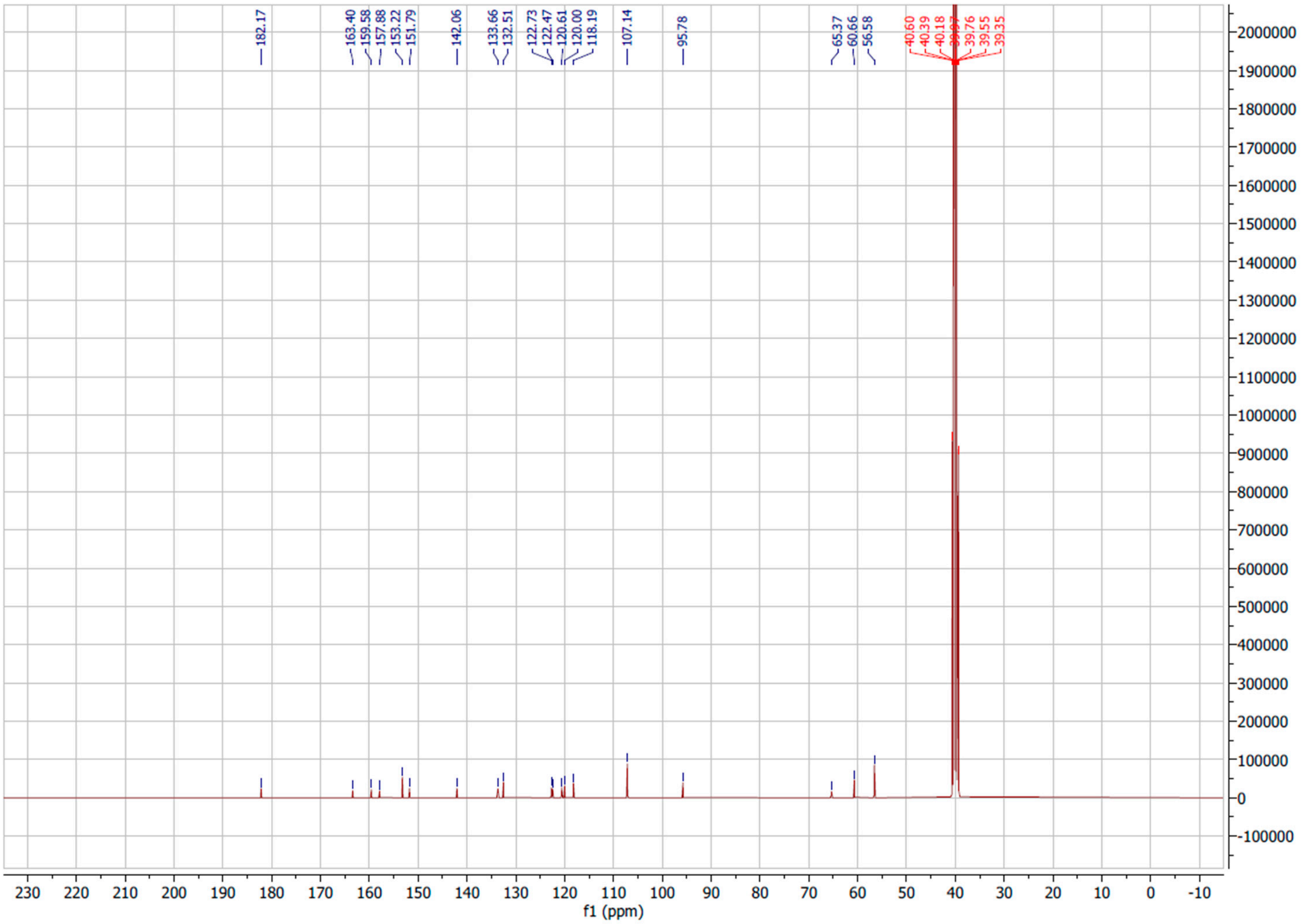

^{13}C-NMR spectra of compound **6i**

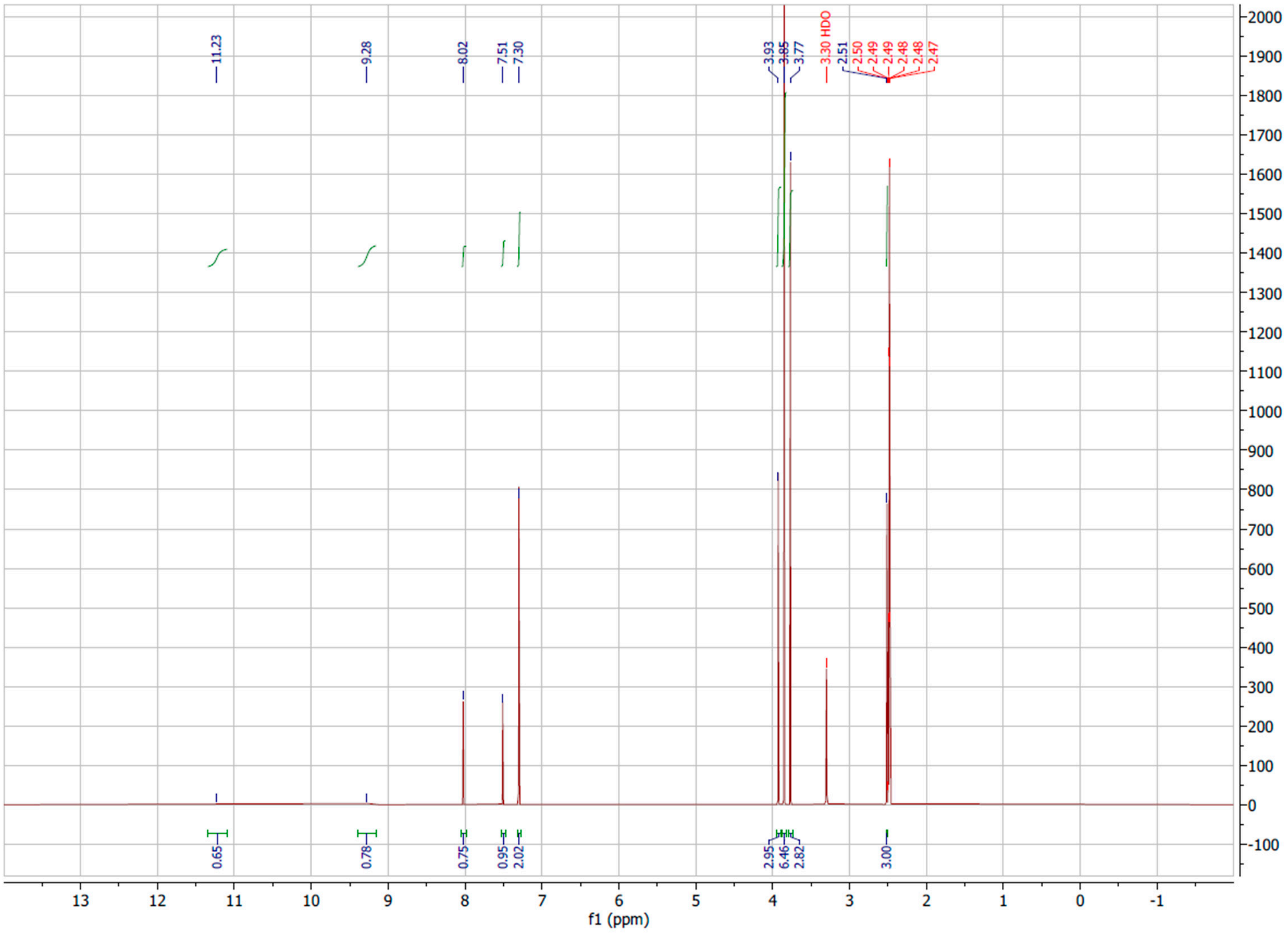

^{1}H-NMR spectra of compound **11a**

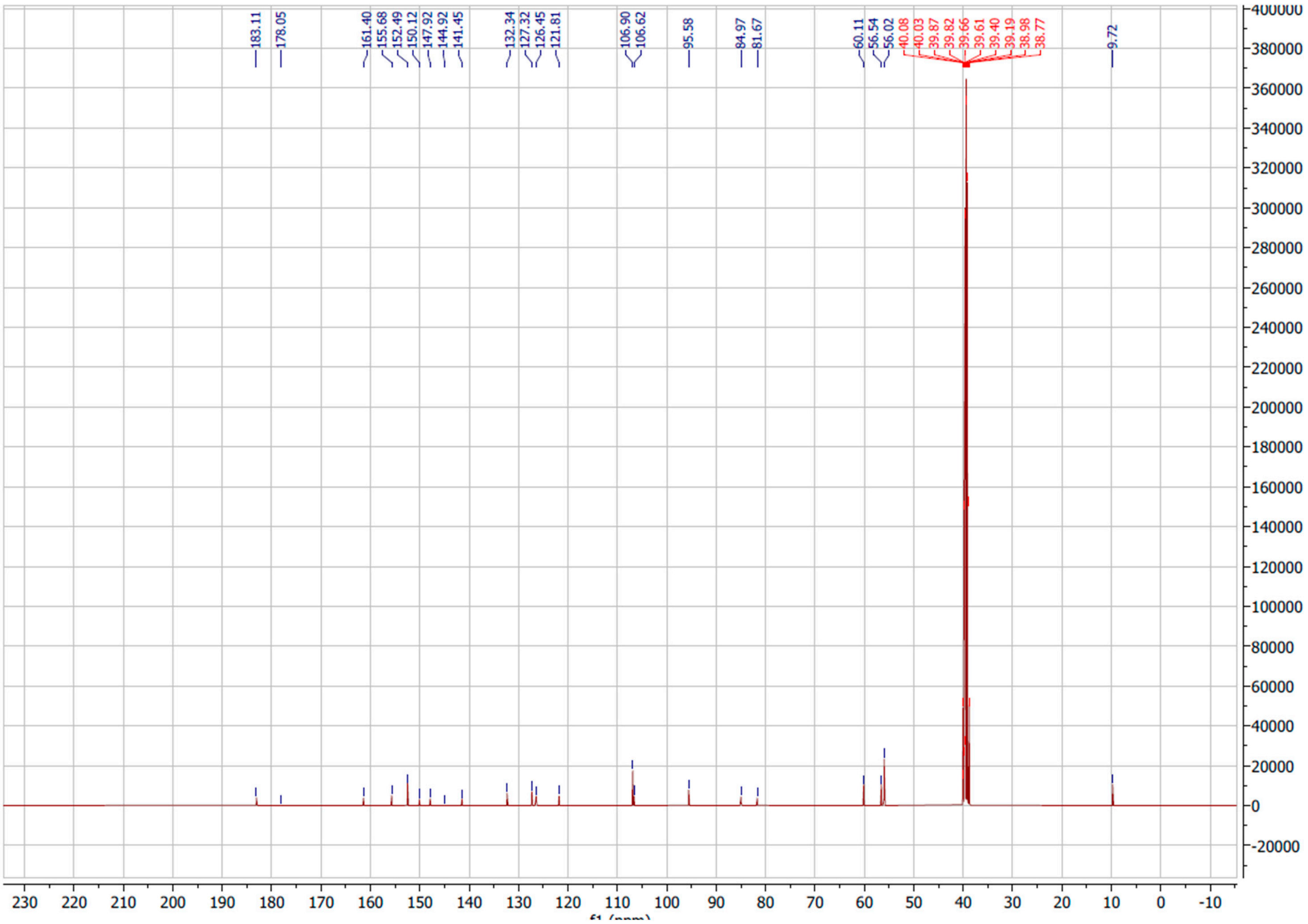

^{13}C-NMR spectra of compound **11a**

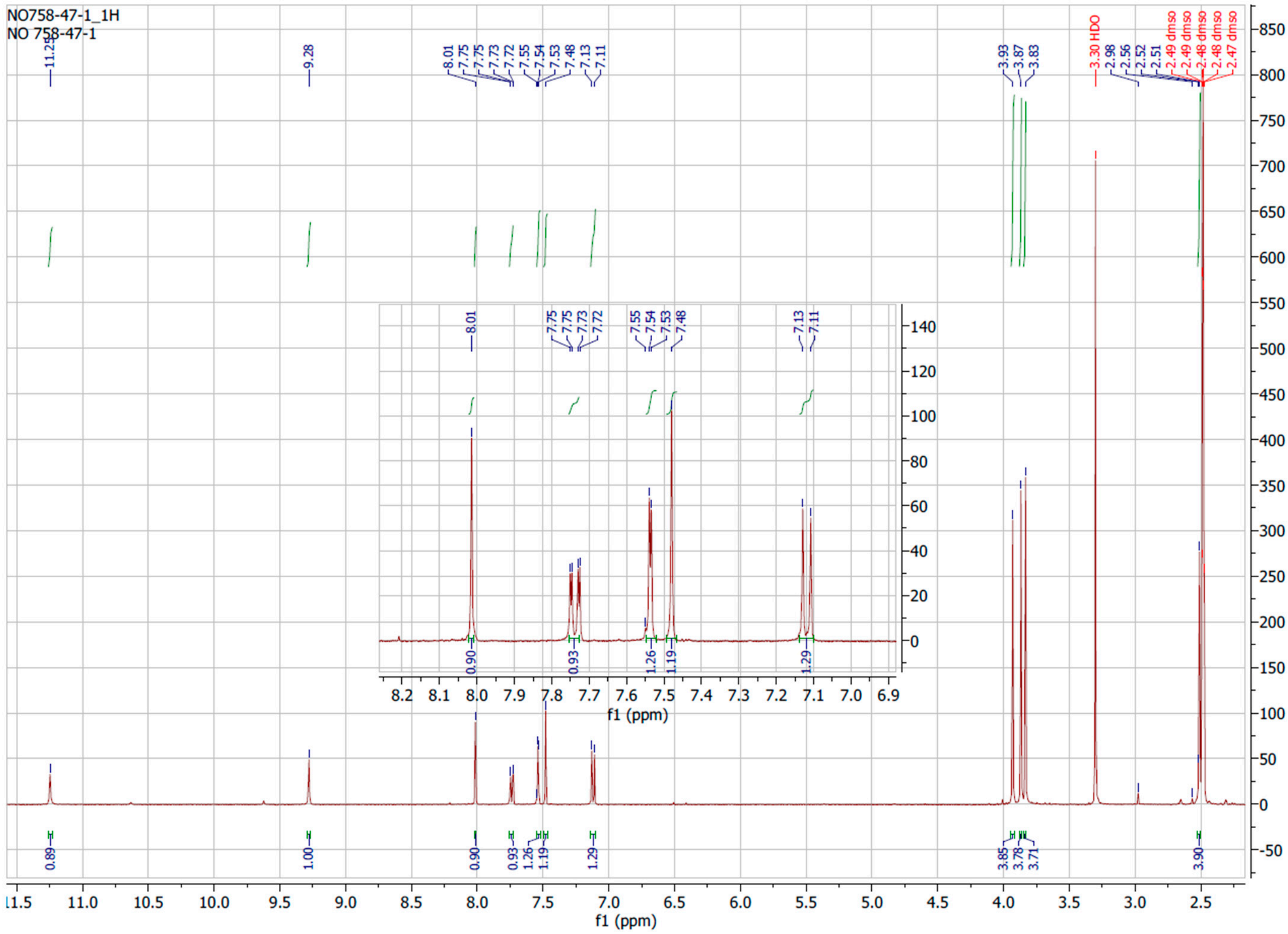

^{1}H-NMR spectra of compound **11b**

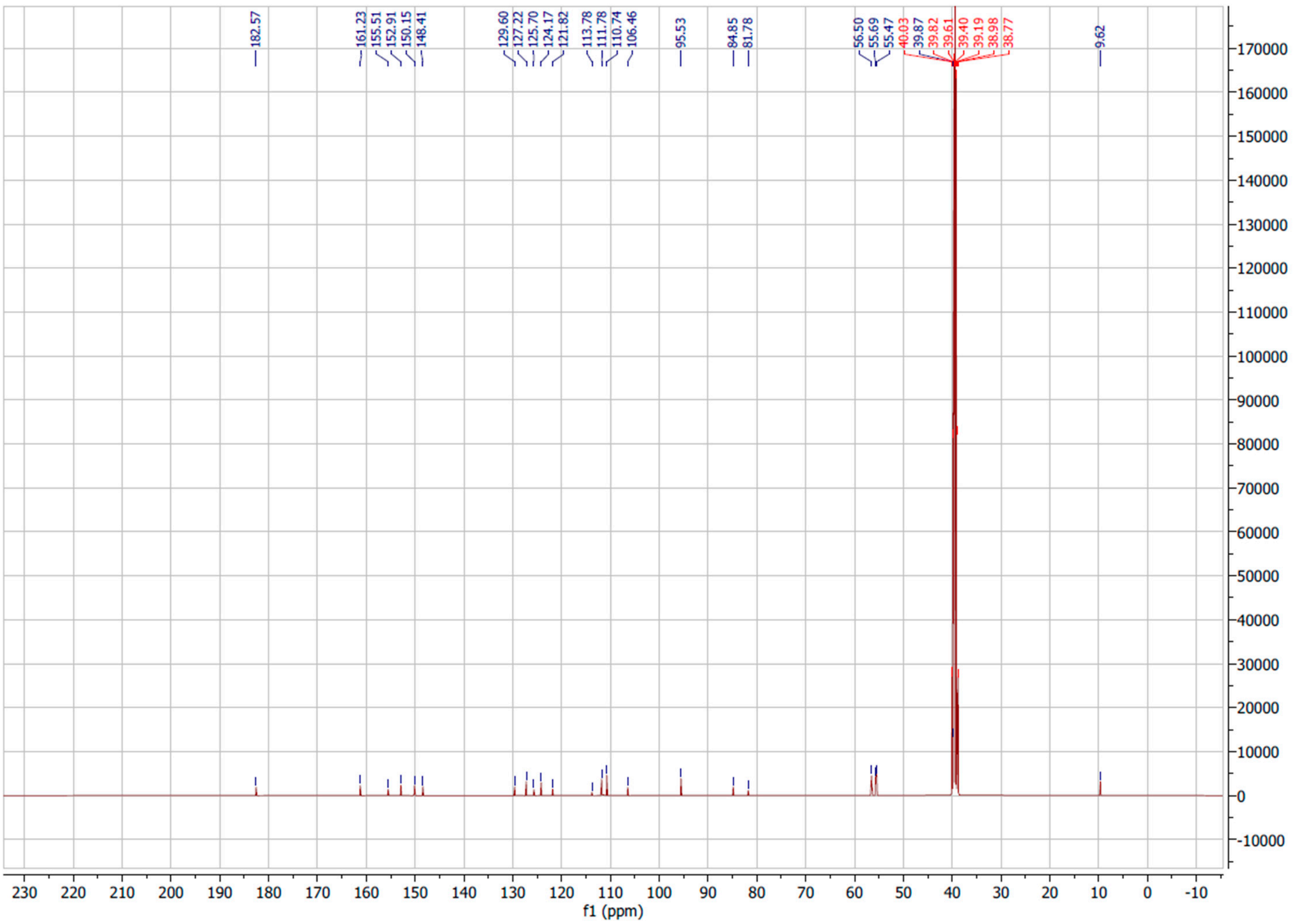

^{13}C-NMR spectra of compound **11b**

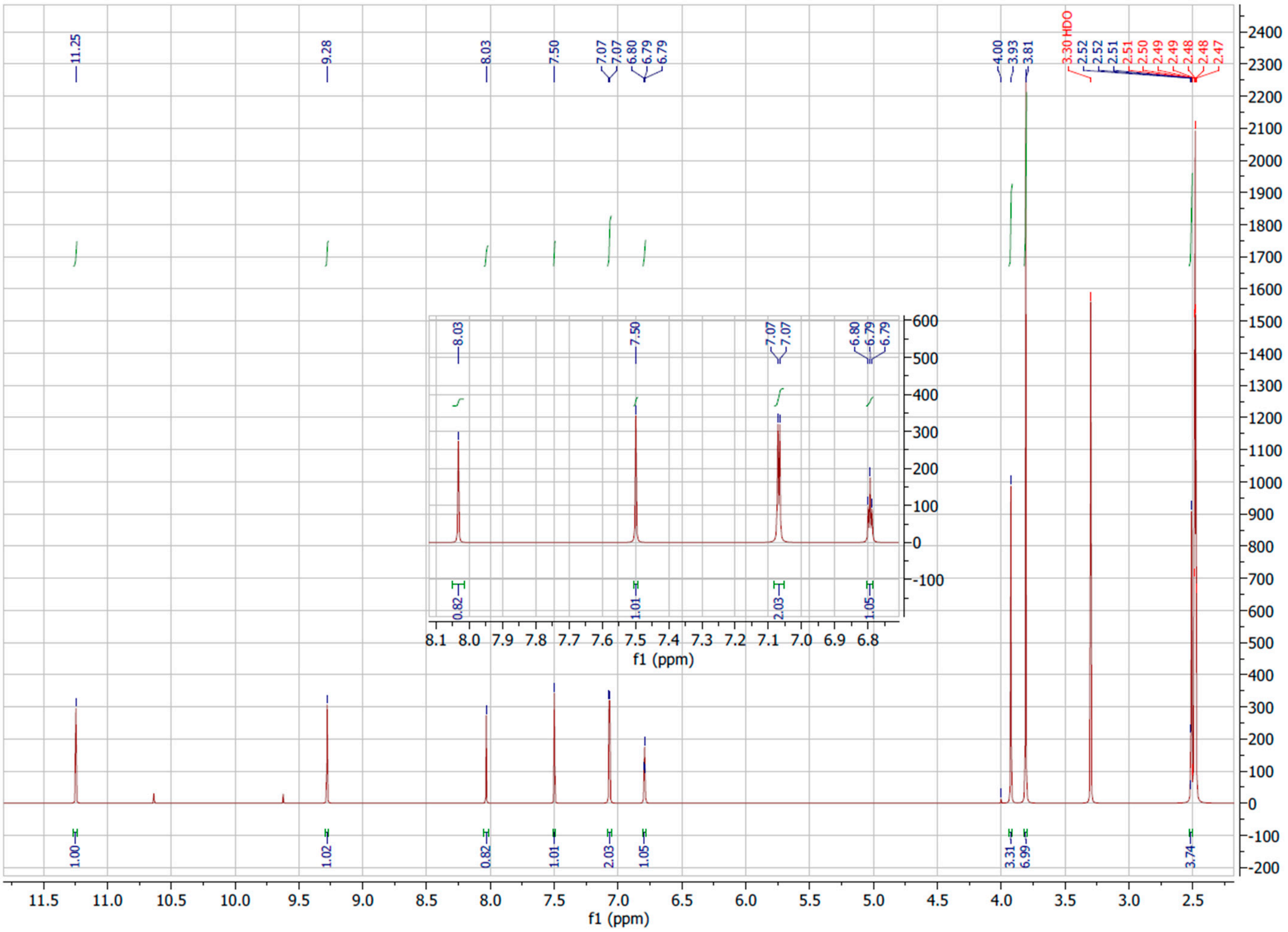

^{1}H-NMR spectra of compound **11c**

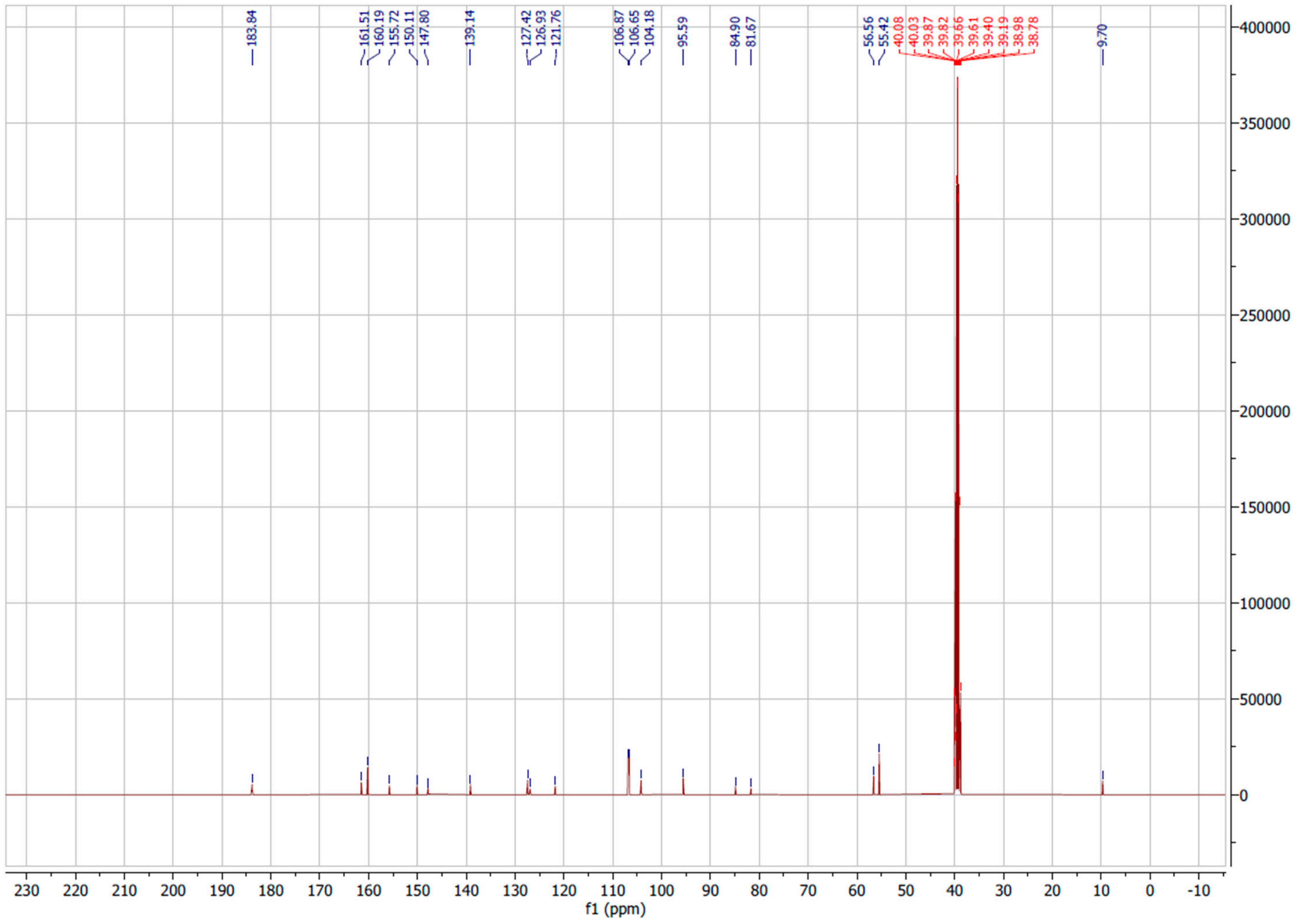

^{13}C-NMR spectra of compound **11c**

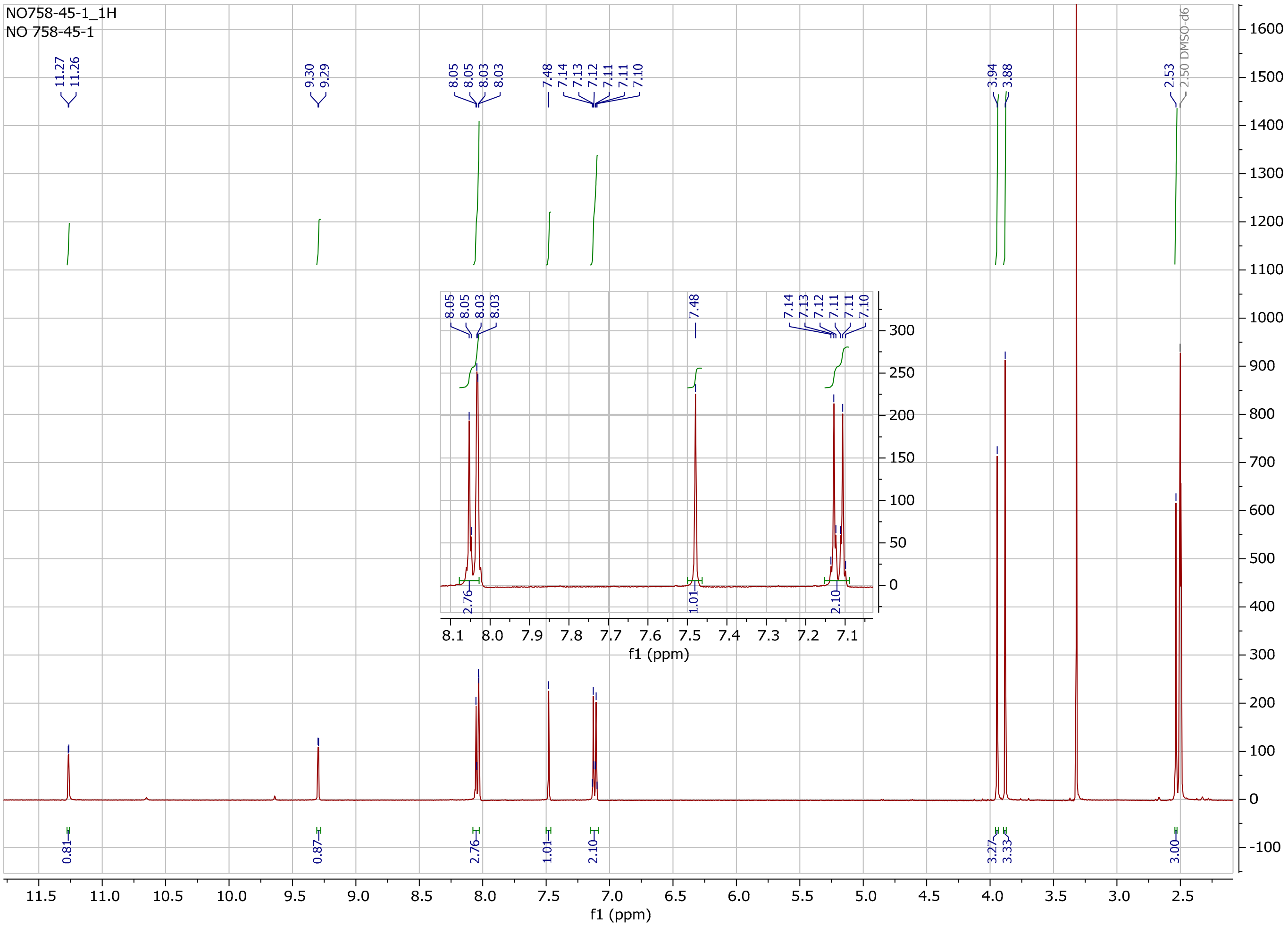

^{1}H-NMR spectra of compound **11d**

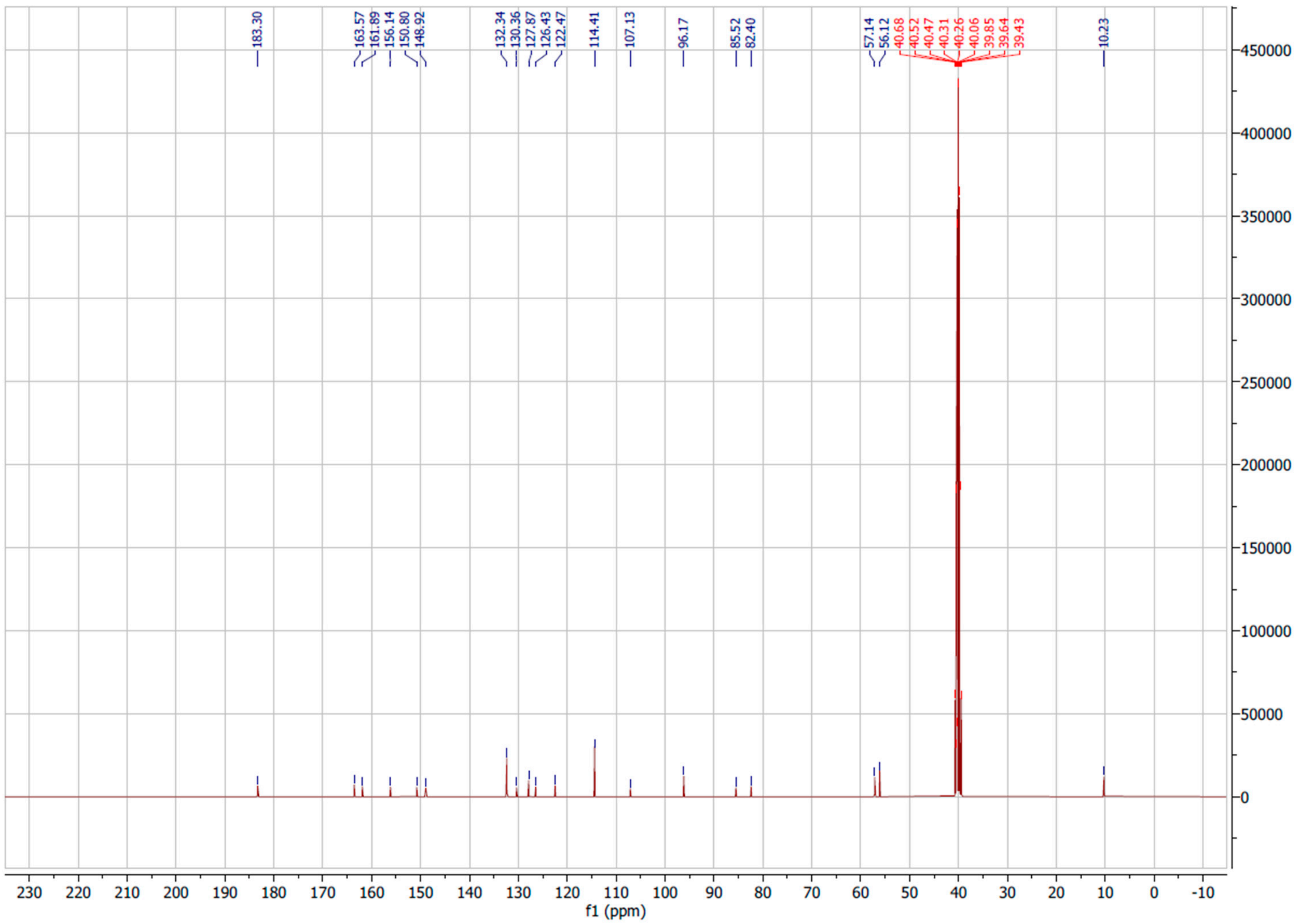

^{13}C-NMR spectra of compound **11d**

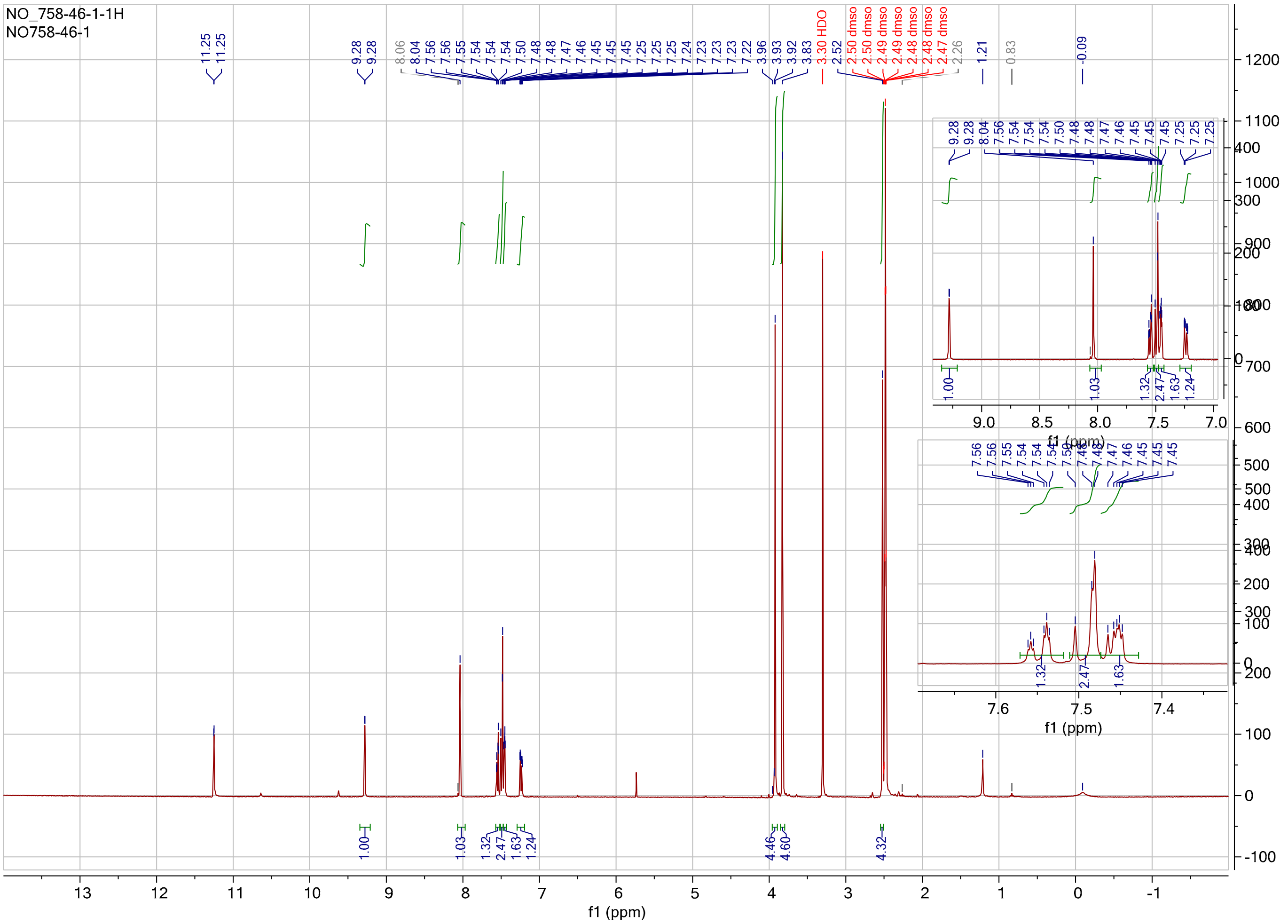

^{1}H-NMR of compound **11e**

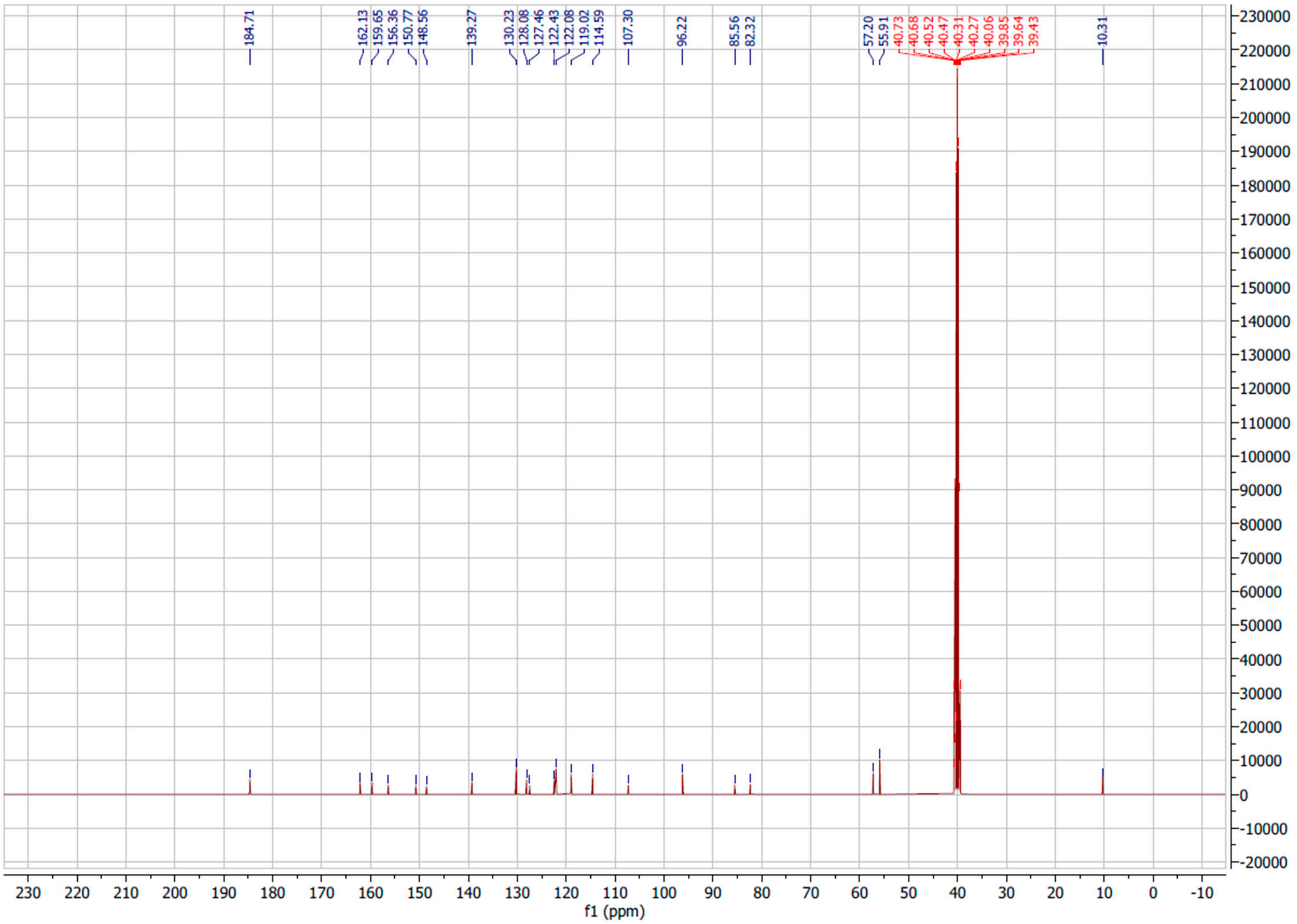

^{1}H-NMR and ^{13}C-NMR spectra of compound **11e**

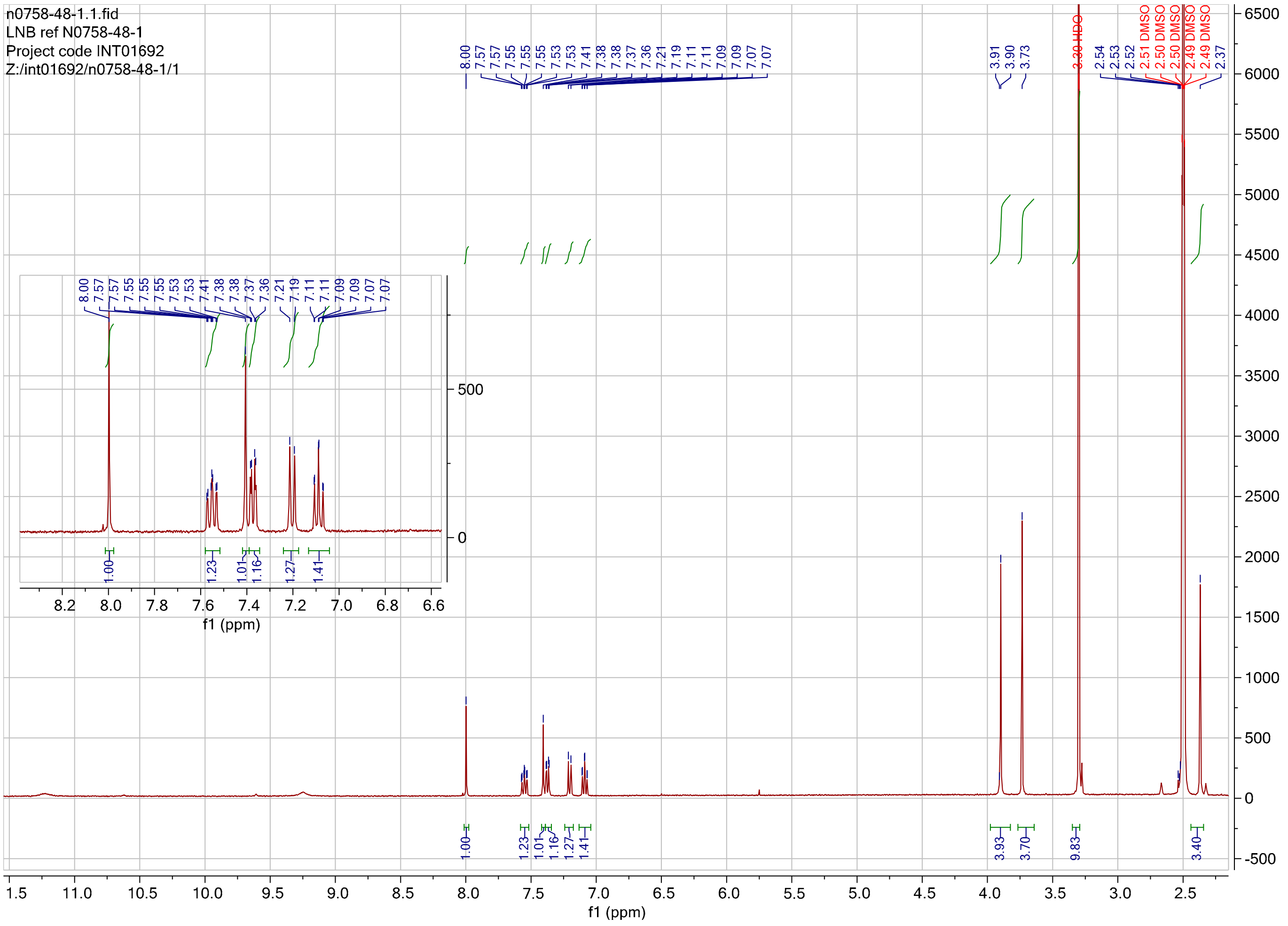

^{1}H-NMR spectra of compound **11f**

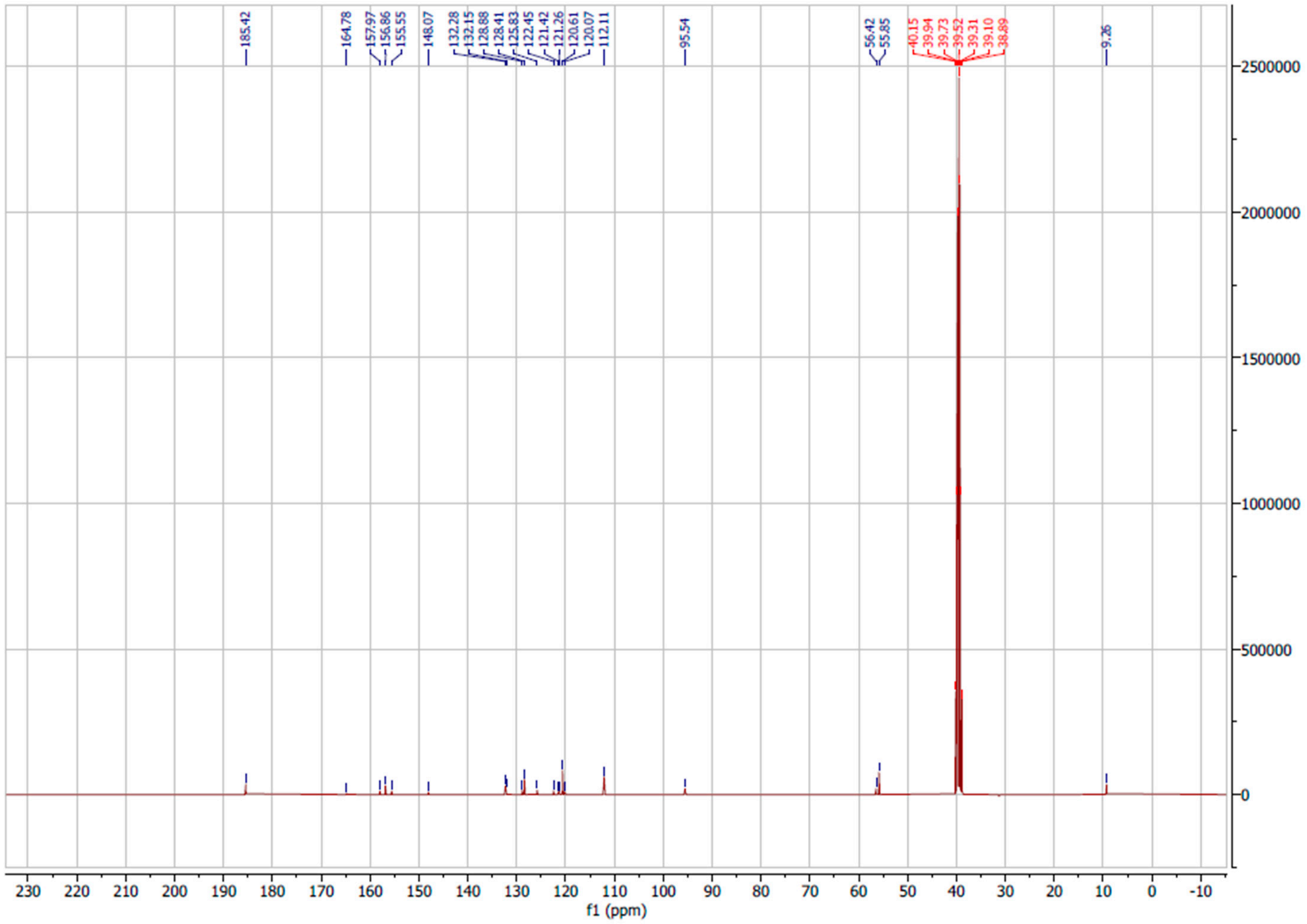

^{13}C-NMR spectra of compound **11f**

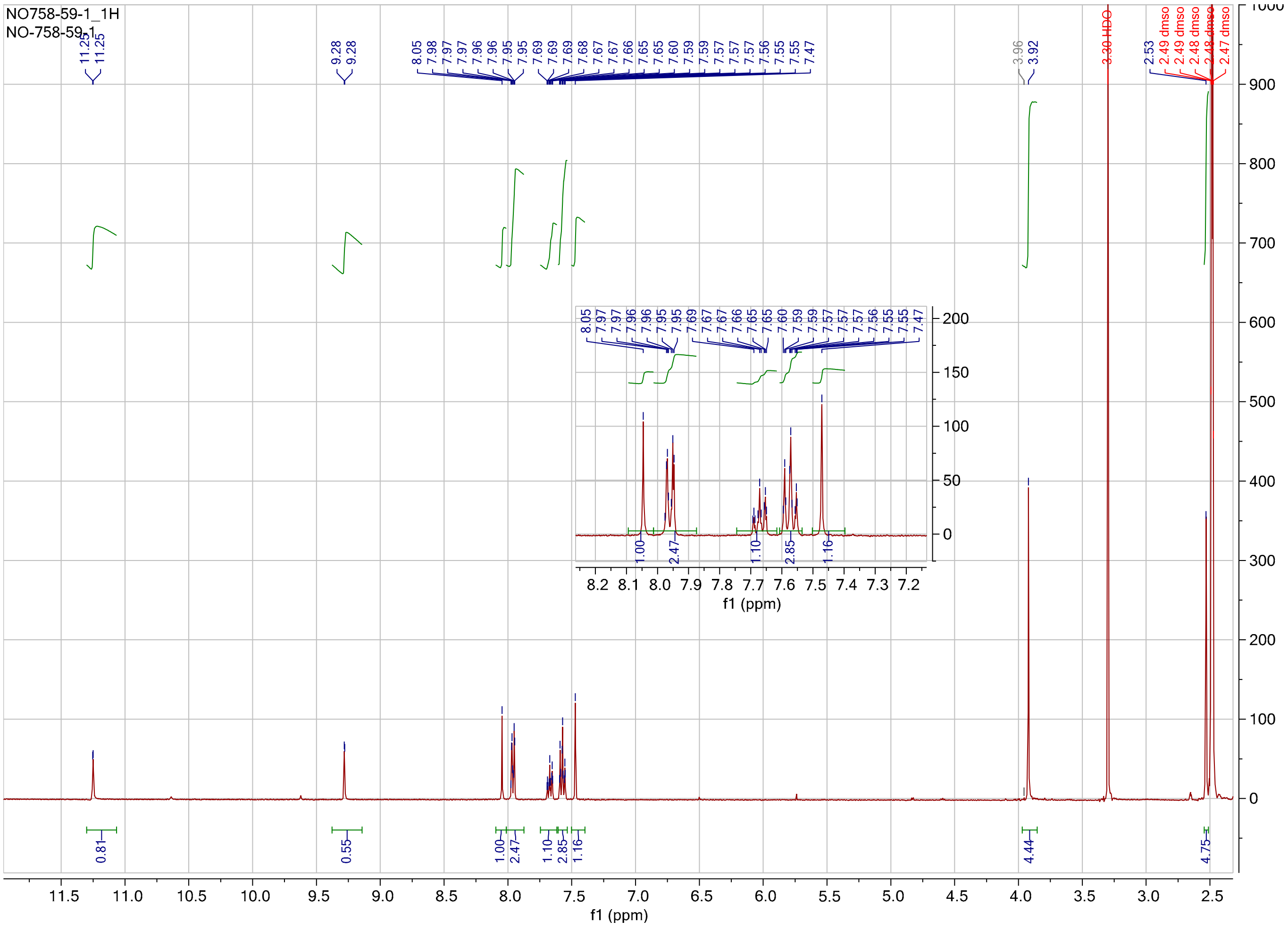

^{1}H-NMR spectra of compound **11g**

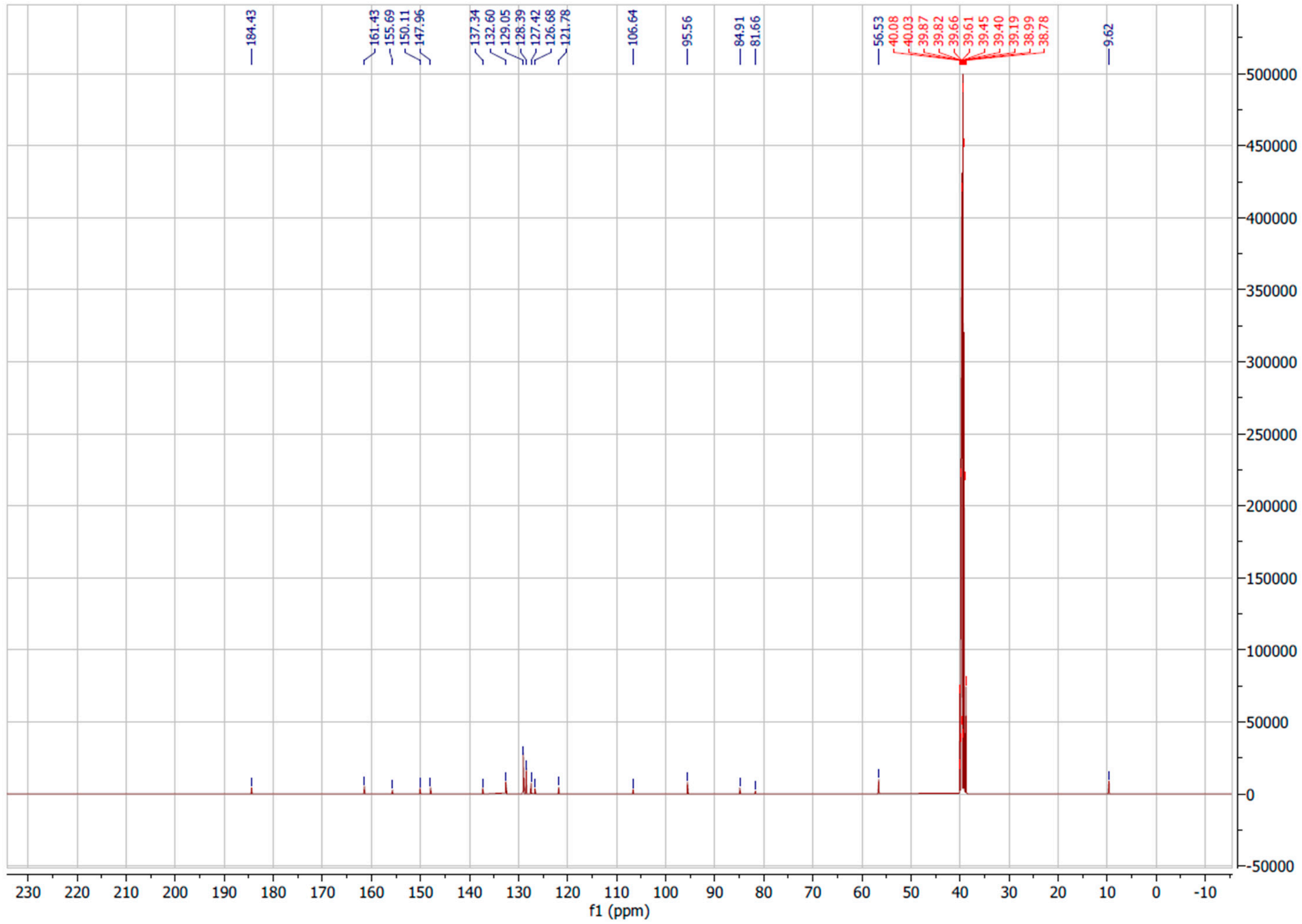

^{13}C-NMR spectra of compound **11g**

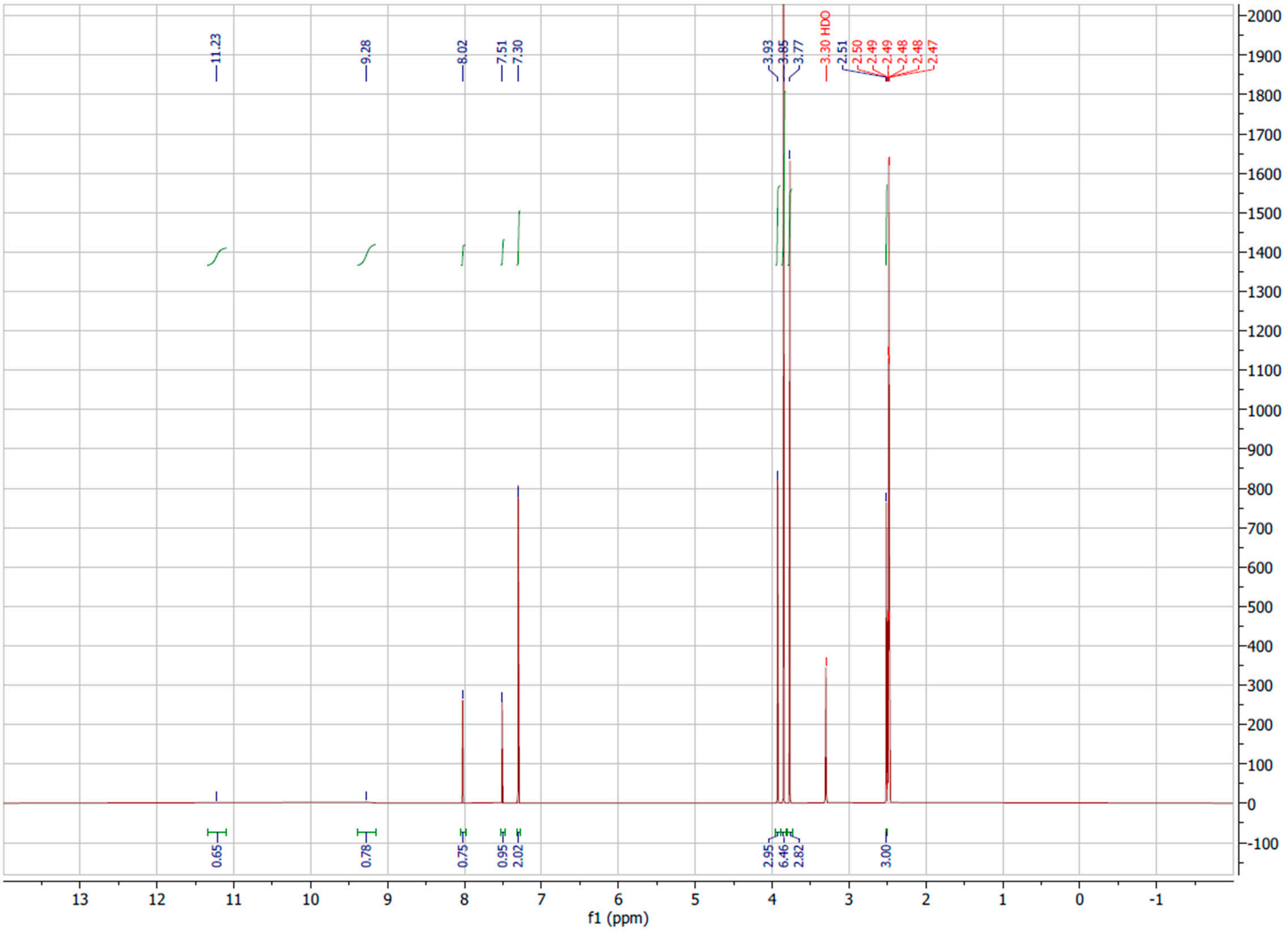

^{1}H-NMR spectra of compound **11h**

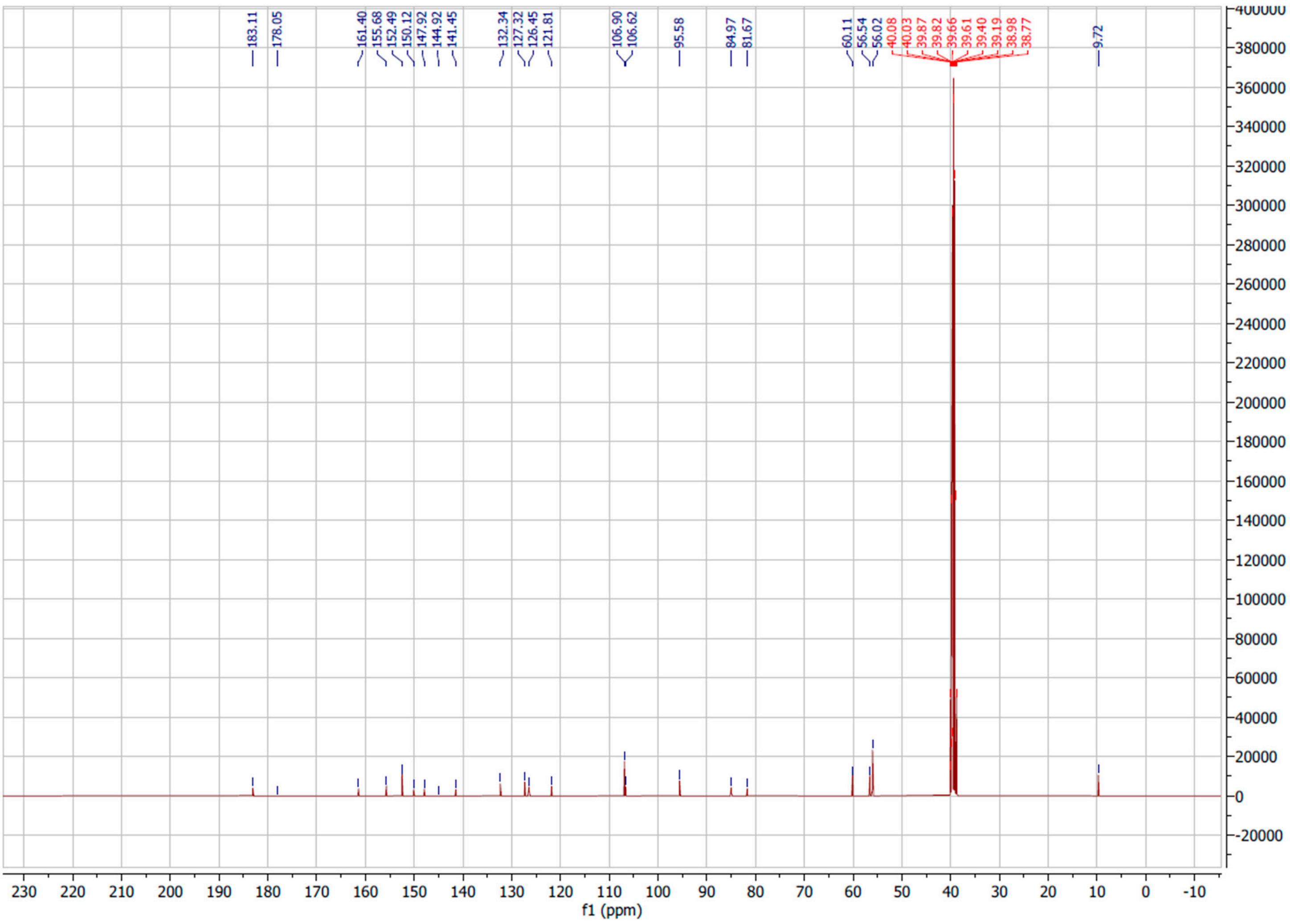

^{13}C-NMR spectra of compound **11h**

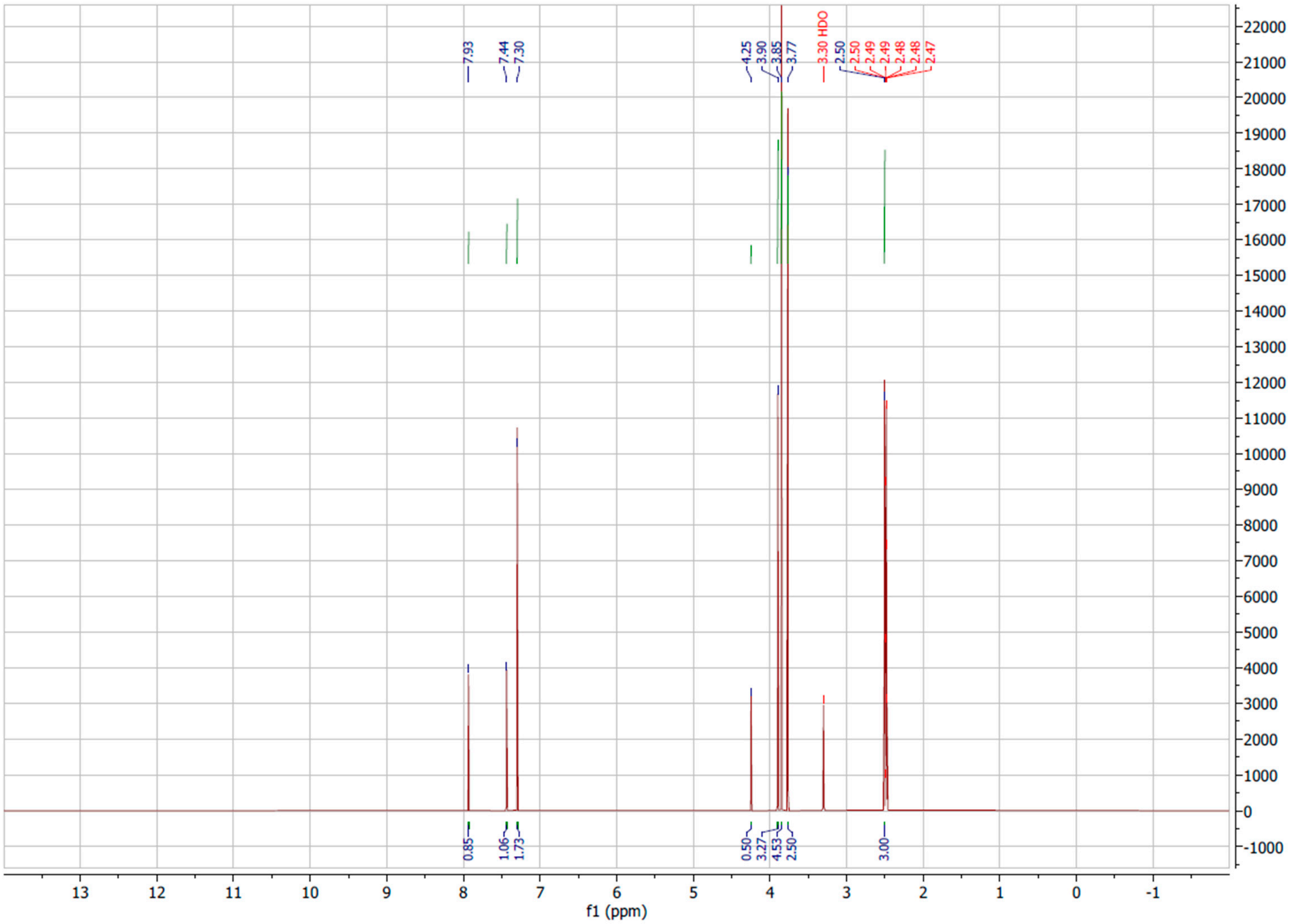

^{1}H-NMR spectra of compound **8**

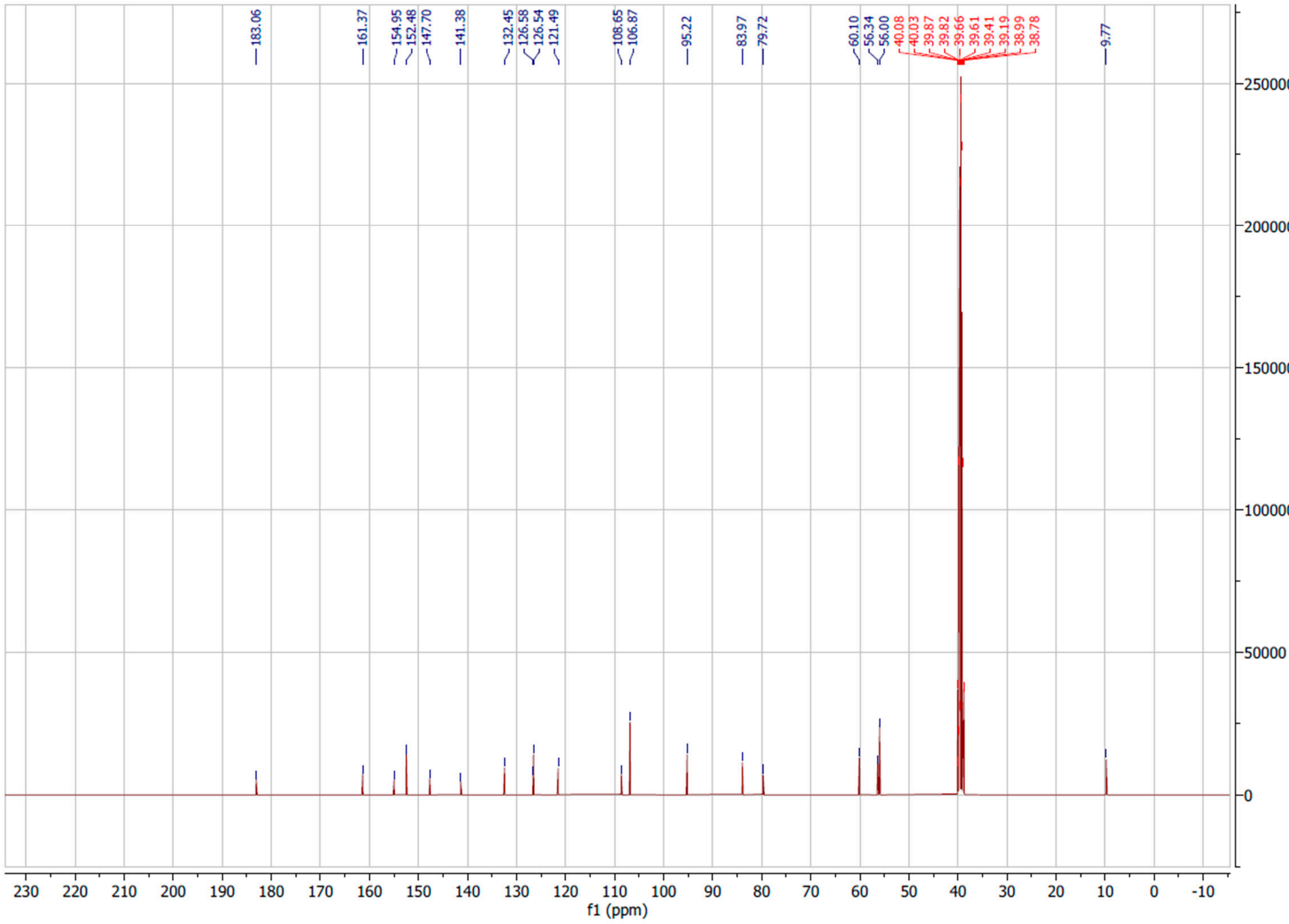

^{13}C-NMR spectra of compound **8**
